# Why did hunting weapon design change at Abri Pataud? Lithic use-wear data on armature use and hafting around 24,000–22,000 BP

**DOI:** 10.1371/journal.pone.0262185

**Published:** 2022-01-14

**Authors:** Noora Taipale, Laurent Chiotti, Veerle Rots

**Affiliations:** 1 TraceoLab / Prehistory, University of Liège, Liège, Belgium; 2 Département Homme et Environnement, Muséum national d’Histoire naturelle, UMR 7194 HNHP, Abri Pataud, France; 3 Maître de Recherches du FNRS, University of Liège, Liège, Belgium; Max Planck Institute for the Science of Human History, GERMANY

## Abstract

Projectile technology is commonly viewed as a significant contributor to past human subsistence and, consequently, to our evolution. Due to the allegedly central role of projectile weapons in the food-getting strategies of Upper Palaeolithic people, typo-technological changes in the European lithic record have often been linked to supposed developments in hunting weaponry. Yet, relatively little reliable functional data is currently available that would aid the detailed reconstruction of past weapon designs. In this paper, we take a use-wear approach to the backed tool assemblages from the Recent and Final Gravettian layers (Levels 3 and 2) of Abri Pataud (Dordogne, France). Our use of strict projectile identification criteria relying on combinations of low and high magnification features and our critical view of the overlap between production and use-related fractures permitted us to confidently identify a large number of used armatures in both collections. By isolating lithic projectiles with the strongest evidence of impact and by recording wear attributes on them in detail, we could establish that the hunting equipment used during the Level 3 occupations involved both lithic weapon tips and composite points armed with lithic inserts. By contrast, the Level 2 assemblage reflects a heavy reliance on composite points in hunting reindeer and other game. Instead of an entirely new weapon design, the Level 2 collection therefore marks a shift in weapon preferences. Using recent faunal data, we discuss the significance of the observed diachronic change from the point of view of prey choice, seasonality, and social organisation of hunting activities. Our analysis shows that to understand their behavioural significance, typo-technological changes in the lithic record must be viewed in the light of functional data and detailed contextual information.

## 1. Introduction

Palaeolithic projectile technology has been much discussed and debated in the recent decades due to the central place it has been assigned in the evolutionary history of our species. The advances in the manufacture and use of hunting weaponry, such as the introduction of composite weapons and long-range weapon systems, have been viewed as having significantly contributed to the success of early *Homo sapiens* and their dispersal to different parts of the world [e.g. 1–3; for a critical discussion, see 4]. Major technological shifts seen in the lithic record, particularly starting from the beginning of the Upper Palaeolithic, have often been associated with the development of projectile weaponry [e.g. 5] and changes in lithic technology in the more recent periods have habitually been seen as driven by adjustments to weapon design and lithic armature production [see 6 for a discussion]. In addition, the distinct shapes of lithic armatures and the chronological and regional variability they present have guaranteed them a central place in typology-based models of cultural variation [7, 8]. Palaeolithic archaeology has thus in many ways prioritised projectile elements over other tool categories and given them a high status in both explanatory models and classificatory systems.

Despite the privileged position projectiles enjoy in archaeological research, reliable functional data that would in detail describe how weapons were exactly constructed and used remains scarce. While the oldest known lithic tools that bear trace combinations suggestive of projectile use currently date to around 200 to 250 kya [9, 10; for critical reviews of older claims, see 11–13], relatively little is known about the subsequent developments in hunting weaponry and their implications for human subsistence and evolution. This is largely due to the fact that despite considerable experimental and analytical efforts, secure identification of archaeological lithic projectiles remains a challenge [8, 11], and the available datasets are not comparable to each other in terms of methodological rigour. Shifts in armature typology therefore remain poorly understood in terms of weapon design and hunting tactics. Fully preserved Palaeolithic weapons are exceptional finds and methods for reconstructing hunting weaponry in detail based on lithic data are still largely in development [see 14].

In this paper, we address the question of Upper Palaeolithic weaponry through the study of Recent and Final Gravettian lithic material from Abri Pataud, one of the key sequences for Western European Upper Palaeolithic. By applying detailed attribute recording of impact damage developed in the recent years [8, 14], we aim to 1) securely identify lithic armatures among backed tools by relying on combinations of macroscopic and microscopic use-wear features, and 2) determine whether the artefacts with convincing evidence of projectile use were hafted as weapon tips or lateral inserts on composite points.

By drawing on recent data on prey animal species and hunting strategies, we show that shifts in projectile design need to be examined in the context of information on subsistence and social organisation to better understand technological variability and change. Our study in addition contributes to an improved methodology for projectile identification by providing a critical view on the use of fracture measurements and by identifying further cases of equifinality in the formation of production and use-related wear.

## 2. Gravettian hunting weaponry in context

The Gravettian ’technocomplex’ spans over a period of nearly 9000 years (c. 29,000–20,000 BP) and an extensive geographical region [15]. Its unity/heterogeneity, internal chronology, and the interlinkage of regional technological traditions continue to be discussed [6, 15–28]. The time period is characterised by considerable climatic fluctuations and large-scale environmental change that culminates in the Last Glacial Maximum (LGM) [29, 30]. The environmental and faunal record in Western and Central Europe generally reflects steppe and tundra-like landscapes and limited tree cover although biotopes appear to have been rather varied even on a local scale [31–39]. Gravettian hunters frequently exploited large game, notably horse, reindeer, red deer, mammoth, and bison [31–34, 36, 40–43]. The site of Kraków Spadzista in Poland has yielded direct evidence of mammoth hunting at around 25,000–24,000 BP [44], and it has been argued that pressure from human hunters may have contributed to mammoth extinction at least regionally [31, 37, 44].

Importantly, small terrestrial and avian fauna, together with small ruminants (ibex and chamois), were captured alongside larger game in Gravettian contexts [33, 34, 45–47]. Varied hunting strategies, including trapping, have consequently been suggested for some sites [46, 48]. Fishing becomes archaeologically pronounced only in the Magdalenian when obvious fishing implements (e.g. harpoons and fishgigs) and numerous artistic depictions of fish enter the archaeological record, but it is likely that fishing has a much longer history in Europe [49]. Fish remains are known from Gravettian contexts [45, 50] and isotope studies signal the consumption of aquatic resources [51, 52]. In this context, it is relevant to note that the exceptional finds from different regions in Europe that testify to the manufacture of textiles, netting, basketry and cordage out of perishable materials during the Gravettian [53, 54] represent know-how that may have implications for hunting and fishing technologies. Evidence of plant foods and related technologies is also emerging [55].

Subsistence practices have often been assumed to show increasing specialisation and resource intensification from the early Upper Palaeolithic towards the Magdalenian. Yet, when in-depth faunal data are examined, factors such as seasonality and regional changes in biomass and biodiversity due to climate change can interfere with the formulation of such overarching hypotheses [56]. In our view, this dilemma calls for a careful examination of faunal and technological data side by side, beginning with individual sites with sufficiently rich and well-dated assemblages, and eventually combining these datasets into diachronic and regional comparisons.

The Gravettian lithic record is characterised by the presence of distinct artefacts with morphologies suggestive of projectile function, including tanged points, Gravette and microgravette points, and *fléchettes*. Projectile use has been confirmed for some of them through use-wear studies relying on tailored experimental reference material [14, 57, 58], but other functional analyses have demonstrated that not all pointed objects functioned as armatures [59–62].

Despite the fact that detailed use-wear data is often only marginally available and the direct archaeological evidence of spearthrowers and bows still postdates the Gravettian [63, 64], the new lithic point types that appear during this period have repeatedly been associated with the introduction of new kinds of weaponry and characterised as ’improvements’ to hunting equipment [65]. Some researchers have proposed that Gravettes and microgravettes represent lighter points adapted to higher propulsion velocities enabled by long-range weaponry (spearthrower or bow) [66]. Others have gone further in their speculations and argued, based on circumstantial evidence (metric comparisons with ethnographic arrows), that delicate microgravette points could represent arrowheads [67]. Yet, detailed data on armature hafting is largely lacking, and alternative hypotheses, such as lateral hafting of small-sized backed lithics [68, 69], cannot be ruled out. In addition, recent research efforts have shown that in the absence of organic components (preserved propulsion systems, shaft elements), the reconstruction of prehistoric weapon systems is a demanding task and methodological groundwork is still under way [14]. The above-cited claims therefore are yet to be critically evaluated.

The truncated and segmented backed bladelets in the younger Gravettian assemblages have been frequently perceived as deriving from composite points [6, 65, 70], but rigorous functional studies addressing Gravettian composite designs through experimental replication have not been conducted with the exception of a single recent study [71]. In sum, while a great deal has been assumed, little has been proved regarding the development of hunting weaponry during the Gravettian.

Against this background, we designed a case study to investigate potential changes in lithic armature hafting and weapon design across the Recent/Final Gravettian boundary at Abri Pataud. The Level 3 (c. 24,000 BP) and Level 2 (c. 22,000 BP) lithic collections are rich in supposed Gravettian armatures (Gravettes, microgravettes, backed pieces) and had not been previously studied from a use-wear point of view. We offer new insights into weapon design and propose ways to improve the existing methodology for studying it. We take the standpoint that detailed analysis of individual site contexts constitutes a necessary first step in attempts to understand links between hunting equipment and human adaptations on a broader scale.

## 3. Material

### 3.1 Site

The site of Abri Pataud is located in the village of Les Eyzies-de-Tayac-Sireuil in Dordogne, southwestern France, and is one of the key sequences for the Upper Palaeolithic in Western Europe. The rock shelter, formed by frost weathering at the base of a west-southwest-facing cliff, is situated on the left bank of the meandering Vézère that is a tributary of the Dordogne [39, 72–74]. The site was first systematically excavated by Hallam L. Movius in the 1950s and 1960s using techniques that were exemplary for their time [75]. The remains of the Upper Palaeolithic occupations at Pataud have preserved within a c. 9m thick layer of sedimentary deposits and include nine Aurignacian occupation layers (Levels 6–14), four Gravettian levels (2–5) and a Solutrean level (1) that are generally separated from each other by sterile or nearly sterile deposits (*éboulis*). Abri Pataud thus presents an exceptionally complete Upper Palaeolithic sequence widely discussed elsewhere [see e.g. 76 for the Aurignacian and 77 for the Gravettian sequence]. Site formation and postdepositional processes have also been extensively studied and published [39, 73, 78–80]. The Level 2 deposits have also yielded an important and unique assemblage of human remains [72, 81–83].

Levels 3 and 2 that are the focus here correspond to Recent and Final Gravettian occupations, respectively. These two levels have been perceived to reflect short-term occupations in comparison with the more voluminous underlying Levels 4 and 5 [84].

The Level 3 occupations have been dated to around 24,000 BP (between 30,030 and 27,350 cal BP). The estimated total duration of this occupation phase is currently over 2000 years, but the low number of determinations in the Bayesian model means that this estimate needs to be treated with caution [84]. Level 3 occupations appear to have taken place during a period of climatic amelioration with annual average temperature perhaps a couple of degrees above zero, associated with a steppe environment [39]. The faunal record witnesses a decline in forested areas and a transition to more open landscapes over the course of Level 3 occupations [33].

Movius originally distinguished six separate occupation phases within Level 3 [85, 86]. Recent lithic refitting and re-examination of the data recorded during the excavations indicate that while some subunits that Movius treated as separate stages of occupation are connected by refits and therefore call for re-examination of the chronology, the integrity of Movius’s units can for large part be maintained [77, 87–90]. A number of recent studies have grouped some of Movius’s original occupation lenses and *éboulis* into larger units (*ensembles*) to account for their sometimes blurry limits. This grouping combines the topmost occupation Lens 1 with the associated *Eboulis a* (*ensemble 1*) and treats the underlying *Eboulis b* as its own unit (*ensemble 2*) to enable a separation of the occupation stages represented by Lenses 1 and 2. It unites different, partly ambiguous Lens 2 occupations (Lens 2, 2 main, 2a, 2b) into a single unit (*ensemble 3)* and connects the two oldest Level 3 occupations (Lenses 3 and 4) with each other and the surrounding *éboulis* (*ensemble 4*) [33, 87, 91].

The Level 2 occupations date to around 22,000 BP (27,000 to 25,800 cal BP) [84]. The date on a human vertebra (GrA-37873; 18,040 ± 80 BP) falls outside this range and is too young with regard to the archaeological context and the recent radiocarbon results. This discrepancy is probably due to insufficient preservation of collagen in the vertebra [92]. The age of 22,000 BP that is supported by the other samples matches that of the Final Gravettian occupation in the nearby Laugerie-Haute Est [72, 92, 93]. The start of the Final Gravettian occupations at Pataud has been narrowed down to between 27,000 and 26,500 cal BP, which corresponds to harsh, cold, and dry stadial conditions at the onset of the Last Glacial Maximum (LGM) [84]. The stratigraphic subunit containing Level 2 reflects arid or semi-arid periglacial conditions with less vegetation cover and average temperature one or two degrees below zero [39].

Only two other sites that date to this period, namely Le Blot and Les Peyrugues, are known from the region [72, 92, 93]. Studies on population dynamics suggest a decline in human populations in the area towards the LGM [94–96], which could correspond with the rarity of Final Gravettian sites in southwestern France. However, the geographical and temporal resolution of these studies is not enough to address the regional situation as they do not separate between the time periods during which Level 3 and Level 2 deposits formed. Nevertheless, the locally scarce record of Final Gravettian human presence can indicate changes in population density and/or mobility strategies.

Palaeontological and zooarchaeological studies [33, 34, 50] have established a heavy reliance on reindeer as the basis of subsistence throughout the Upper Palaeolithic sequence of Pataud. Cho has recorded a proportion of 93.9% of reindeer among identifiable remains for Level 3 and 87.6% for Level 2 [33]. The recent excavations of Level 2 deposits have yielded a faunal collection where reindeer dominates more moderately and is represented by 75.8% [34]. The archaeological record demonstrates strong and continuous cultural traditions linked to the exploitation of reindeer that reflect considerable knowledge about the behaviour of this species during different seasons [33, 34]. Bovids (aurochs/bison), horse, ibex and chamois were exploited on the side of the reindeer [33, 34, 50], and bird and fish remains are sporadically present [50].

Solifluction has contributed to the formation of the Level 2 deposits [39, 72, 79, 80]. However, refitting of lithic material from the recent excavations and the Movius collection has demonstrated that the redistribution of the remains in this level is limited both vertically and horizontally, and most refits respect the limits of the archaeological units [93]. The stratigraphy observed during excavation is therefore not created by natural processes (pseudo-stratification) alone [72, 80]. The recent studies have thus been able to confirm the hypothesis hesitantly put forward by Movius [74] that Level 2, similarly to Level 3, represents a sequence of human occupations. While solifluction has affected both levels, the archaeological evidence shows that it was not extensive enough to completely perturbate the levels and their subunits. The levels can therefore be considered to have maintained their integrity. Study of the spatial distribution of artefacts, on the other hand, is not feasible.

### 3.2 Lithic assemblage and backed tools

Backed tools, which include Gravette points, microgravette points, and backed bladelets, are among the type fossils of Gravettian industries, and form an important component of the Level 3 and 2 assemblages [see 85]. In other contexts, these items have been classically perceived as projectile armatures although knife function has sometimes been proposed for some of them [59–61, 69] and also use in craft activities has been reported [69]. The shift from Gravette and microgravette points to truncated backed pieces has been viewed as the replacement of distally hafted weapons tips by laterally hafted ones towards the end of the Gravettian [6].

We focused here on the Movius collection, which contains these artefacts in abundance. In addition, a small number of artefacts recovered during the rescue excavation in 1989 were included. The material studied here belongs to the collections of Muséum national d’Histoire naturelle (France) and is held at the laboratory of Abri Pataud (Les Eyzies-de-Tayac-Sireuil). The artefact identification numbers (format AP[Abri Pataud]/XX[excavation year]-X[level]-XXX[level-specific ID]) used here correspond to the existing find catalogues and databases. No permits were required for the described study, which complied with all relevant regulations.

While Movius’s excavation techniques were advanced for their time [75], the recent excavations of Level 2 in 2004–2009 have demonstrated that only fine sieving can recover the smallest size groups of backed pieces. The new assemblage is much more heavily dominated by backed bladelets than the older collection, with microlithic elements representing more than 70% of the retouched tools as opposed to the much more moderate c. 40% recovered in the older excavations [97]. The Movius collection can therefore be considered somewhat biased towards larger backed artefacts, and the number of small-sized items is probably an underestimate of their true proportion. While this poses certain limits for understanding variability in weapon design, excavation techniques in the 1950s and 1960s were similar for the two levels and the backed tool collections can therefore be regarded as roughly comparable in terms of the loss of the smallest fraction of backed artefacts. This makes the present samples sufficient for the research questions at hand.

#### 3.2.1 Level 3

The Level 3 lithic assemblage comprises altogether 33,786 lithics, among which 1662 formal tools, and c. 2000 pieces of debris [88]. The flint used during the Level 3 occupations comes from several different sources. The most frequently used variety is the local Senonian flint that was available in primary and secondary deposits near the site [97]. Senonian flint makes up about 90% of the Level 3 lithic assemblage measured in weight [90], and extensive knapping activities using this variety of flint are reflected in high number of cores and preparation flakes recovered at the site [88]. The second most common raw material is Bergerac flint, represented by c. 5% in the assemblage [90]. The outcrops of this and other non-local raw materials, present in the assemblage in minor quantities, are located c. 45–60km from Abri Pataud [97].

The industry is based on blade and bladelet production from single and double platform cores without a true technical division between the two core categories, and some of the refits show a continuum of blade and bladelet production from a single core [90]. Technological and functional studies have demonstrated that blanks were also produced from burin cores [77, 88, 98]. From a typological point of view, the assemblage is characterised by an abundance of Gravette and particularly microgravette points as well as burins. Bitruncated backed bladelets are present but very few [99].

**Fig 1:**
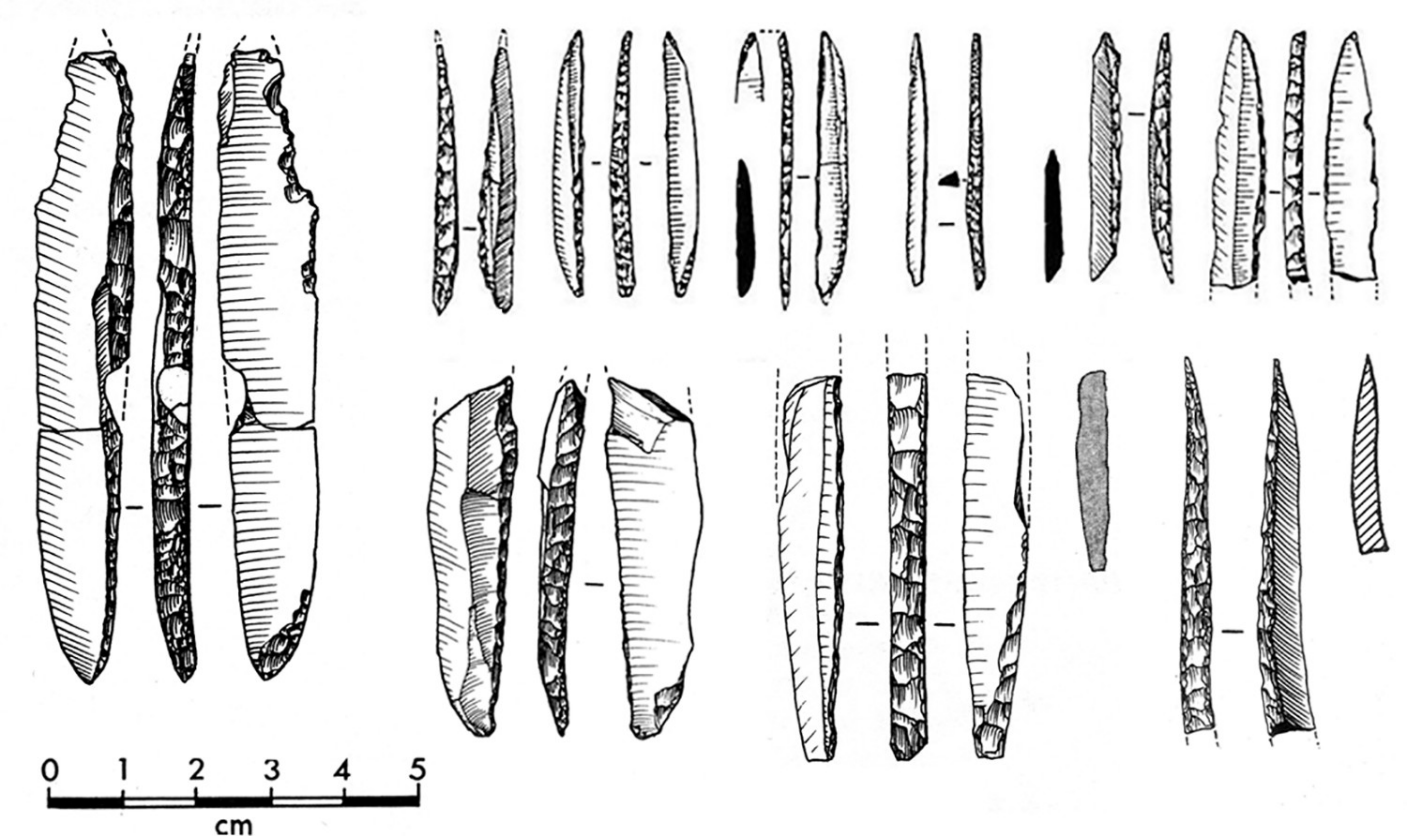
Examples of Gravette and microgravette points from Level 3. Drawings P. Laurent [85, 100].

**Fig 2:**
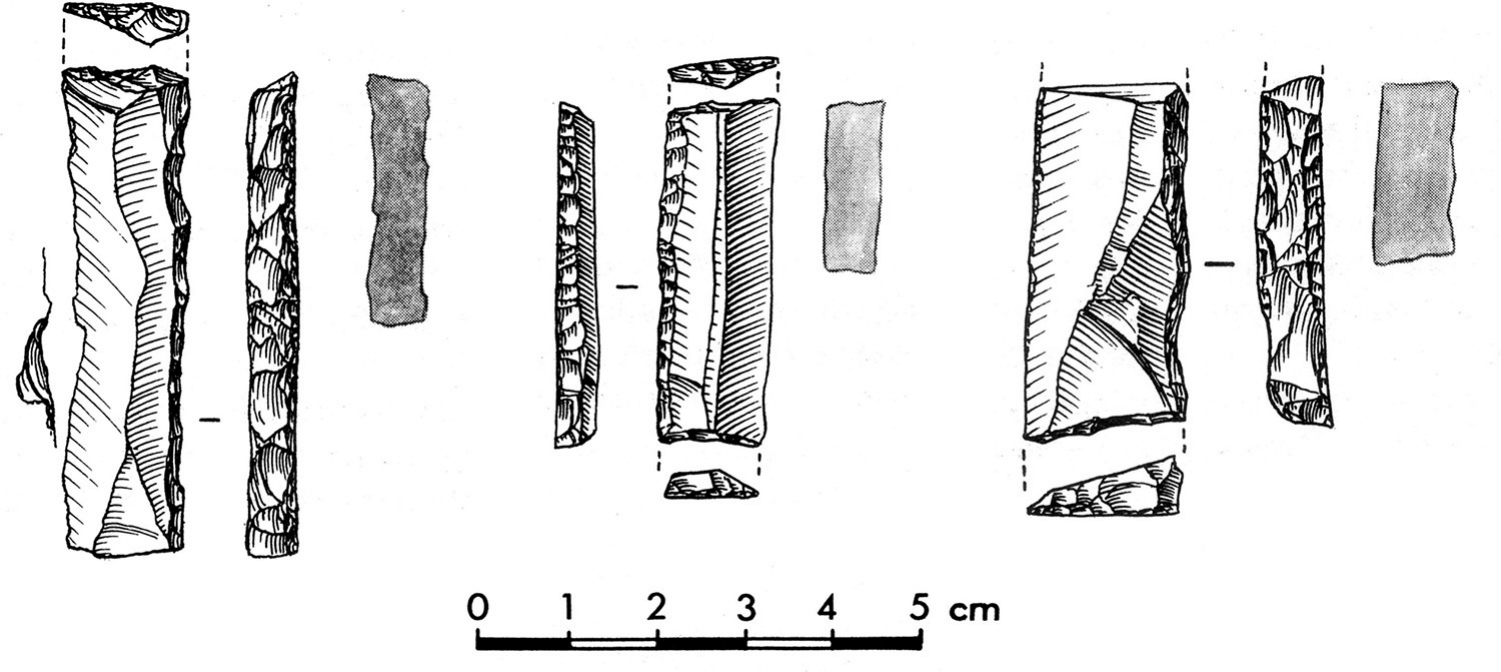
Examples of truncated backed pieces from Level 3. Drawings P. Laurent [100].

The backed tool assemblage from Level 3 consists of Gravette and microgravette points (Fig 1, Table 1) as well as rare truncated backed pieces (Fig 2). The collection has previously been subject to several typological and technological studies [87, 88, 90, 99, 100]. Earlier works have used different criteria for dividing the Gravette point family into subgroups according to size, but these groups resemble each other in terms of shaping [88, 100]. Microgravettes dominate heavily over larger points, and truncated backed bladelets are limited in number, represented by only 14 artefacts [88]. Blanks for backed artefacts were obtained from blade cores and, at least in some cases, burin cores [88–90]. Functional analysis of Level 3 burins supports the presence of burin cores at the site [98]. A recent study focused on the oldest and youngest occupations of Level 3 indicates that the backs of the artefacts were shaped at least by direct percussion using a soft stone hammer, and in some cases potentially by pressure flaking [87].

**Table 1:** Frequencies of backed tools in the Movius collection from Level 3 of Abri Pataud [88 table 53].

Artefact category	n
Gravette and microgravette point	279
Backed piece	51
**Total**	**330**

In terms of raw materials, the local Senonian flint dominates, accounting for between 54% and 59% of the artefacts in each category of backed tools (Gravette points, microgravettes, truncated backed pieces) [88]. The clearest contrast in the counts reported by Nespoulet [88] is the proportionately high amount of artefacts in Bergerac and other exotic (regional) flints among the largest points in the Gravette family, and the relatively low frequency of these raw materials amongst the other backed pieces.

#### 3.2.2 Level 2

The Level 2 assemblage from the Movius excavations comprises c. 17,000 lithics when splinters, debris and fragments smaller than 1cm are excluded. Among this material are 1674 formal tools [101]. The new excavations have yielded an additional 2459 lithics, including 369 retouched tools [97]. Nearly 90% of the raw blanks in the Movius collection are in local Senonian flint when measured in weight. Bergerac flint is represented by 7.8% [101]. The same pattern is repeated in the material from the recent excavations [97]. The percentages are thus roughly comparable to those in Level 3.

The examination of cortical pieces from the new excavations has shown that the local flint was very frequently sourced from the alluvial deposits of the Vézère, that is, in the immediate vicinity of the site, while only a fraction was acquired from primary deposits. The variety of raw materials recovered at the site has been taken as a sign of close inter-group relations as well as interregional contacts and has been viewed as lending support to the interpretation that the Gravettian groups were highly mobile [97].

The Level 2 lithic assemblage witnesses the production of blades from single platform and double platform cores [101]. Gravette and microgravette points are absent whereas backed bladelets are numerous [70, 101]. While the production sequences were oriented towards small blades and bladelets, also large blades were sought along the side of them and were at least partly transported to the site as ready-made blanks [97]. Among domestic tools, dihedral burins are prominent and are often very large in size, whereas scrapers are relatively limited in number [70]. A particular feature of the assemblage are heavily battered spheroids of flint (often former cores) that have been used as hammers [70, 101, 102].

**Fig 3:**
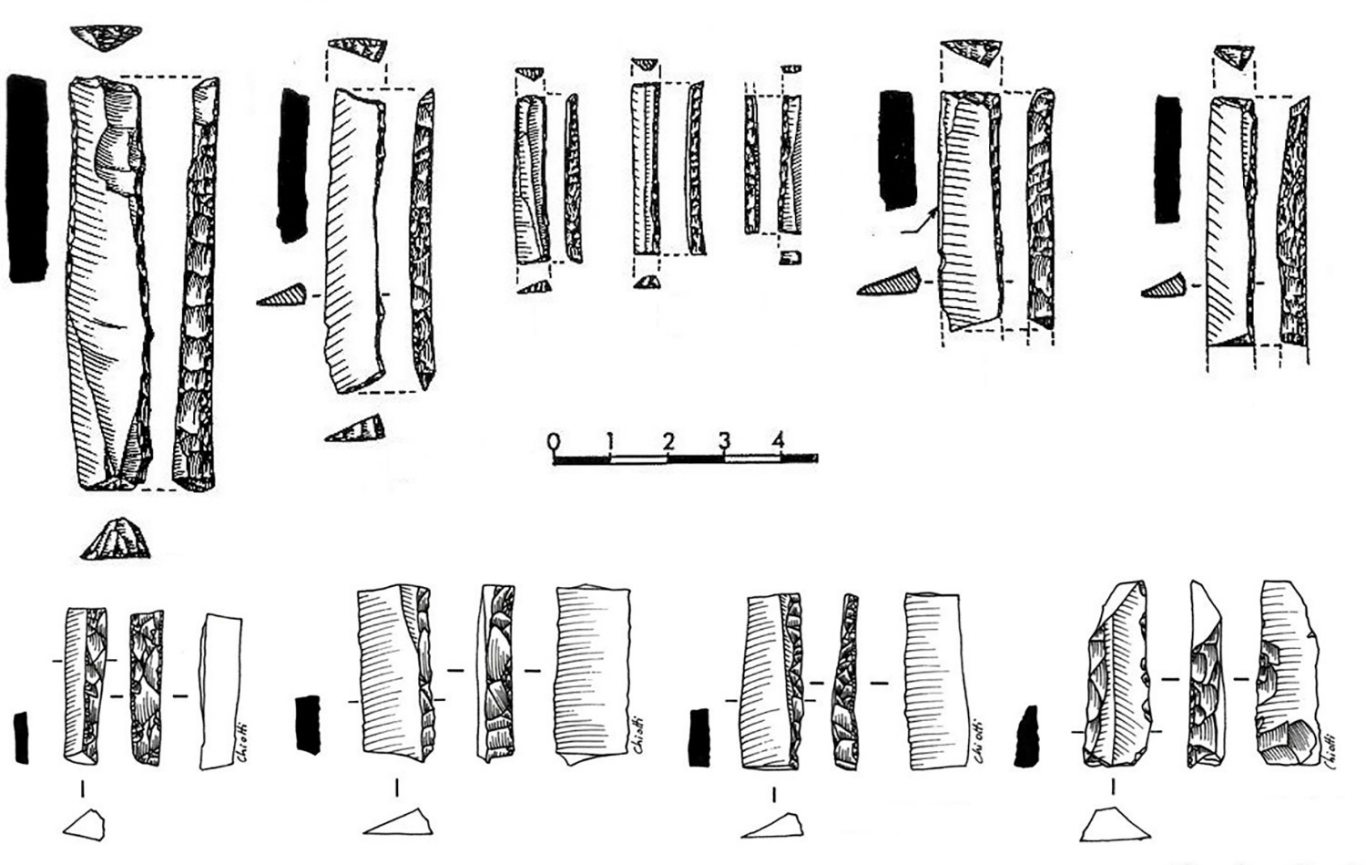
Backed pieces from Level 2. Drawings P. Laurent [85] and L. Chiotti.

Backed pieces are the most numerous artefacts in the Level 2 collection (Fig 3). Obvious Gravette points and microgravette points are absent. Even if the frequency of backed pieces in the Movius collection can be considered an underestimate of their true proportion (see above), this material provides a large enough sample for the purposes of the present study.

**Fig 4:**
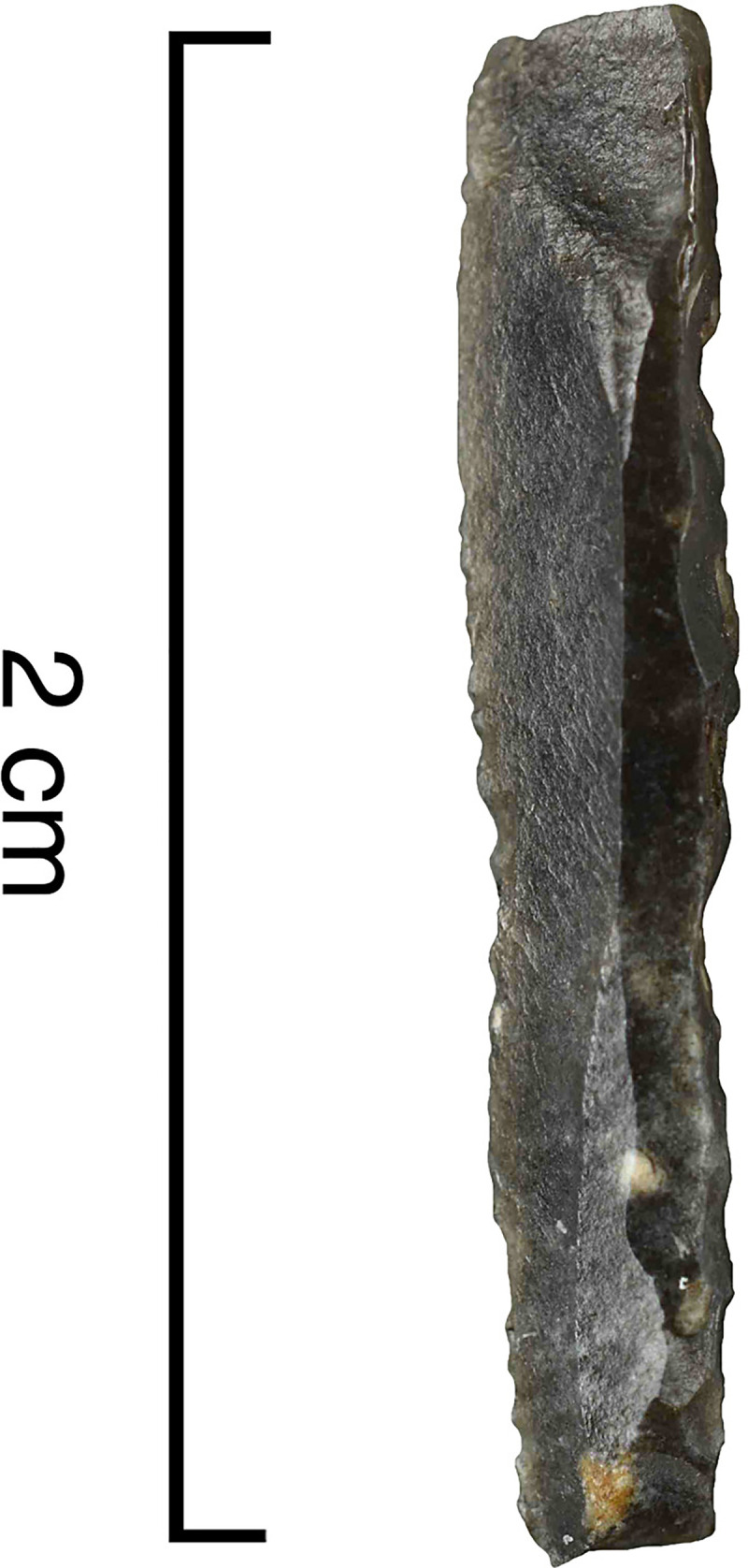
An example of a Level 2 backed bladelet with a snap break at both ends. AP/58-2-662, Senonian flint.

The backed elements were manufactured through intentional snapping [101, 103], which has produced an assemblage where backed elements with a break at both ends are the most frequent form (Fig 4), whereas pieces with one truncation are rarer and bitruncated pieces nearly absent (Table 2) [101]. According to a technological study, the length of the backed elements was deliberately controlled for by snapping and their width by shaping the back, and many of these elements (35.5% of the sample examined) show use-related scarring [101]. A link has repeatedly been proposed between the backed bladelets and composite projectile points [6, 70, 101].

**Table 2:** Classification of the Level 2 backed bladelets according to Kong-Cho [101 p. 250].

Artefact category	n
Double-backed bladelet	2
Truncated backed bladelet	126
Bitruncated backed bladelet	8
Denticulated backed bladelet	4
Other	405
**Total**	**545**

Of the total of 545 pieces, 497 derive from the Movius collection and 48 from the 1989 excavations. The category ’other’ includes backed elements without a truncation that are the most numerous objects in the subcollection.

Local flint dominates quite heavily in this category of tools. Varieties of Senonian flint are represented by 70.8%, which is a higher proportion than that recorded for domestic tools (e.g. scrapers and burins). The amount of Bergerac flint is consequently lower, at 21% as opposed to 30–40% in scrapers and burins [101]. On-site production of backed tools from local raw materials is further witnessed by the recovery of characteristic waste pieces [103].

## 4. Methods

### 4.1 Study design

The objective of the detailed use-wear analysis was to understand projectile use and weapon design during the Level 3 and Level 2 occupations at Pataud and to provide a preliminary view of possible other uses of backed tools. The primary aim was to confirm projectile use in the two samples using strict criteria relying on combinations of macroscopic and microscopic features. The subsequent goal was, with the aid of detailed recording of impact damage, to reconstruct the ways in which the armatures were hafted. Finally, we wanted to find out whether the younger part of the Pataud Gravettian record reflects long-term changes in weapon design and to identify possible factors that might explain such changes.

The reconstruction of the hafting mode of projectiles is a prerequisite of further analysis aimed at e.g. identifying weapon systems (propulsion modes) [14, 104]. The method for addressing this aspect is still in development and the experiments conducted thus far have heavily focused on distally hafted armatures (weapon tips) [71, but see 105]. The topic of propulsion modes is therefore beyond the scope of the present study. Nevertheless, the data presented here provides the necessary basis for further research questions and hypotheses as well as future experiments focused on these particular armature morphologies.

The functional study followed a step-wise approach where the entire backed tool assemblage was first screened to narrow down research questions and sampling for detailed use-wear analysis. The initial screening provided a first view of the presence/absence and relative frequency of impact damage in the material and the evaluation of possible other sources of damage (production, other tool uses, taphonomic processes). The collection was subsequently sampled for more detailed analysis focusing on artefacts that showed the highest potential for projectile use. The damage on these artefacts was recorded in detail and a smaller sample was selected for analysis under high magnification to detect MLITs (microscopic linear impact traces) and other microwear. The study was complemented with a production experiment where backed bladelets were intentionally snapped into shorter segments to investigate the characteristics of breaks produced by this process and their possible overlap with damage from projectile use.

### 4.2 Terminology

In macrowear analysis, we distinguish between ’breaks’ that are defined as fractures that cut across the armature, reaching from one edge to another and one surface to another, and ’scars’, which are more limited removals that leave at least part of the opposite edge intact [cf. 8]. Terms ’scar’ and ’removal’ are used synonymously. ’Fracture’ is used here as a general term to refer to both breaks and scars, i.e. any event of brittle breakage of lithic material under mechanical stress. The terminology for break and scar attributes (initiations, terminations) follows that used in previous published literature [e.g. 8, 106, 107]. ’Secondary scars’ (or ’secondary damage’ or ’secondary removals’) are scars that initiate on a break surface and terminate on the adjacent surface or edge. ’Lateral scarring’ (or damage) refers to removals located on the lateral edge (typically cutting edge) of the armature. By ’microscopic linear impact traces’ or ’MLITs’ [11, 107, 108] we mean distinct linear features (striations, linear polish) that clearly initiate at the termination of a macroscopic impact damage feature (a break, a lateral scar, or a secondary removal) and are probably caused by the flint particles themselves (see below).

The term ’laterally hafted’ refers here to armatures where the back is attached to the point or weapon shaft over its entire length. In contrast, part of the back of ’distally hafted’ armatures is unsupported by the shaft and protrudes from it to form a lithic tip. This category includes all lithic tips regardless of the alignment of the armature’s cutting edge with the shaft (axial vs oblique; see below).

### 4.3 Screening and preliminary analysis

The entire backed tool collection was first screened with the help of a stereomicroscope and selective use of a metallographic microscope, and basic data were recorded to aid the formulation of research questions and sampling of the material for further analysis. The artefacts were classified by morphological group and preliminary functional category. The recording differed somewhat between the analysed assemblages: for Level 3, a piece-to-piece recording was done for a large background sample (n = 329) to investigate potential causes of fracturing on a preliminary level, whereas for Level 2, the screened material (n = 693) was classified into preliminary functional categories without recording observations for individual artefacts.

The Level 3 artefacts were initially classified according to two criteria, typo-technological group (on the most basic level, different size groups of Gravette and microgravette points versus truncated backed bladelets) and break characteristics. The fragmentary nature of most pieces and the variability in overall design made straightforward grouping by techno-morphological criteria alone quite difficult. Measurement data on a sample (n = 113) were used to create three subcategories (size groups) to aid further comparisons. Each break in the background sample (n = 329) was briefly examined and classified as a potential production, impact, or taphonomic break, with non-characteristic breaks such as snaps marked as ’indeterminate’, to gain a first view of the processes responsible for the assemblage consisting mostly of fragmentary artefacts.

In contrast, the Level 2 collection that is largely made up of medial fragments, many of which could be expected to represent intentionally snapped pieces [101, 103], was classified according to the qualities of edge damage as well as breaks to distinguish between used and unused objects and to evaluate the proportion of armatures in the collection. The following categories were used: ’breaks suggestive of impact’, ’substantial lateral edge damage’, ’minor edge damage’, ’no edge damage / isolated minuscule scars’, ’production waste’, ’indeterminate’. The classification criteria are provided in S1 Appendix. In addition, a small number of pieces with minor edge damage were examined under high magnification already at this stage to identify traces of potential knife use.

### 4.4 Detailed functional analysis

The detailed use-wear analysis built on the results of the initial screening and aimed at confirming projectile use, documenting impact damage in detail, and distinguishing between distally and laterally hafted armatures using the recorded data. In addition, potential non-projectile use was evaluated on a preliminary basis drawing on the present samples.

#### 4.4.1 Sample selection

The Movius collection was sampled following the results of the initial functional screening. A small number of artefacts deriving from the 1989 rescue excavation were also included. Due to the research questions that were oriented towards understanding long-term technological changes, Levels 3 and 2 were treated during sampling as undivided units and contrasted with each other without paying attention to subdivisions (within-level occupation phases). However, after the completion of the functional study, the vertical position of some of the Level 3 artefacts was examined to better understand the observed temporal changes in armature design.

Of the screened Level 3 material, a sample of 104 pieces was selected to investigate the characteristics of the breaks and edge damage in more detail. This sample was chosen by including the main artefact categories and size groups, and by favouring pieces with abundant macroscopic wear. The sample therefore dominantly contains finished objects, whereas production discards were examined on the side (mostly without recording the damage on them in detail) to gain insights into production-related damage on the small-sized implements. The surface preservation of the Level 3 backed tools is in many cases rather poor, but a sample of tools was nevertheless subjected to high magnification analysis since it was judged crucial to be able to confirm projectile use by detecting combinations of low and high magnification features. High magnification analysis was also the only way to reliably address the question of possible other uses in the studied sample. Sixty-six pieces became analysed with high magnification. The samples are summarised in Table 3.

**Table 3:** The composition of the Level 3 backed tool low and high magnification samples.

	Low magnification	Low and high magnification
Gravette	9	5
Microgravette	43	22
Nanogravette	23	10
Bitruncated backed piece	3	4
Truncated backed piece	6	7
Other	4	3
Indet	16	15
**Total**	**104**	**66**

A total of 48 pieces were included both in the low and high magnification detailed analysis sample. The other 18 pieces that were analysed with high magnification but for which damage was not recorded in detail represent tools that were either examined for potential knife use or on which the damage was, in the absence of MLITs and other microwear, considered too ambiguous to be worth a detailed recording. The local Senonian flint was overrepresented here by 90.4% in the low magnification sample and 78.8% in the high magnification sample compared to the proportions reported by Nespoulet for backed tools [88] because the sampling strategy put a heavy focus on functional observations and surface preservation at the expense of raw material representativeness.

The initial Level 2 low magnification sample (n = 105) for which basic recording (break and edge damage category, main technological features) was done was selected by focusing on pieces interpreted as finished and used implements. In addition, similarly to what was done for the Level 3 assemblage, clear production waste pieces were analysed on the side without detailed recording to get an idea of the breaks and related features that can form during the manufacturing process (mainly backing and snapping). Besides material from the Movius collection, the sample also includes a low number of pieces (n = 8) deriving from the 1989 excavations.

In addition to the backed pieces, a single non-backed denticulated bladelet fragment was included to investigate how these edges were used, bringing the total of denticulated pieces in the sample to two out of the total of four pieces reported by Kong-Cho [101].

The structure of the samples is shown in Table 4.

**Table 4:** The composition of the Level 2 backed piece low and high magnification samples.

	Low magnification	Low and high magnification
Bitruncated backed piece	2	4
Truncated backed piece	13	9
Backed piece with a break at both ends	43	20
Backed piece with additional retouch	4	2
Truncated backed piece with additional retouch	1	1
Partially backed piece	1	2
Denticulated backed piece	2	2
Denticulated piece	0	1
**Total**	**66**	**41**

The raw material proportions in the analysed samples roughly corresponded with the ones reported in a previous technological study [101]. The bias here was in the same direction as in the Level 3 sample, with local flint varieties being slightly overrepresented, but with less of a difference (c. 80% in the present study and c. 70% in Kong-Cho’s data).

#### 4.4.2 Data collection

The detailed low magnification analysis involved recording the attributes for each relevant macroscopic fracture individually following the approach developed at TraceoLab [8, 14]. It builds on the attribute system created in the 1970s for the description of use-related fracturing on brittle solids [106]. The system used in the present study was somewhat simplified from that used by Coppe [8, 14]. Break and scar attributes (S2 Appendix) were recorded on Excel sheets so that each feature formed a single row and the wear pattern on each artefact typically became described by several rows of data. For artefacts with evidence suggestive of impact, all breaks were recorded despite the fact that particularly in the case of Level 2, many of them are probably production-related. While this resulted in the recording of features irrelevant for use interpretations, it was considered a safer approach than attempting to filter out production breaks during analysis given that at the moment no simple criteria exist for doing this (see below).

Simultaneously to the attribute recording, basic morphological and technological data (see S2 Appendix) were registered for each artefact on a separate Excel sheet to allow judging the effect of size, morphology, and location of retouch on fracture formation afterwards when relevant. The high magnification observations of functional wear were written down as verbal descriptions, and a cross-link was made to associated low magnification features. The severity of post-depositional alterations (scarring, rounding, patina, polish, bright spots, striations, polish erosion) was noted on a scale from 0 to 3 for each category individually, and presence of recent markings or additives such as metal traces, pencil marks, and refitting glue was also noted to aid the critical evaluation of high magnification use-wear observations.

#### 4.4.3 Analytical equipment and protocols

The main part of the use-wear analysis was done in the laboratory of Abri Pataud. The analysed artefacts had been previously washed, and basic cleaning using cotton and ethanol and/or acetone was sufficient to remove adhering contaminants (e.g. handling grease). A Nikon SMZ-1 stereomicroscope (magnifications between 6.6× and 40×) was used for low magnification analysis and a Nikon Optiphot metallographic microscope (magnifications between 50× and 400×) for high magnification observation. The photographic documentation of the traces was done with a Nikon D7200 digital camera mounted on the Optiphot with an adapter (NDPL-1(2X)). The images were captured with the software Nikon Camera Control Pro and z-stacked in Helicon Focus Lite. Most of the low magnification photos were taken directly with the Nikon D7200 and a macro lens (Nikon AF-S Micro NIKKOR 40mm) and stacked in Helicon Focus. The experimental material is hosted at TraceoLab (University of Liège) and was analysed using the microscopes available there, including a Zeiss stereomicroscope Discovery V12 (magnifications between 8× and 100×, oblique lighting) and a Zeiss reflected light microscope AxioImager (magnifications between 50× and 1000×). The photographs were taken under high magnification using Zeiss AxioVision software for both image capture and z-stacking. Low magnification images were taken with a Zeiss Macro-Zoom Macroscope V16 using oblique lighting. The AxioVision software was employed for image capture and the images were stacked in Helicon Focus Pro.

#### 4.4.4 Projectile identification criteria

Use-wear on lithic projectiles is extremely variable in comparison to wear that forms as a result of craft activities [11]. Because of this, projectile identification requires a particularly critical view of the variability and patterning of wear traces and an awareness of equifinality in wear formation. The most popular approach in projectile studies in the last few decades has been one that emphasises so-called diagnostic impact fractures (DIFs). These features have been experimentally demonstrated to be frequent on armatures and to occur only rarely as the result of other processes. They include bending-initiated breaks, particularly ones with step terminations and secondary scarring [107].

While Fischer et al. [107] themselves made a critical comment on some of these fractures occurring as the result of e.g. tool production, the overlap between knapping and impact breaks has rarely been addressed explicitly and has often been overlooked [see, however, 11]. A solution Fischer and colleagues proposed for distinguishing between production breaks and impact breaks was fracture chronology: only breaks that demonstrably occurred after the shaping of the artefact can be considered impact-related [107] This control measure has sometimes been adopted in the analysis of archaeological backed artefacts [e.g. 109, 110]. The application of this criterion, however, requires detailed understanding of potential production-related breaks that may in some cases occur accidentally during the shaping of the back or intentionally after it (see below).

Due to these complications in using the DIF approach, as well as other criticisms it has drawn [8, 11], we opted here to use combinations of features instead of single breaks to identify armatures. This analytical approach is informed by the ongoing methodological work on projectiles carried out at TraceoLab [e.g. 8, 14], which involves refining criteria for projectile identification.

Ideally, an artefact should show a set of macroscopic features suggestive of impact as well as so-called MLITs (microscopic linear impact traces) that are clearly associated with the macroscopic damage as well as aligned with the direction of impact [see 11, 107, 108]. Some researchers judge that MLITs may also form through direct contact with bone [111]. In this study, however, we focused exclusively on linear features that occur in direct association with impact damage (breaks, secondary damage, or lateral scarring) and that therefore have most probably been caused by detached flint fragments.

**Fig 5:**
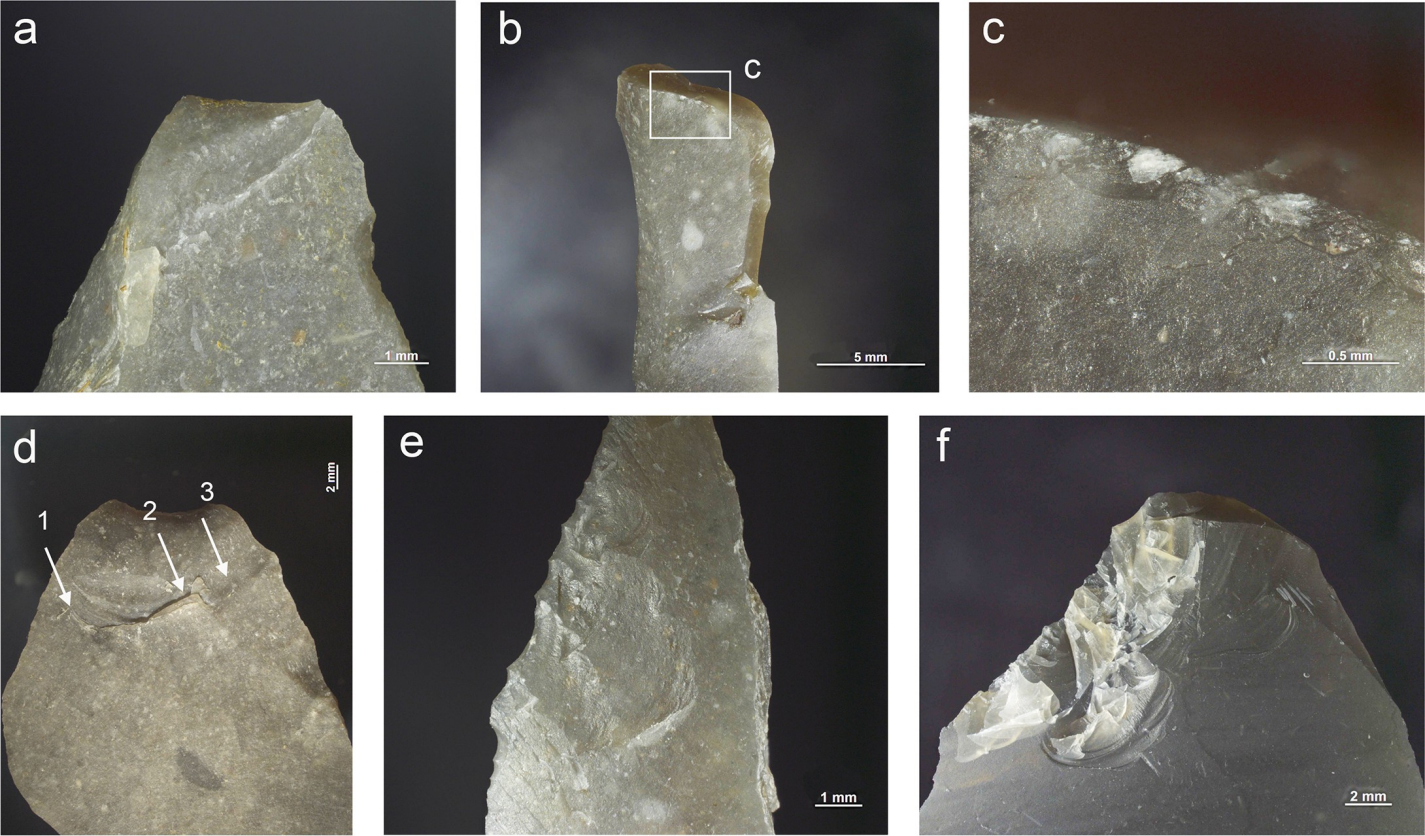
Impact damage on experimental armatures. a. A ventrally initiated bending break with a fissured step termination on microgravette point Exp. 46/59 (25×); b. A bending break with long propagation initiated on the backed edge and terminated on the opposite edge, associated with dorsal secondary damage, on microgravette Exp. 46/342 (10×); c. Secondary damage on the break shown in b., partly showing crushed initiations (90×); d. A dorsally initiated bending break with a complex termination combining step (1), hinge with fissure (2), and feather (3) on tanged point Exp. 78/24 (photo J. Coppe); e. Invasive obliquely oriented lateral scarring, some with fissured terminations, on microgravette Exp. 46/217 (20×); f. A small tip break initiated on the lateral edge and heavy obliquely oriented edge damage with crushed initiations and fissured terminations on tanged point Exp. 78/10 (10×).

**Fig 6:**
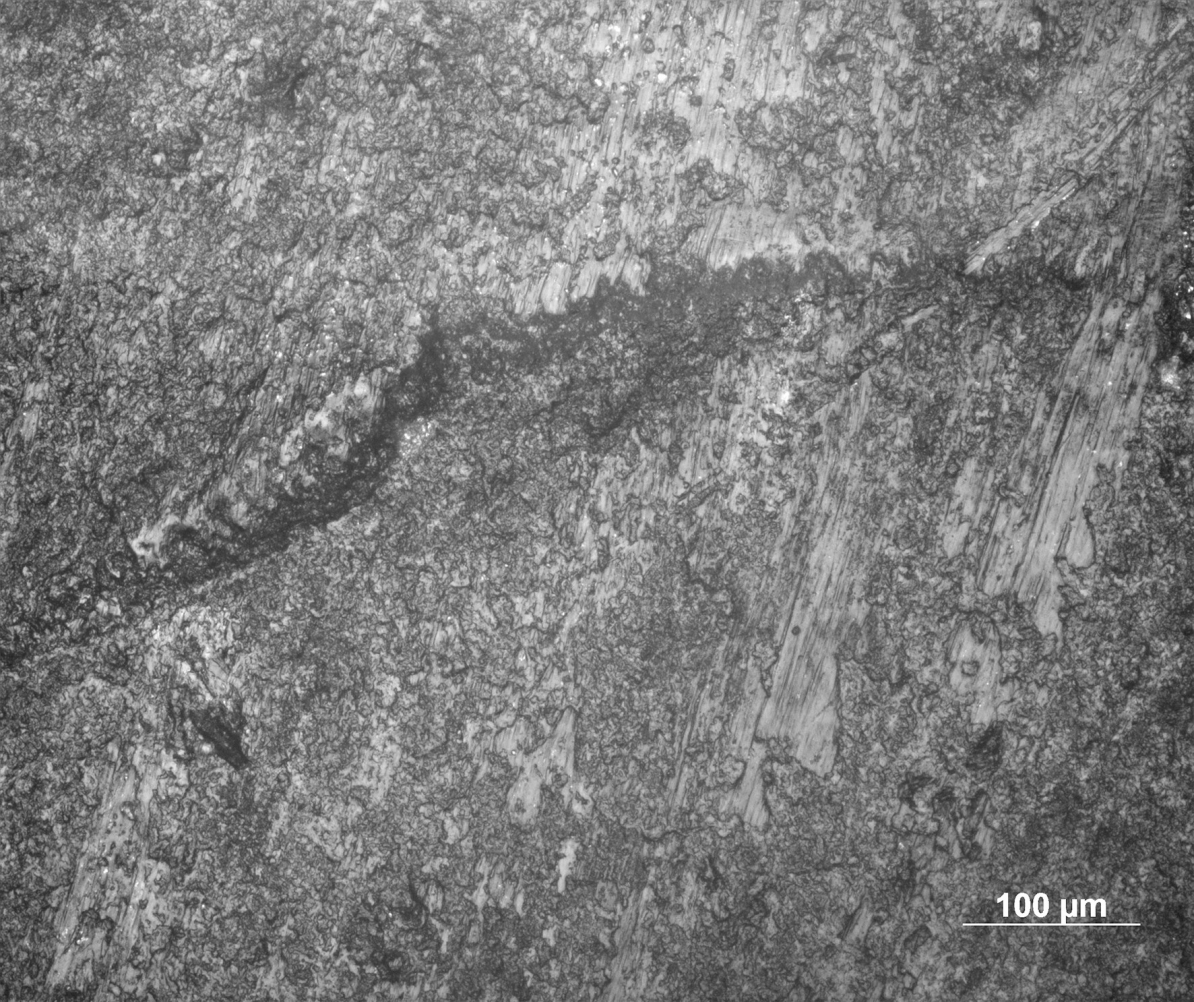
MLITs (microscopic linear impact traces) associated with tip break on a microlithic experimental armature Exp. 3/26 (200×).

While MLITs are a useful clue to projectile function, they are not present on all damaged armatures [105], which means that projectile identification has to rely partly on sets of macroscopic features. In these cases, an artefact should show several zones of fractures [breaks or invasive scarring; see 8, 11] that are logically patterned and aligned with respect to each other and the direction of impact. Among features that are particularly informative in this respect are those that testify to strong compressive forces at the moment of breakage, including fissured terminations, removed (crushed) initiations, and long fracture propagations [14]. Examples of typical macroscopic features on experimental projectiles are shown in Fig 5 and an example of MLITs in Fig 6.

Taphonomic breakage adds to the factors that can complicate the identification of projectiles archaeologically. Particularly small-sized implements break easily as the result of, for instance, trampling. Chesnaux’s trampling experiment involving backed microliths produced clean snap breaks but also breaks with slightly longer propagations (<1.5mm) as well as associated secondary removals, some of which measured just above 1mm in length [111]. O’Farrell has reported impact-like breaks up to 3mm in length on trampled experimental Gravette points [112]. When a projectile breaks on impact, a relatively dominant compressive component (acting in the direction of the length of the armature) can generally be expected, but since several factors, including the properties of the target and the angle of impact, affect fracture formation [14], overlap between trace patterns from different origins can be expected.

Lateral damage oriented obliquely or longitudinally to the edge of the tool is the only category of impact wear that is not frequently reported from production and trampling experiments. It is particularly important for the identification of lateral inserts (composite point elements) because these show characteristic breaks more rarely than armature tips [e.g. 105, 111]. While oriented scarring on the cutting edges of armatures would deserve a more central role in the sets of criteria used for identifying projectiles than it has had thus far [8], it is clear that lateral scarring can also result from other forms of tool use, e.g. butchering [11, 113]. Attributes of lateral scars suggestive of strong compression and therefore potential projectile use include the occurrence of multiple scars with consistent (unidirectional) orientations, crushed or removed initiations (Fig 5F), long propagations, and fissured terminations (Fig 5E) [14].

Here, an artefact needed to minimally show a combination of a break and other features (MLITs and/or oriented lateral scarring and/or secondary damage located on the surface of break initiation) to be confidently identified as a projectile. When a piece only showed lateral scarring, at least two of the attributes mentioned above or MLITs in direct association with the scarring had to be present to warrant a secure projectile identification.

While we consider our identification criteria conservative enough to ensure reliable functional inferences, the method has to be formalised and tested with additional experimental datasets. This work is currently in progress [8, 14], and the approach used here can be considered an application of the new method in its preliminary stage. At the core of this approach is the detailed recording of fracture frequencies and attributes (see above), which has the benefit of forcing the analyst to employ systematic and detailed recording of use-wear attributes instead of the use of somewhat opaque categories of ‘diagnostic’ features [8]. This higher rigour in data collection, although time-consuming, allows producing datasets that are directly comparable between collections and can therefore contribute to work that aims at addressing higher-level questions linked to projectile hafting and use.

#### 4.4.5 Experimental projectile reference collection

The projectile reference collection available at TraceoLab currently includes c. 1000 experimental artefacts [114]. Among these are Gravette and microgravette points hafted axially and obliquely and shot into an artificial target using different propulsion modes [8, 14]. While the flint raw material (Harmignies, Belgium) and the artefact morphology are not exact matches to the Pataud material, the reference collection provides a suitable starting point for interpreting the Level 3 collection. Laterally hafted armatures, on the other hand, are currently less well represented in the reference material [but see 71, 105, 115], and at the moment morphologies directly comparable to the Pataud artefacts are not available. As artefact morphometrics significantly affect impact fracture patterns [11, 14, 107, 116], this is an important limitation of the present reference collection, and further experimentation is needed to properly address the hypotheses formulated in this study. There was, however, enough reference material available to us to gain a basic understanding of damage patterns on laterally hafted armatures [e.g. 105], and this material, together with other published results [111, 116–118] helped interpret the Pataud Level 2 collection. To address the particular production breaks expected in the same subassemblage and to examine their overlap with impact damage with the goal of attaining more reliable projectile identifications, we designed a small-scale production experiment that complemented the reference material (see below).

### 4.5 Experimental program

#### 4.5.1 Rationale

The Pataud material, and particularly the Level 2 collection, poses a particular challenge for projectile identification because it is composed of backed elements that have been reduced to their desired length by deliberate snapping [101, 103]. While it was initially expected that intentional fracturing by bending would mostly lead to clean snap breaks that are not easily mistaken for impact fractures, which should generally involve a rather pronounced compressive component [14], no experimental material was available that would have allowed confirming this hypothesis. An experiment was therefore designed to address this question, and involved intentional breaking of backed bladelets by bending. The results of this study helped refine the criteria for projectile identification in the particular case of the Pataud Level 2 sample.

#### 4.5.2 Experimental setup

**Fig 7:**
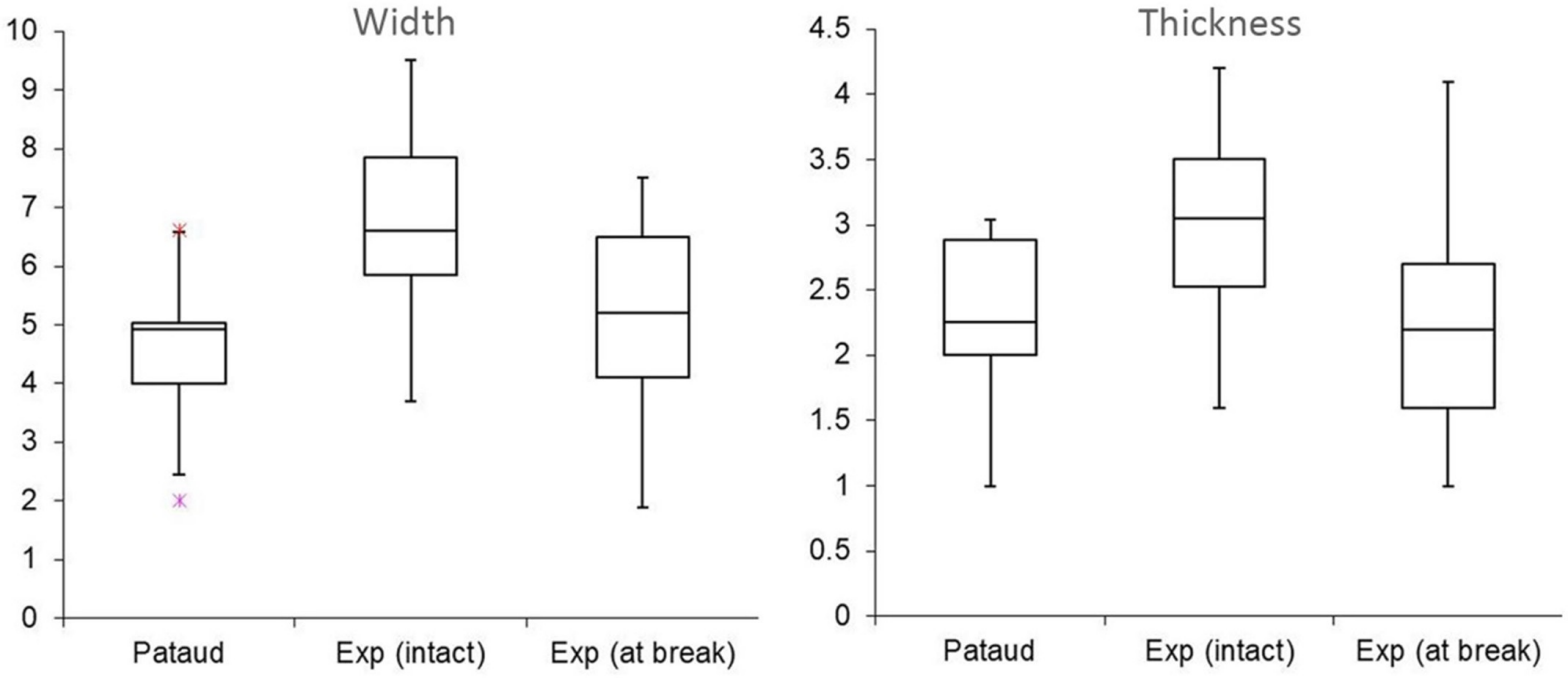
Width and thickness measurements for the experimental intentionally snapped bladelets, shown next to an archaeological sample (n = 24).

To better understand the effect of intentional breakage on backed tool assemblages, twenty-six experimental backed bladelets were produced and snapped into smaller fragments. The flint used in the experiment was Harmignies flint from Belgium. Justin Coppe retouched previously knapped bladelets by direct retouch using a sandstone billet and a wooden anvil. To reproduce the dimensions observed in the archaeological material, a sample of artefacts with secondary damage initiating from a snap break were measured from both samples of projectiles (n = 9 for Level 3 and n = 15 for Level 2). One of us (NT) subsequently snapped the artefacts either by hand, or, in cases where the bladelet proved too resistant, by pressing one end of the bladelet against a hard surface (table with coating that makes the surface of it slightly yielding). This choice (as opposed to snapping using hammer and anvil) was made because of the clear dominance of bending breaks in the archaeological material. Due to the frequent curvature of the blanks, most bladelets were easiest to snap by bending the ventral surface outwards. The experimental sample therefore shows a heavy bias towards ventrally initiated breaks. When measured at the break, the widths and thicknesses of the experimental and archaeological pieces correspond to each other sufficiently to allow initial comparisons (Fig 7). The measurements taken on intact pieces (n = 26) clearly differ from those measured at the break (n = 45), which can be explained by the fact that the experimental artefacts do not represent finished objects and in many cases retained the original width and thickness of the blank at one extremity.

The somewhat bigger size of the experimental specimens should in general be expected to lead to the formation of larger fractures since more energy is required to break them [see 14]. This means that the features produced can be exaggerated in dimensions when compared to the archaeological sample. The fact that most breaks are ventrally initiated adds to this because guiding dorsal ridges will cause the fracture front to propagate in the direction of the ridge instead of spreading laterally [119], which typically leads into longer, narrower terminations on dorsal surfaces (ridges) as opposed to flat ventral surfaces.

The breaks produced by intentional snapping were observed under low magnification (stereomicroscope) and the following attributes recorded: feature type (break/secondary scar), location, initiation (location and type), termination (location and type), propagation length with and without fissures, and association with other features.

## 5. Results

### 5.1 Experimental results

**Fig 8:**
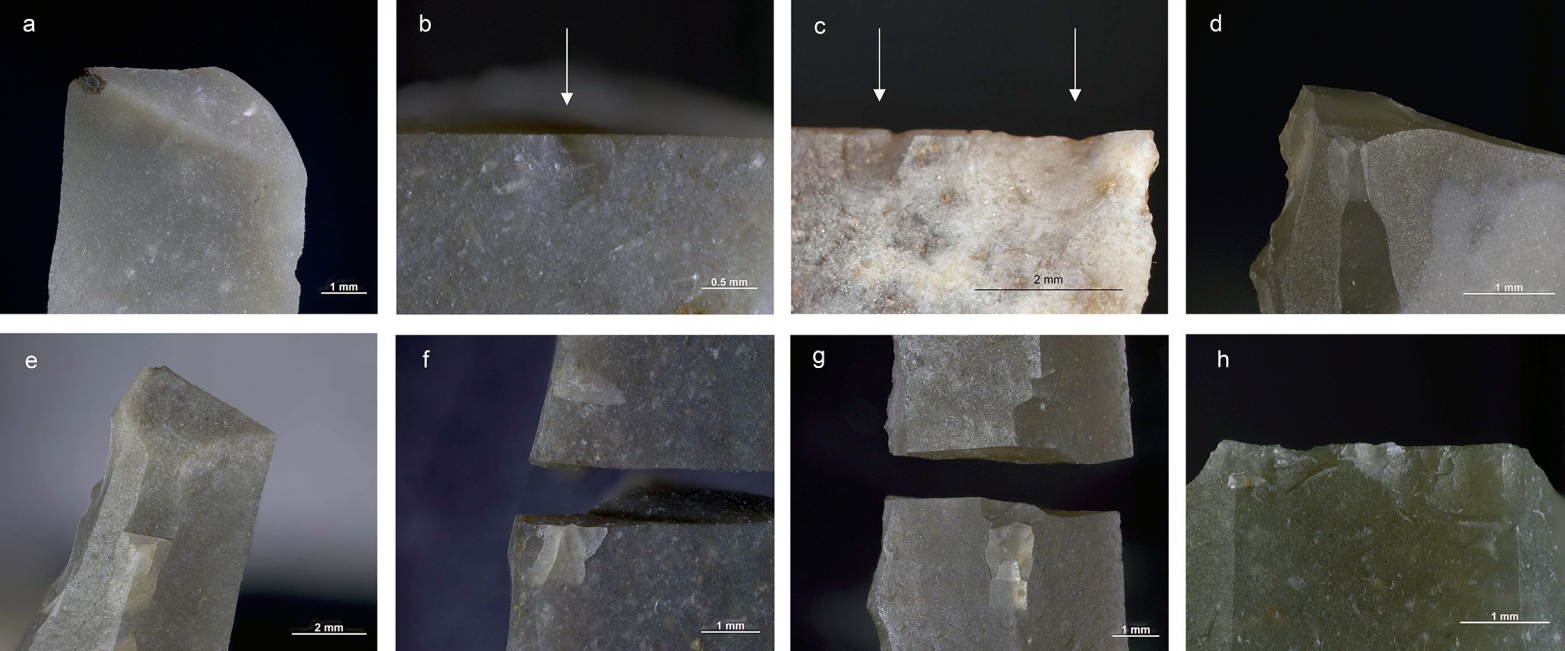
Production-related damage on experimental and archaeological backed bladelets. a. An oblique snap break on Exp. 48/113, broken accidentally during backing (25×); b. A secondary scar on accidentally snapped Exp. 48/93 (63×); c. Two similar secondary scars on archaeological piece AP/59-3-1771, interpreted as a production discard; d. A fissured step termination on intentionally snapped Exp. 48/107 (50×); e. A bending-initiated step/fissure-terminated break with a long propagation on Exp. 48/100, produced by intentional snapping (20×); f. A step/fissure-terminated secondary removal opposite to incipient removals on the intentionally snapped Exp. 48/92 (32×); g. Secondary removals on dorsal ridge on the intentionally snapped Exp. 48/95 (25×); h. Continuous secondary scarring with partly removed initiations on the dorsal aspect of the intentionally snapped backed bladelet Exp. 48/90 (50×).

Altogether 42 intentional breaks were created during the experiment. In addition, three bladelets broke accidentally during backing. The last mentioned display dorsally bending-initiated snaps, two of them with an oblique direction against the long axis of the piece (Fig 8). These kinds of breaks are frequent in the archaeological collection. When secondary removals occur in association with the accidental breaks on the experimental specimens, they are always located on the surface of the termination of the break. Secondary scars similar to those observed experimentally were recorded on probable production discards in the Level 3 collection (Fig 8C), likewise on the surface on which the break terminates. One of the accidentally snapped pieces also displays an elongated removal originating from the back and following the ridge between the break surface and the ventral surface. This scar is similar to those documented on artefacts in the Level 2 collection (S3 Appendix) and suggests that at least some of them can be linked to production.

Of the intentional breaks (n = 42), 26 were achieved by snapping by hand, and the remaining 16 by bending the artefact against the table. Like the accidental breaks, these breaks are all bending-initiated. Ventral initiations (n = 36) dominate heavily over dorsal ones (n = 6). As expected, true snap breaks are frequent (n = 19), but also other terminations occur, and fissuration is common (Table 5; Fig 8). This is probably due to the tendency of the fractures to propagate along a dorsal ridge despite the relatively low amount of energy required to break these thin and narrow objects and the fact that some compression is involved in breaking bladelets by hand. This is illustrated in the presence of step/fissure-terminating breaks with a long propagation (in proportion to the overall dimensions of the artefact), some of which would clearly classify as so-called diagnostic impact fractures (DIFs) [see 107] (Fig 8E). Other researchers have obtained similar results [112].

**Table 5:** Terminations recorded on the intentionally snapped experimental bladelets (Exp. 48/88–113).

Termination category	n
Snap	19
Feather	12
Step	5
Hinge	2
Complex	4
Fissured	10
**Total**	**42**

’Complex’ terminations combine different termination types (e.g. step-to-step, step and hinge) [see 8]. The fissured terminations are part of other terminations (e.g. step, complex) and are therefore double-counted.

Secondary damage is frequent on the intentional breaks. Of the 42 breaks, 18 show some form of it. This includes patches of two or more fully formed scars (n = 14), singly occurring scars (n = 6), and incipient or partly detached secondary removals (n = 2). Cone initiations dominate; occurrences where the initiations of secondary removals are completely destroyed are extremely rare. With a single exception (Exp. 48/98), all the secondary scars are located on the surface where the break terminates. The sole secondary scar located on the surface of initiation was found on a dorsal ridge and is microscopic (c. 0.1mm in length) and ill-defined. This is as expected since the surface of termination is where compression occurs when a bladelet breaks by bending [14, 107]. In principle, breaking against hard surface in our experiment is a process similar to the trampling situation described by Chesnaux [111] and bears similar consequences for projectile identification.

Based on their experimental work, Fischer et al. have proposed that besides their location, also the relative size of secondary scars (“spin-offs”) could be used in determining whether or not they are the result of projectile use. These authors tentatively suggested a minimum length of 1mm for small armatures such as transverse arrowheads [107]. Other researchers have also observed that the size of impact features is proportionate to armature size [e.g. 116, 120]. We therefore examined whether the size of production-related pseudo-impact fractures showed clear patterning.

**Fig 9:**
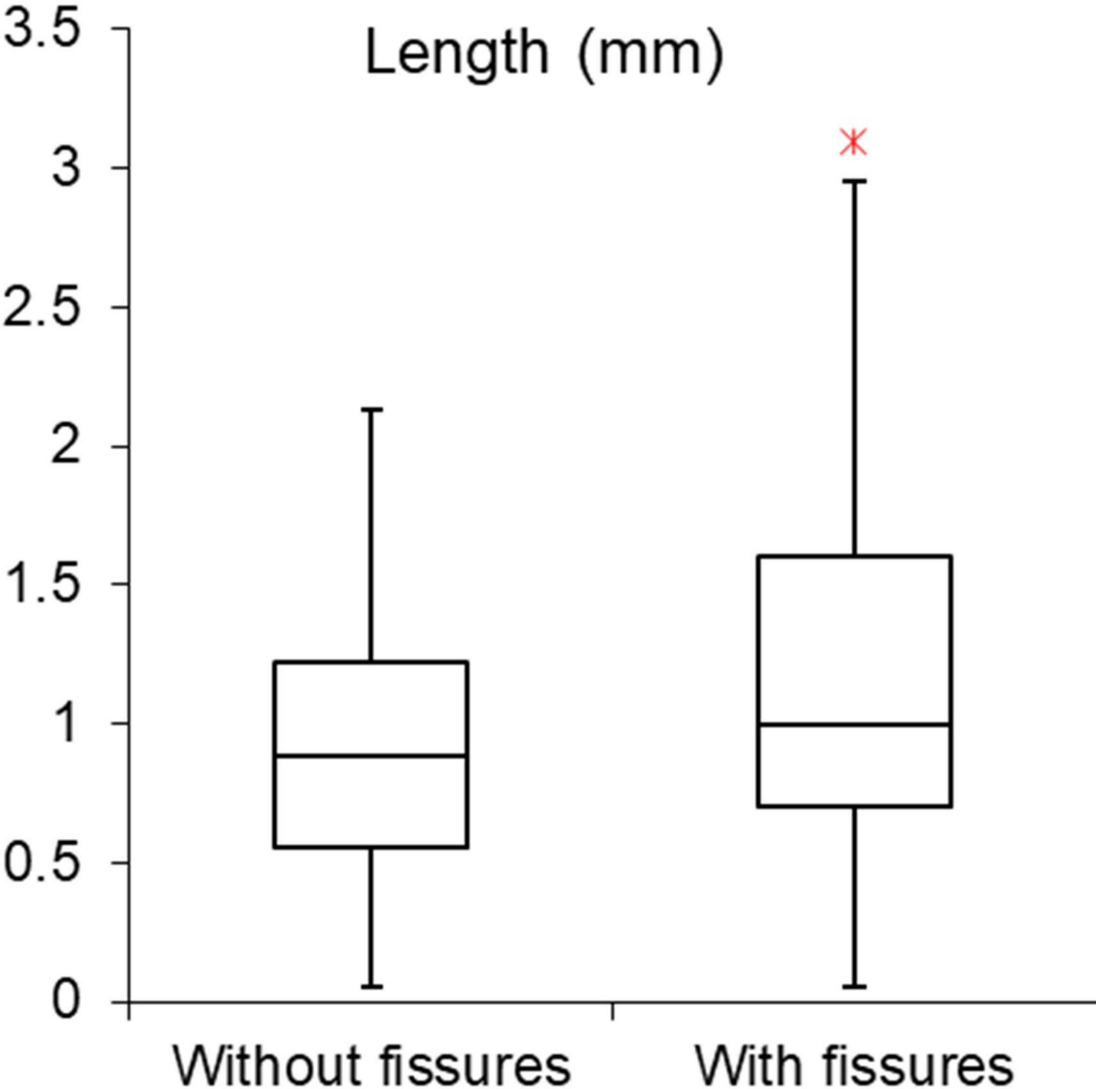
Length (in mm) of secondary scars associated with bending breaks in the experimental sample of intentionally snapped bladelets (n = 20) with and without fissures associated with the terminations.

While the production-related secondary scars are not excessively long, their median length is close to the 1mm limit proposed previously [107], and the largest ones exceed 2mm in length. Fig 9 shows the length of the removals both with and without the fissures that are part of the terminations. The measurements show that significant variability can be expected in the length of secondary removals located on the dorsal ridges of backed bladelets broken by processes other than projectile use.

The results of the snapping experiment lend unambiguous support to the view that a single fracture cannot be considered diagnostic of impact [for a discussion, see 11] since other processes can produce similar features. Our experiment also shows that breaks that have occurred post-retouch cannot be straightforwardly interpreted as caused by impact [cf. 107] in assemblages that could include armatures produced by deliberate snapping. We segmented already backed bladelets into shorter elements, and the breaks–some of which qualify as classic DIFs (Fig 8E above)–consequently postdate the retouch (backing).

The data recorded here indicates that size cannot be straightforwardly used as a criterion for filtering out production-related secondary damage in the archaeological data even if artefact dimensions are relatively standardised. The location and attributes of the secondary scars may better help distinguish between scars formed on impact and those resulting from intentional snapping of backed elements. In the intentionally snapped sample, scars located on the surface of break initiation are rare, as are scars with destroyed initiations. These two characteristics testify to significant compression at the moment of breakage [cf. 14], which is typical of projectile impact but rarer in intentional snapping where bending forces dominate. While these feature attributes alone are unlikely to yield reliable projectile identifications, when examined as parts of feature combinations they can be helpful clues to the amount of compression that was involved in the breakage of the lithic artefact.

Previous experimental results likewise give reason to argue that production-related breakage can be a complex phenomenon and produce fractures that mimic impact damage. One very illustrative example is the breakage pattern documented in an experiment by O’Farrell and Pelegrin where backing by pressure produced an accidental bending break with a very long removal initiated on the break surface and located on the ventral surface where the break initiates [112: plate VI.23]. While we suspect that this ventral scar may be due to direct contact with the pressure flaker and would therefore not count as a true secondary scar (formed as the result of contact between the two armature fragments under axial stress), the depicted wear pattern resembles impact damage. This means that some of the criteria highlighted by us here–particularly location of secondary damage with respect to break initiation–may be limited to intentional snapping and therefore not universally applicable. Such criteria need to be constructed case by case, taking into consideration the *chaîne opératoire* of the archaeological artefacts. From these results, it is clear that tailored projectile experiments that include careful evaluation of production-related features are required for reaching reliable projectile identifications archaeologically.

### 5.2 Screening results

#### 5.2.1 Level 3

As already noted in previous studies, the Level 3 backed tool collection consists mostly of artefacts of small to very small size. Among the finished objects in the collection, there are Gravette points that show impact damage obvious to the naked eye, but these larger points are not frequent and the majority of the artefacts with a pointed design are best described as microgravettes. Some of these are very narrow and fine. Fragmentary objects are numerous. A distinctive feature of the assemblage is the presence of extremely small backed tools that can be termed nanogravettes although they appear less constrained in design (qualities of additional retouch) than the larger microgravettes that typically show characteristic inverse retouch at the proximal extremity. The first screening identified a number of fracture combinations strongly indicative of impact, but a large majority of the material showed uncharacteristic breaks.

The measurement data acquired on a sample (n = 113) of backed tools during screening was used to group the material in the following three size groups: 1) implements narrower than 4mm following previous studies [27] to account for the smallest fraction (“nanogravettes”), 2) those with a width between 4mm and 8mm (“microgravettes”), and 3) those with a width equal to or higher than 9mm following the possible subtle clustering observed in visualising the measurement data (“Gravettes”). This grouping remains arbitrary and only serves the purpose of allowing initial comparisons between broad size groups. The details are discussed in S4 Appendix.

**Fig 10:**
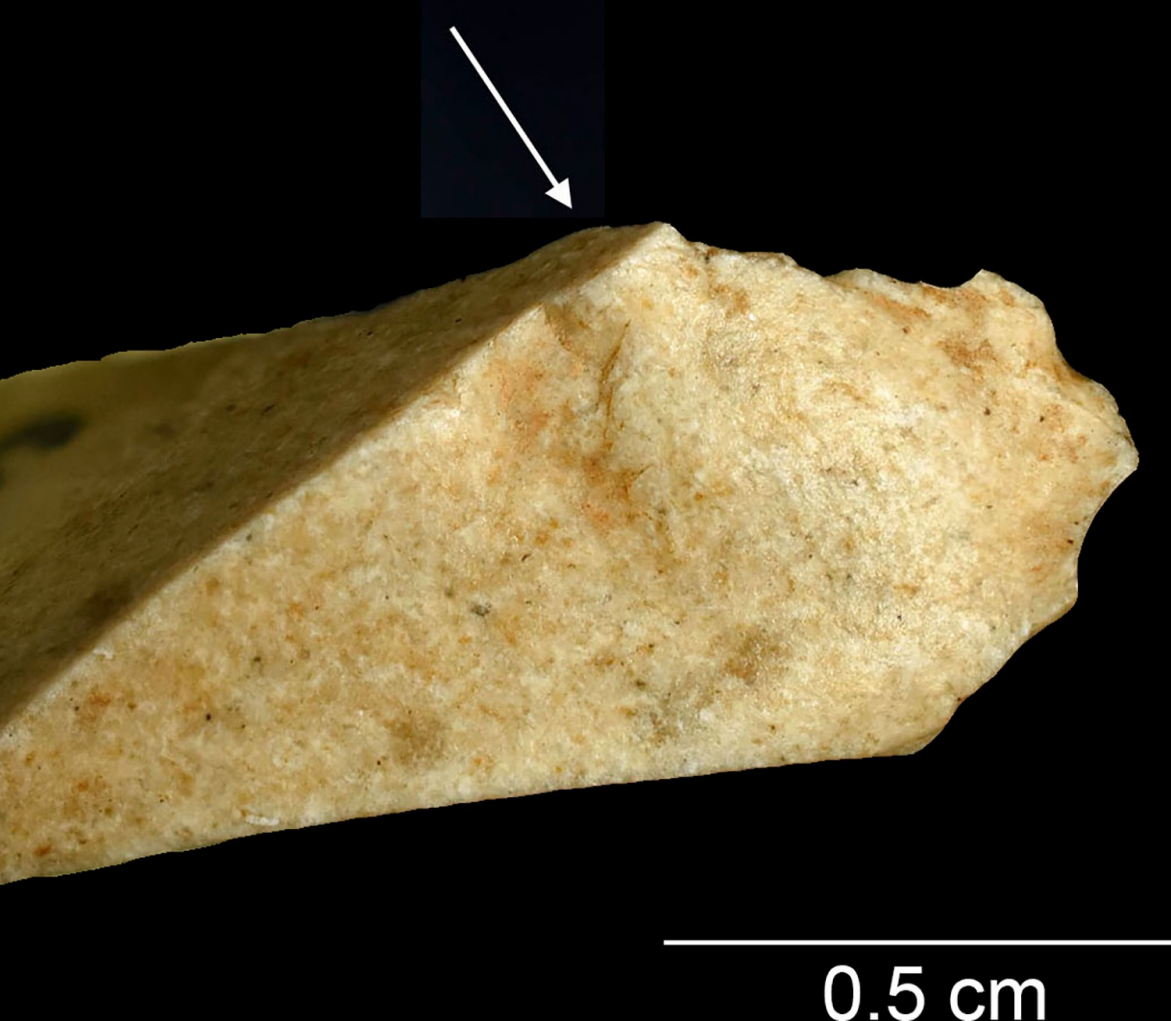
A backing break with a cone initiation (arrow) and a clear bulb on AP/58-3-1473.

Using this size grouping, the artefacts in the background sample can be divided into preliminary break categories (Table 6). Breaks that had a clear cone initiation on the surface from which backing removals initiate (Fig 10) were classified as definite backing breaks [see 112 for comparable observations], whereas similar breaks with more diffuse initiations, as well as oblique and/or slightly twisted snaps, were counted as tentative ones. The cone initiations are consistent with the recent interpretation, also relying on scar attributes, that some of the backs were produced by direct percussion using (soft) stone hammers [87]. Importantly, the ‘impact breaks’ singled out at this stage of the analysis are only *indicative* of projectile use and required supporting evidence to be confirmed (see below).

**Table 6:** Preliminary classification of the breaks observed in the Level 3 backed tool sample for which basic recording was done.

	Impact	Backing accident	Other product-ion	Tapho-nomic	Mixed	Indet	Total
Gravette	9 (5)	2 (0)	3 (0)	0 (0)	0 (0)	6	20
Microgravette	36 (17)	8 (8)	0 (0)	2 (0)	9 (0)	56	111
Nanogravette	11 (7)	4 (3)	0 (0)	0 (0)	2 (0)	46	63
Bitruncated backed piece	1 (0)	0 (0)	1 (0)	0 (0)	0 (0)	2	4
Truncated backed piece	2 (2)	0 (0)	0 (0)	0 (0)	0 (0)	6	8
Production waste	0 (0)	0 (0)	53 (0)	0 (0)	0 (0)	0	0
Indet	13 (9)	1 (1)	4 (1)	0 (0)	6 (0)	46	70
**Total**	**72 (40)**	**15 (12)**	**61 (1)**	**2 (0)**	**17 (0)**	**162**	**329**

The counts in brackets indicate the proportion of tentative identifications.

A majority of the breaks fall in the category ‘indeterminate’, which dominantly includes snap breaks that were in most cases suspected to be production-related or taphonomic but for which use could not be ruled out as a cause. Reliable identification of taphonomic breaks by macroscopic criteria alone was a challenge, and only two were finally recorded: one clearly recent break and one heat fracture. This count must therefore be treated as an underestimate. Category ‘mixed’ concerns pieces with two (or, in rare cases, several, meaning two overlapping breaks at the same extremity) breaks that represent different categories.

The results of the preliminary analysis indicate that the Level 3 backed tool collection is shaped by various processes, including tool manufacture, use, and taphonomic events. Projectile use was evident in a part of the assemblage already from the first screening, whereas indications of other uses were tentative at best, consisting mainly of minor lateral scarring that could not be attributed to a particular function and could also result from projectile use.

#### 5.2.2 Level 2

The manufacturing technique of backed bladelets, based on intentional snapping of the blanks [101, 103], has created a collection that consists largely of backed bladelet fragments with a clean break at both ends (Figs 3 and 4 above). The material was divided into six main screening categories on the basis of macroscopic observations and selective use of the stereomicroscope (6.6–40*×*) that provide a first idea of the frequency and characteristics of functional wear (Table 7) (for classification details, see S1 Appendix).

**Table 7:** The screened Level 2 backed piece collection divided into preliminary categories according to the presence/absence of use-related damage and technological characteristics.

Screening category	n	%
Break(s) suggestive of impact	102	14,7%
Substantial edge damage	104	15,0%
Minor edge damage	220	31,7%
No edge damage or isolated minuscule scars	49	7,1%
Production waste	190	27,4%
Indet	28	4,0%
**Total**	**693**	**100,0%**

‘Production waste’ includes seven tentative interpretations.

Pieces without potentially functional fractures are in the minority, which is consistent with the earlier interpretations [101, 103] that despite the low frequency of bitruncated backed elements, a large part of the collection can be considered used artefacts, be it intentionally snapped or broken during or after their use. Evidence suggestive of projectile use was particularly abundant. During screening, a number of artefacts with minor edge damage (subtle forms of scarring on the cutting edge) were briefly examined with high magnification for potential knife wear, but no conclusive evidence was found.

Techno-morphologically speaking, the screening confirmed the previously reported complete absence of intact Gravette and microgravette points. A limited number of pieces (n = 41 out 639 screened) showed additional retouch besides the back and possible truncation. In some cases, this resembles microgravette retouch in that it is found at one of the extremities and is inverse, but since this kind of retouch sometimes (n = 5) co-occurred with a truncation, it cannot be considered by itself indicative of the presence of fragmentary microgravette points in the sample. The assemblage represents at least dominantly, if not exclusively, microliths and their production waste.

### 5.3 Evidence of projectile use

**Fig 11:**
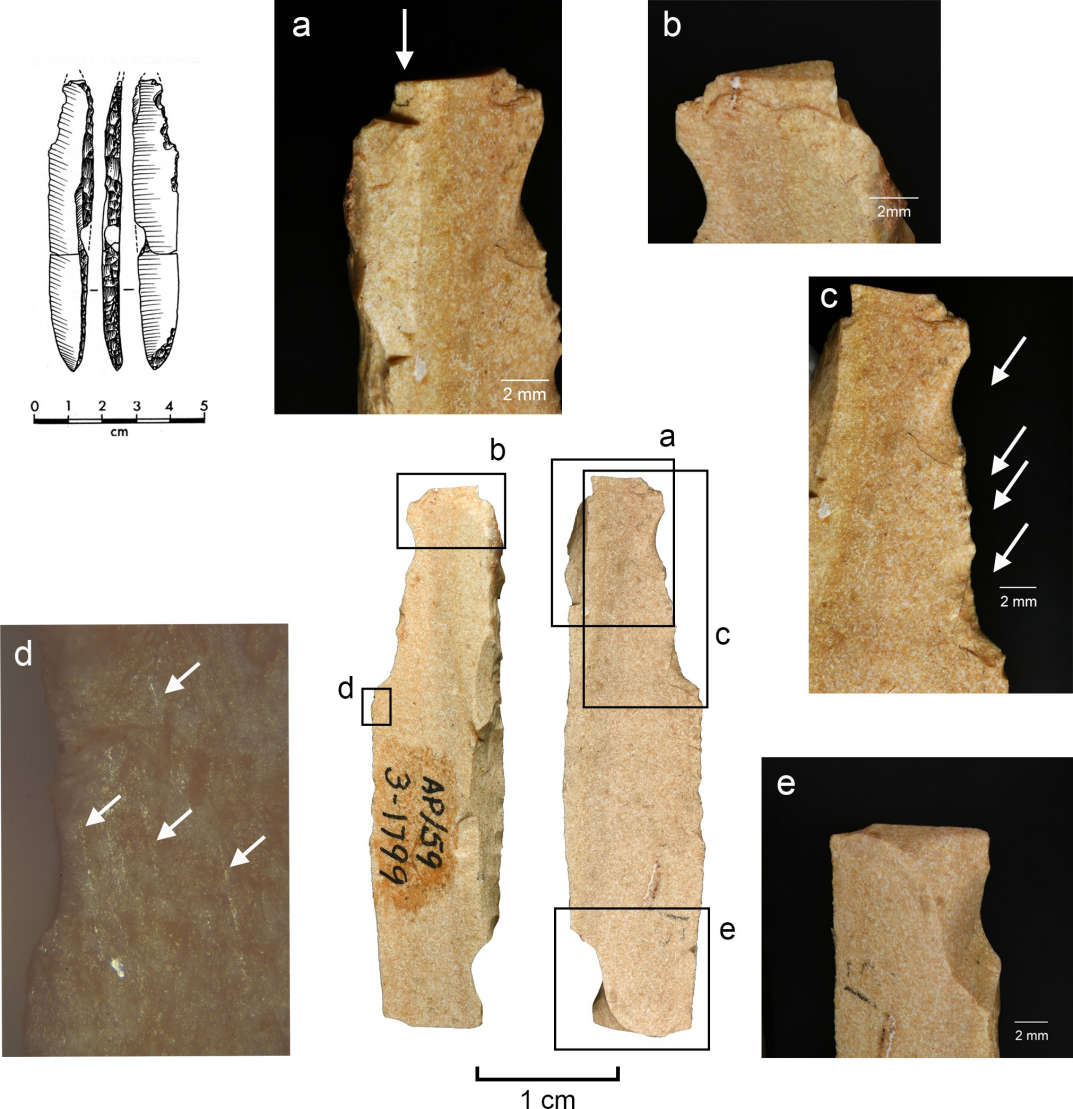
Evidence of impact on Gravette point from Level 3 (AP/59-3-1799, Bergerac flint). a. An elongated secondary removal ("burination") initiated on a break (b); b. Bending-initiated break with a short propagation and a fissured step termination at the distal end of the armature, associated with secondary damage on the surface of initiation (a); c. Obliquely oriented bending-initiated lateral scarring (direction indicated with arrows) on the ventral aspect of the cutting edge, showing dominantly stepped, partly fissured terminations; d. MLITs (indicated with arrows) associated with lateral scarring on the dorsal aspect of the cutting edge (50×); e. Proximal break with a bending initiation on the cutting edge and feather termination on the backed edge.

The detailed functional study that employed low and high magnification analysis found frequent evidence of projectile use in both samples. Fig 11 shows a Gravette point from Level 3 with particularly abundant impact damage and serves as an example of feature combinations that yielded the most confident projectile identifications in the present study.

We argue that relying on combinations of wear features [cf. 11, 14] allowed singling out the projectiles with the strongest evidence and thus guaranteed that the subsequent discussion built on artefacts for which projectile function could be confirmed with a high degree of certainty.

#### 5.3.1 Level 3

**Fig 12:**
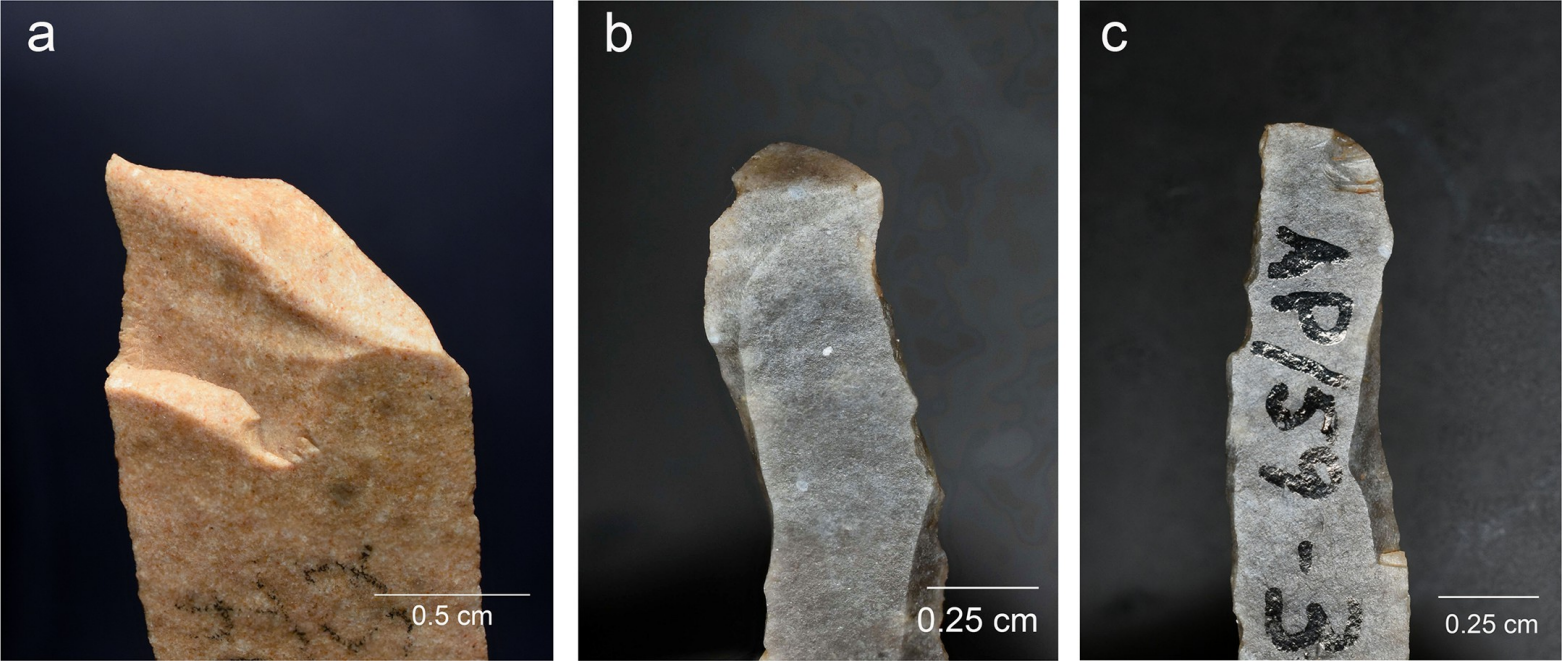
Bending breaks on Level 3 armatures. a. A dorsally initiated break terminating in a fissured step (fissure marked with arrow) on Gravette point AP/59-3-1798; b. A bending break initiated on the backed edge and twisting onto the ventral aspect of the opposite (cutting) edge on microgravette AP/59-3-1731 (Senonian flint); c. Fissured step termination of the break shown in b. on the cutting edge of the armature (fissure indicated with arrow). Note the large secondary scar initiated on the break surface.

**Fig 13:**
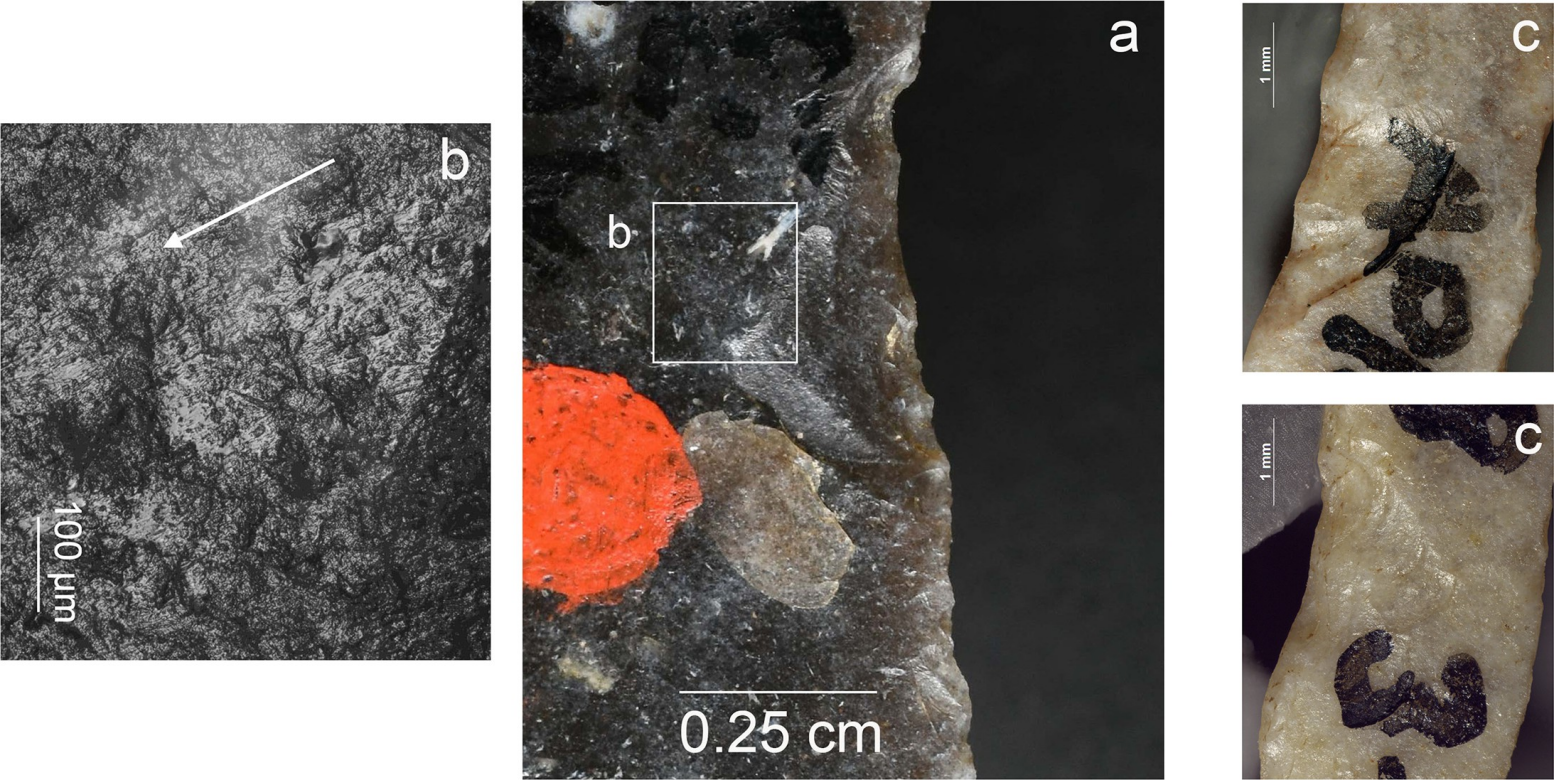
Impact damage (lateral removals) on Level 3 artefacts. a. Obliquely oriented lateral scarring and a nearly detached secondary scar on AP/58-3-847 (truncated backed bladelet, Senonian flint); b. Friction polish associated with the damage shown in a., created by the detached chips (linearity marked with an arrow); c. Two locations with invasive lateral scarring on a very narrow backed bladelet (“nanogravette”) AP/58-3-1210.

**Fig 14:**
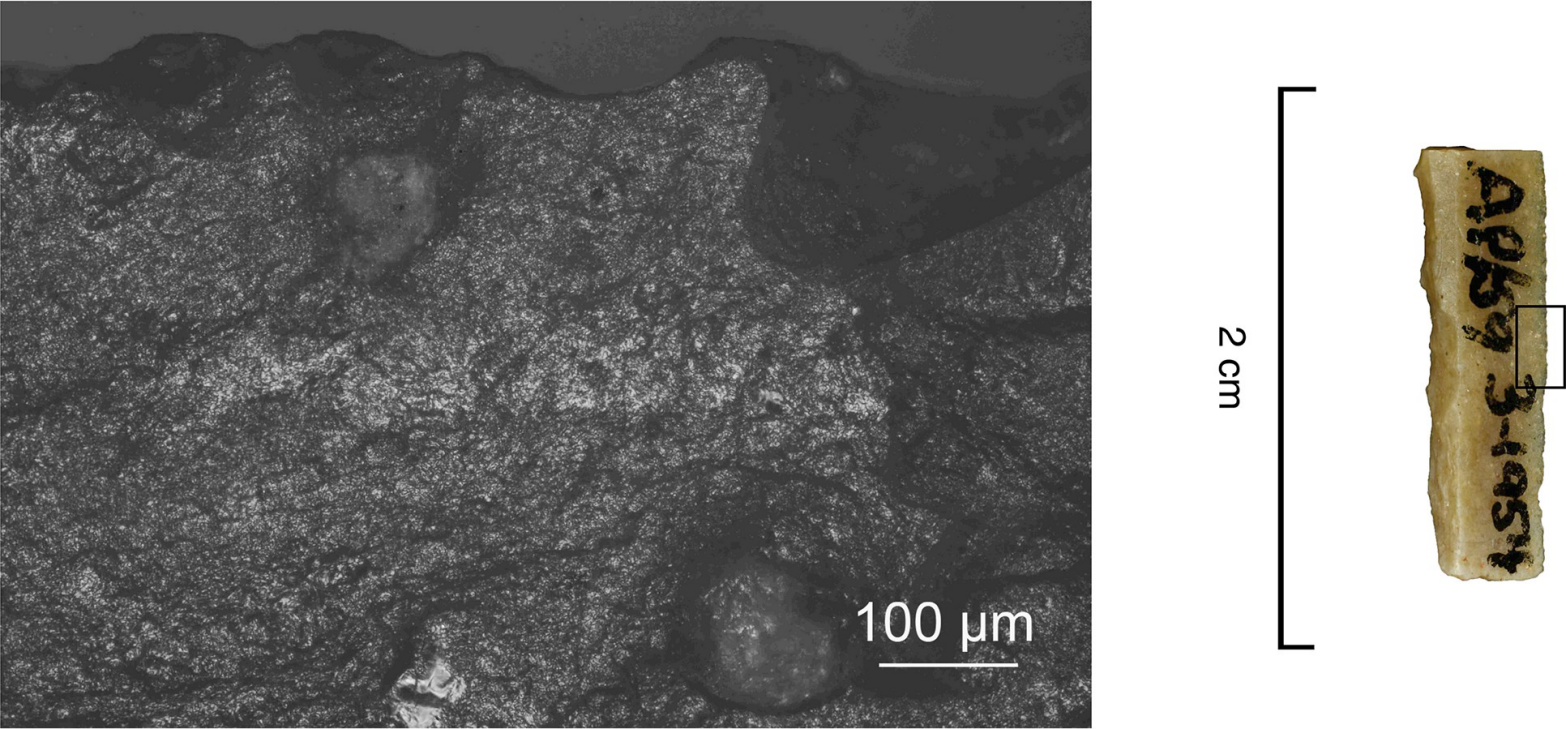
MLITs associated with minor edge damage on truncated backed piece AP/59-3-1954 (Senonian flint).

Out of the 104 pieces for which damage patterns were recorded in detail, a total of 39 showed a set of features that allowed identifying them as projectiles with certainty. The feature patterns consisted mostly of combinations of bending-initiated breaks with a long propagation, associated secondary scarring, and obliquely oriented lateral scarring. Examples are shown in Figs 12 and 13. Clear MLITs (Fig 14) were very infrequent in the Level 3 sample and were only observed on two pieces analysed with high magnification (n = 66), with a further five pieces showing faintly visible linear features whose link to use could not be confirmed with certainty. It is probable that surface preservation played a role here. Consequently, projectile identification in this sample relied mainly on combinations of macroscopic (low magnification) features.

The frequency of confident projectile identifications in each backed tool category is presented in Table 8. This was a targeted, non-random sample that included mostly pieces that were considered to have high potential for projectile use in screening, and does not therefore reflect the frequency of impact wear in the entire collection. In addition to the counts presented in the table, a total of 83 artefacts (four Gravette points, 36 microgravettes, 14 nanogravettes, three truncated backed pieces, 19 indeterminate backed pieces, and six artefacts that showed more ambiguous shaping but were nevertheless stored with the backed tool material) could be considered as possible projectiles, but when the strict criteria chosen here are applied, the evidence can be considered insufficient. Only the pieces with solid evidence (cf. Table 8) are considered in further discussing damage patterns and their implications.

**Table 8:** The number of confident projectile identifications in the Abri Pataud Level 3 backed tool sample.

Artefact category	n	total analysed
Gravette	4	9
Microgravette	18	44
Nanogravette	6	21
Truncated backed piece	4	9
Indet	7	21
**Total**	**39**	**104**

While the frequencies are lower among the nanogravettes and pieces that could not be attributed to any of the main categories (i.e. mainly medial fragments without characteristic shaping), it is clear that all categories included here show evidence of projectile use.

The frequency of different macroscopic wear features on the artefacts that could be identified as armatures with a high level of confidence are summarised in Table 9. Lateral scarring is a recurrent form of damage in all projectile categories, matched in frequency only by bending breaks which here include snap breaks and other uncharacteristic breaks that can result from a variety of processes, including production and trampling (see above). While lateral scarring needs to be accompanied with other features to qualify for evidence of projectile use (see e.g. the examples in Figs 13 and 14 above), it is in this sense not different from other forms of damage that are also by themselves not enough for reliable identifications [see 11]. The dataset here shows that lateral removals can be valuable clues to this kind of use in an archaeological assemblage. Breaks initiating and terminating on an edge are particularly frequent among the microgravettes. Secondary damage (scars initiated on a break surface) associated with bending-initiated breaks is also relatively frequent.

**Table 9:** Frequencies of macroscopic features on the Level 3 armatures identified with a high degree of confidence.

Feature category		Gravette (n = 4)	Micrograv. (n = 18)	Nanograv. (n = 6)	Truncated (n = 4)	Indet (n = 7)
Bending breaks	Surface-to-surface	4	12	5	3	5
	Edge-to-edge	1	7	2	0	0
	Indet	1	1	0	0	3
Secondary damage		4	9	4	1	6
Lateral damage		3	12	4	4	7

‘Surface-to-surface’ breaks refer to bending breaks initiated on a surface and terminated on the opposite surface whereas ‘edge-to-edge’ breaks are initiated and terminated on a lateral edge. ‘Secondary damage’ refers to scars that are associated with bending breaks and initiate on the surface formed by the break. ‘Lateral damage’ refers to obliquely oriented scarring on the cutting edge of the armature.

Examination of the propagation length of breaks and scars on the projectiles revealed that the length is primarily affected by point morphology and fracture location (S5 Appendix). These results give reason to argue that measurement data should not be used uncritically in projectile studies and that establishing minimum values based on armature size does not appear to be enough of a precaution. Feature measurements were not included here in the set of diagnostic criteria.

#### 5.3.2 Level 2

The low and high magnification samples from Level 2 overlap to some extent: of the 66 pieces for which damage was recorded in detail, 25 became analysed under high magnification. The remaining 16 pieces in the high magnification sample mostly represent artefacts with subtle forms of microwear, including pieces with minor lateral scarring (n = 11) or no scarring at all (n = 1). These were selected in an attempt to detect possible non-projectile use in the assemblage. Substantial edge damage is represented by three pieces in addition to which a single piece with a suspected impact break was included.

The sample shows a bias towards probable projectile armatures, and is therefore not used for drawing conclusions of the functional variability of the entire assemblage. The sample for a detailed recording of break and edge damage data (low magnification sample) was selected by including tools mainly from the three main screening categories that were judged to represent use-wear (categories 1–3 in S1 Appendix). Forty-seven pieces initially judged as showing an impact break (often in combination with edge damage) became analysed, together with 28 showing substantial edge damage, 23 with minor edge damage, and seven with either no edge damage or only isolated minuscule scars.

When classified using the same criteria as for Level 3, altogether 47 of the 66 pieces for which damage was recorded in detail in the Level 2 sample could be identified as projectile armatures with certainty. In addition, two pieces in the high magnification sample for which damage was not recorded in detail proved to have MLITs, bringing the total of positive projectile identifications up to 49 in the combined sample. The two denticulated pieces in the sample were interpreted as probable projectiles (see S6 Appendix).

**Fig 15:**
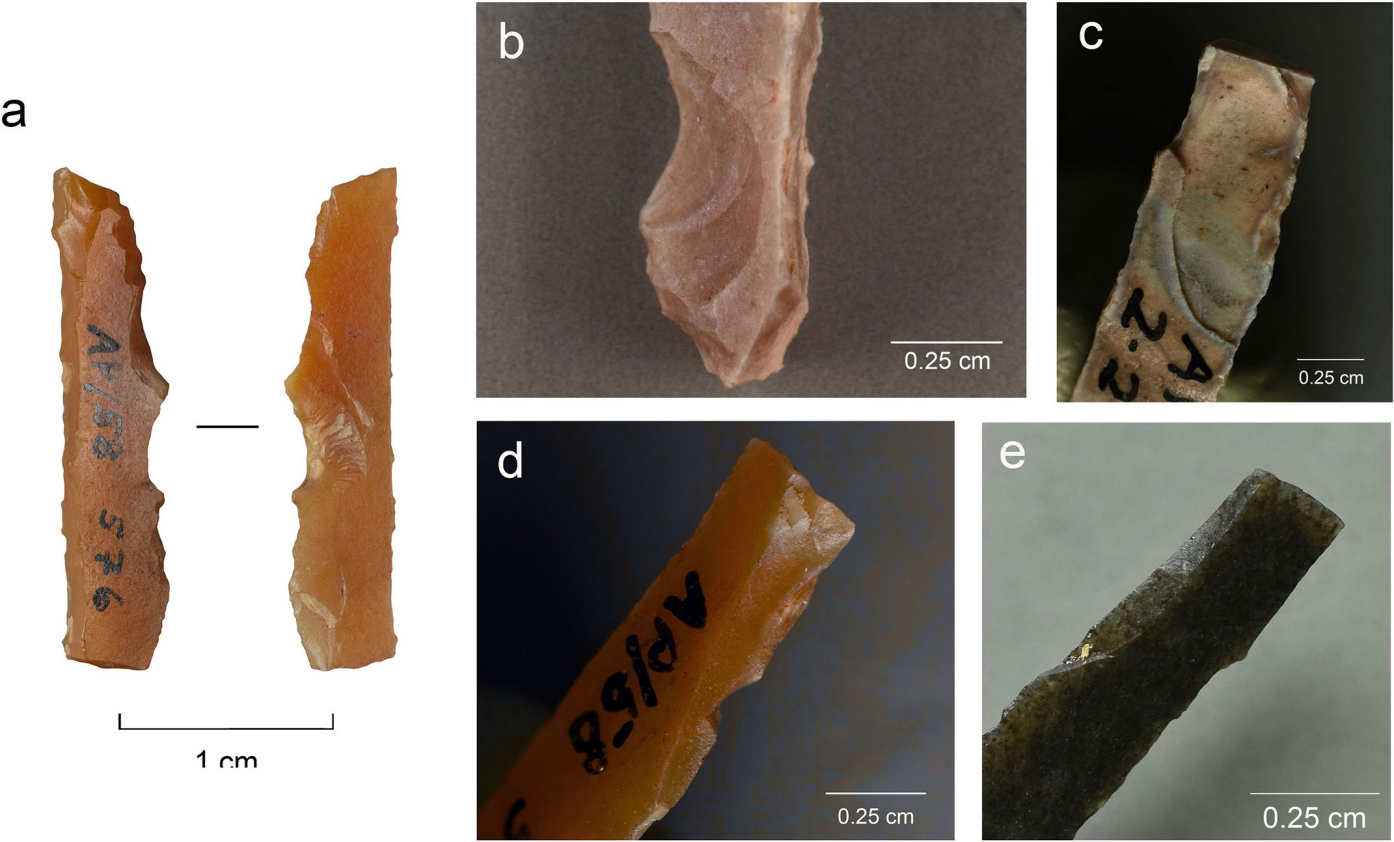
Examples of lateral damage and other impact scars on Level 2 armatures. a. A bending-initiated break and heavy bifacial edge damage showing two main orientations on AP/58-2-576 (Senonian flint); b. An invasive obliquely oriented scar on AP/58-2-862 (Senonian flint); c. A perpendicular removal with an overshot termination on AP/63-2-2064; d. A longitudinal removal (burination) on AP/58-2-595 (Senonian flint); e. A longitudinal removal (burination) on AP/58-2-610 (Senonian flint).

Obliquely and perpendicularly oriented lateral scarring is the most characteristic form of impact wear in the Level 2 assemblage (Table 10). It is relevant to note here that lateral scarring is not standardly considered in studies that focus on DIFs [8], which means that the DIF approach would be likely to miss many of the armatures in the current set. The removals are often invasive and easily identifiable without a microscope, and in some cases even overshoot the ridge formed by the back and the adjacent surface. In at least one case a secondary scar associated with a lateral removal was registered, similar to AP/58-3-847 from Level 3 described above, and suggestive of strong compression at the moment of breakage. Also more subtle forms of lateral removals were documented and could be attributed to projectile use with relative certainty in cases where the pieces were examined under high magnification and no evidence of repetitive cutting motion (knife use) was encountered. In addition to the removals initiating on one aspect of the cutting edge, some of the artefacts show elongated removals that start at one extremity and terminate on the lateral edge (so-called impact burinations) (Fig 15).

**Table 10:** Frequencies of macroscopic features on the Level 2 armatures identified with a high degree of confidence.

Feature category		n	out of	% of pieces
Bending breaks	Surface-to-surface	41	47	87.2%
	Edge-to-edge	5	47	10.6%
	Indet	5	47	10.6%
Secondary damage		27	47	57.4%
Lateral damage		45	47	95.7%

The two pieces for which macrofractures were not recorded in detail are left out even though they have MLIT). ‘Secondary damage’ refers to scars that are associated with bending breaks and initiate on the break surface. ‘Lateral damage’ refers to scarring on the cutting edge of the armature. Note that the high frequency of surface-to-surface breaks is most likely affected by production-related breaks (intentional snapping).

**Fig 16:**
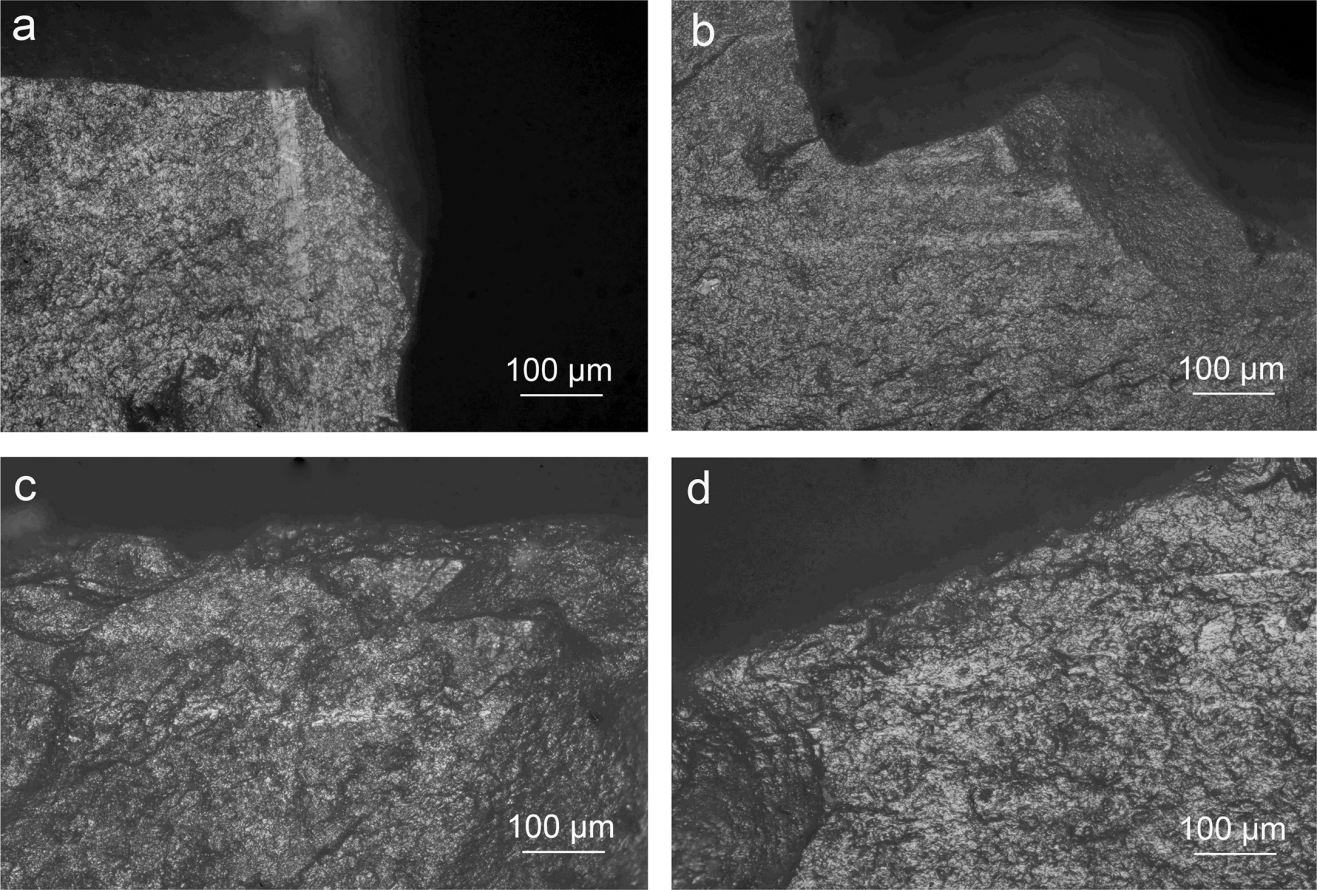
MLITs on the Level 2 backed pieces. a. Ventral distal extremity of AP/89-2-261, associated with a snap break; b. Ventral distal right edge of AP/58-2-938, associated with lateral removals; c. Dorsal distal left edge of AP/58-2-801, associated with lateral removals; d. Ventral medial right surface of AP58-2-595, associated with secondary scars. All 200×.

MLITs were in general slightly more frequent in the Level 2 sample than in the Level 3 sample. Convincing examples were found on a further five pieces in addition to the two mentioned above, bringing the total to seven, and tentative ones on four more artefacts. The best examples are most often associated with lateral scarring, dominantly scars that are oriented obliquely to the edge, but in one case also a snap break (Fig 16) and in another with a patch of scars with a perpendicular orientation. Despite these occurrences, majority of the projectiles were identified on the basis of macroscopic fractures (Table 10). Some of the armatures showed indications of repeated use and/or reworking after having been damaged (S7 Appendix).

To help estimate what portion of the breaks with secondary damage might be due to production in the present sample, the snap breaks in Level 2 and Level 3 samples were divided into those where the secondary removals are located on the surface of the break’s termination (as can be expected from intentional snapping) and into those where they are located elsewhere (on the surface of initiation or on a lateral edge, which here usually meant the backed edge). These frequencies for each projectile sample are shown in Table 11. The remaining part of both samples, i.e. artefacts where the evidence was not enough for secure projectile identifications, is included for comparison. The high frequency of secondary scars on the surface of break’s termination in the part of the Level 2 that does not show strong evidence of projectile use is supportive of the hypothesis that part of the secondary scarring may be due to production. Interestingly, Level 3 shows an inverse pattern, with locations other than the surface on which the break terminates dominating in both categories. This could imply a relatively higher proportion of use-related secondary damage in this sample as opposed to the Level 2 one. The counts for Level 2 give reason to treat the break and secondary damage data presented above with some caution in terms of the origin of some of the features. Yet including all the breaks with their associated secondary damage in the counts is the most straightforward way to present the observations on this collection since the true proportion of production-related breaks and damage remains unknown. Table 11 provides a background against which to judge this data.

**Table 11:** Location of secondary scarring associated with the snap breaks with respect to the break’s termination.

Location of secondary scarring	Level 3				Level 2			
	Projectiles		Remaining sample		Projectiles		Remaining sample	
	n	%	n	%	n	%	n	%
Same as break’s termination	17	41,5%	8	32,0%	19	57,6%	5	71,4%
Other	24	58,5%	17	68,0%	14	42,4%	2	28,6%
**Total**	**41**	**100,0%**	**25**	**100,0%**	**33**	**100,0%**	**7**	**100,0%**

**Fig 17:**
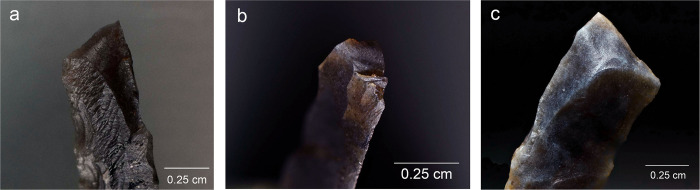
Bending breaks on Level 2 armatures. a. A ventrally initiated step-terminating break associated with lateral scarring on AP/58-2-147 (Senonian flint); b. An edge-to-edge break with a fissured termination on AP/58-2-507 (Senonian flint); c. A ventrally initiated break with a shallow step termination on AP/89-2-54 (Senonian flint).

The terminations of bending breaks with long propagation on a dorsal ridge can be very narrow and therefore poorly visible without a stereomicroscope. Ventrally terminated breaks are often shorter but can have long fissures indicative of relatively strong compressive force (Fig 17).

As regards secondary damage associated with the breaks, it was often not possible to determine with certainty whether the small removals initiating on an earlier break surface were true secondary scars formed (almost) simultaneously with the break, or whether the break and the scars represented independent events (e.g. a snap formed during production and removals formed through contact with the shaft, the target, or other armatures during use). The count of actual secondary scars may therefore be lower than the one reported here. It also needs to be kept in mind that secondary scars can also form during the intentional or accidental breakage of small backed tools (see above). The present sample nevertheless includes combinations of features that are easier to attribute to use than production, such as a heavily oblique and twisted dorsally initiated snap break associated with secondary removals on both surfaces on AP/63-2-1922 that in addition shows oriented lateral edge damage highly suggestive of impact.

### 5.4 Tips or lateral inserts?

The detailed recording of impact break and scar attributes allows comparing patterns between the samples and pushing the analysis one step beyond the identification of armatures. The recorded data is used here to reconstruct the position of the armatures in their shafts (distal versus lateral) to address the question of overall weapon design in the Recent and Final Gravettian of Abri Pataud.

Due to their suggestive morphology, Gravette and microgravette points have been commonly perceived as weapon tips, and experiments have confirmed their efficacy when hafted this way [8, 57, 58, 121] although lateral hafting of microgravettes has sometimes also been hypothesised [68, 69, 112] At Pataud, the data presented above allows confirming that laterally hafted armature elements are present in both Level 3 and Level 2 collections. The most obvious argument for this is the presence of convincing impact wear on bitruncated backed bladelets.

The challenge that the Pataud collection poses is that the material, particularly in Level 3, is highly fragmentary, and that truncated pieces are rare. The frequency of lateral hafting cannot therefore be investigated solely by examining the morphology of the artefacts with impact damage. For the majority of the collection, other methods were needed for reconstructing the position and alignment of the armatures in their shafts. Two main criteria are proposed here for distinguishing between distally and laterally hafted armatures: the characteristics and relative frequency of lateral scarring, and bending break properties, including the location of initiation. The following discussion only concerns artefacts with the strongest evidence of impact (i.e. pieces identified as certain projectiles).

#### 5.4.1 Characteristics and frequency of lateral scarring

Qualitatively speaking, certain characteristics of the lateral damage observed on Level 2 armatures lend support to the interpretation that these lithic artefacts were hafted as side elements. We propose here that particularly lateral removals with overshot terminations (see Fig 15C above) could be indicative of lateral hafting. These kinds of removals are absent in our current sample of experimental microgravettes hafted as weapon tips, and present in the Pataud Level 2 sample. While they are not frequent, occurring only on 6 of the artefacts for which damage was recorded in detail, on an assemblage scale they can be considered indicative of the presence (if not frequency) of laterally hafted elements. While the formation of such removals needs to be investigated through further experiments, the mechanical explanation we propose here is that for these removals to form, the back of the artefact needs to be firmly supported by the weapon shaft; otherwise forces acting obliquely or perpendicularly to the cutting edge would produce a break rather than invasive lateral removals.

**Fig 18:**
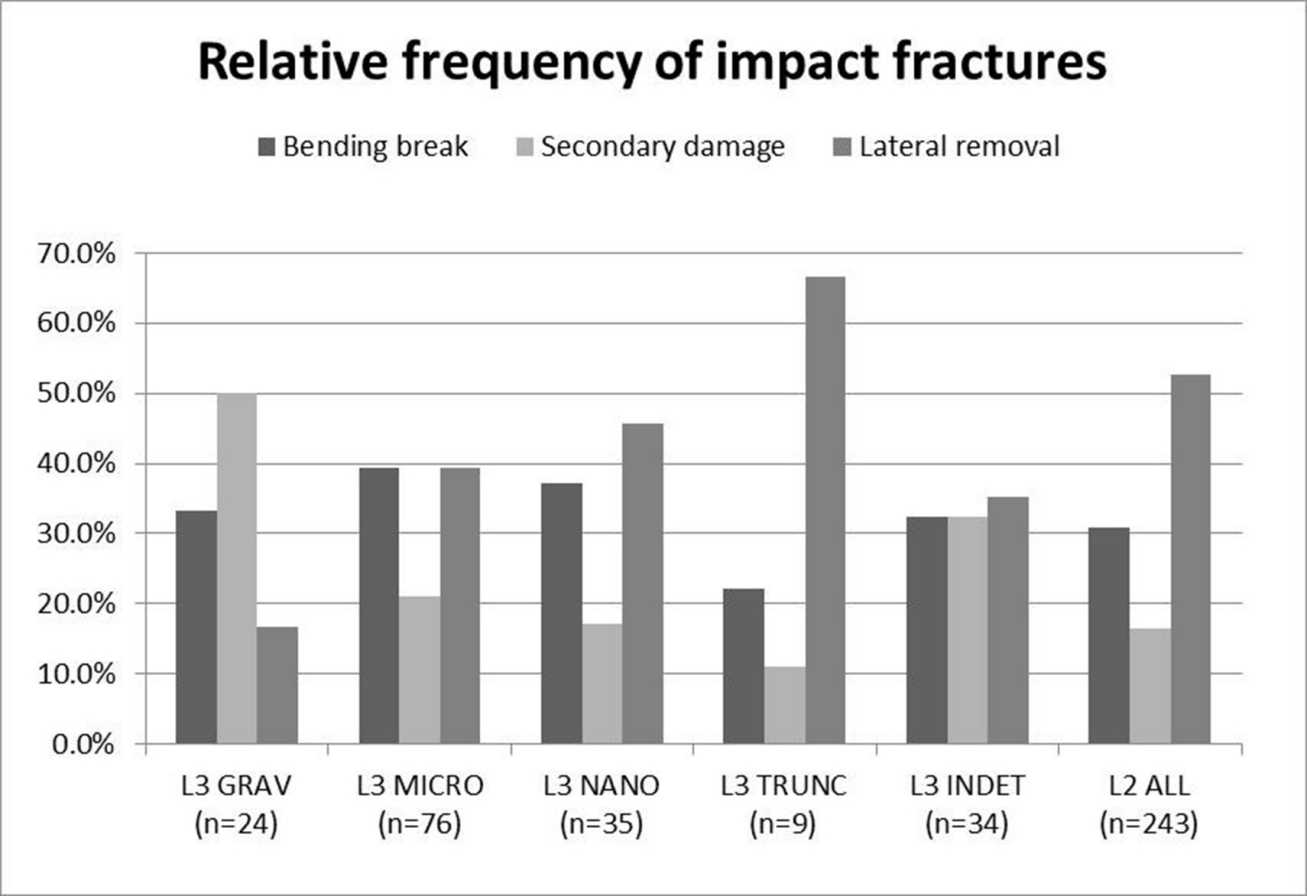
Relative frequencies of main impact damage categories in the Abri Pataud Level 3 and 2 samples of artefacts with strong evidence of projectile use. Note that the number of observations for the Level 3 truncated backed pieces is very low. L3: Level 3, L2: Level 2, GRAV: Gravette point, MICRO: microgravette, NANO: nanogravette, TRUNC: truncated piece. For data, see S9 Appendix.

Taking into consideration the fact that lateral scarring is extremely frequent in the Level 2 assemblage that is also rich in truncated backed bladelets, and the earlier observations that this type of damage dominates on barbs and other laterally hafted inserts [105, 122], it can be hypothesised that the Level 2 assemblage would contain relatively high proportions of laterally hafted armatures. To see whether a true difference existed between Level 2 and Level 3 in this respect, we calculated the relative frequencies of lateral damage and other main impact fracture categories for all projectile categories (Fig 18 and S8 Appendix). The Level 2 sample dominantly consists of medial fragments of backed bladelets, and obvious pointed forms are lacking. The sample is treated here as a single unit to make comparisons with Level 3 simpler because the differences in the relative frequencies of main feature categories are minor between the truncated and non-truncated artefacts from Level 2 (S8 Appendix) and may be partly affected by the difference in sample sizes. The Level 3 sample was divided into broad morphometric categories employed in the study to be able to detect variability between them.

If the Level 3 truncated backed piece sample that is too small for the percentages to be reliable is ignored, the Level 2 sample shows the heaviest dominance of lateral removals, consistent with the impression gained early in the analysis. This sample is the one most likely to show a mix of production breaks (intentional snapping) and impact damage in the bending break category. The relative frequency of lateral scarring may therefore be even higher within these groups than the graph indicates. Since production-related breaks are not limited to snaps and can also show secondary scarring (see above), there was no straightforward way to filter out the suspected non-impact breaks from the dataset without risking errors. Therefore all recorded features are included here in the counts.

**Fig 19:**
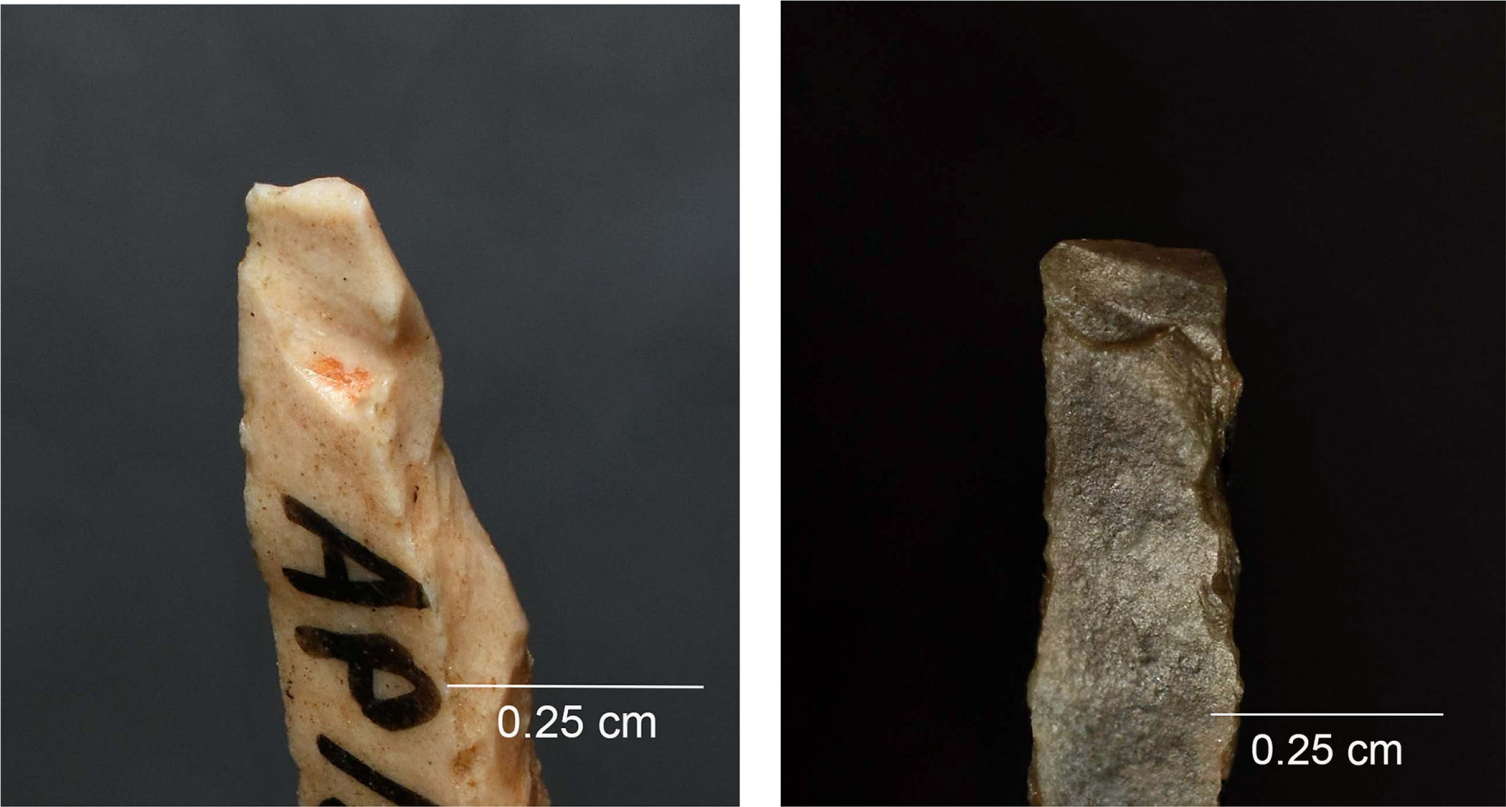
Breaks on two artefacts interpreted as laterally hafted armatures from Level 3. Left: a small dorsally bending-initiated feather-terminating break on the tip of AP/58-3-400; right: a dorsally bending-initiated step and fissure terminating break on AP/58-3-1363 (Senonian flint). Note that in the absence of MLITs, the breaks could also be the result of production. The projectile identifications are based on combinations of features (e.g. lateral removals).

When the Gravette samples from Level 3 are compared with the Level 2 data, it appears that the relative frequency of lateral scarring increases when armature size decreases. This signal should be treated somewhat cautiously given that the samples from the largest and the smallest size groups are very small (observations on four and seven artefacts, respectively). Yet, if all other things were kept constant, it would seem counterintuitive that nanogravettes showed a lower relative frequency of bending breaks than microgravettes (or Gravettes) since less energy is required to break a small piece than a large one [cf. 66, 112, 123]. The diminishing portion of bending breaks (and secondary damage) can therefore be taken as an indication of a higher frequency of laterally hafted armatures among nanogravettes than microgravettes. This is in agreement with the pure common-sense observation that most of the nanogravettes appear too fragile to serve as weapon tips and would better function as lateral inserts on composite points. Also the rather unimpressive bending breaks located at the extremities of some of these armatures (Fig 19) are more consistent with what is seen in Level 2 than with the sometimes dramatic forms of breakage observed on the Level 3 microgravettes and Gravettes. The fact that the overrepresentation of lateral damage is not as evident in the nanogravette sample as in the Level 2 one can potentially be explained by the smaller dimensions of the former. Small armatures break with less energy than large ones, and while the Level 2 armatures are not particularly large, their widths range from c. 2.5mm to almost 7mm and thicknesses from less than 1mm to c. 3mm whereas the nanogravette group was arbitrarily limited to artefacts narrower than 4mm (with thickness values comparable to the Level 2 sample). Breakage due to a variety of processes can be expected to be more frequent among them and increase the proportion of bending breaks, thus bringing down the relative proportion of lateral damage.

When viewed this way, the pattern presented in Fig 18 can be explained by differences in the hafting of different groups of armatures. Yet it has to be kept in mind that other factors, such as the weapon delivery system used, may lead into differential patterning between samples even if the hafting system is kept constant [14]. Also details of hafting, including the qualities of the glue [cf. 124], are likely to have an effect on impact fracture patterns as the kinetic energy released on impact results in the breakage of the weakest element of the weapon unless all the energy is absorbed by the target [11, 113]. Determining the dominant variables responsible for the patterns observed here requires further experimentation. Until this reference data becomes available, other lines of evidence are required to argue for particular projectile point designs in the present assemblage.

#### 5.4.2 Bending break initiation and other characteristics

**Fig 20:**
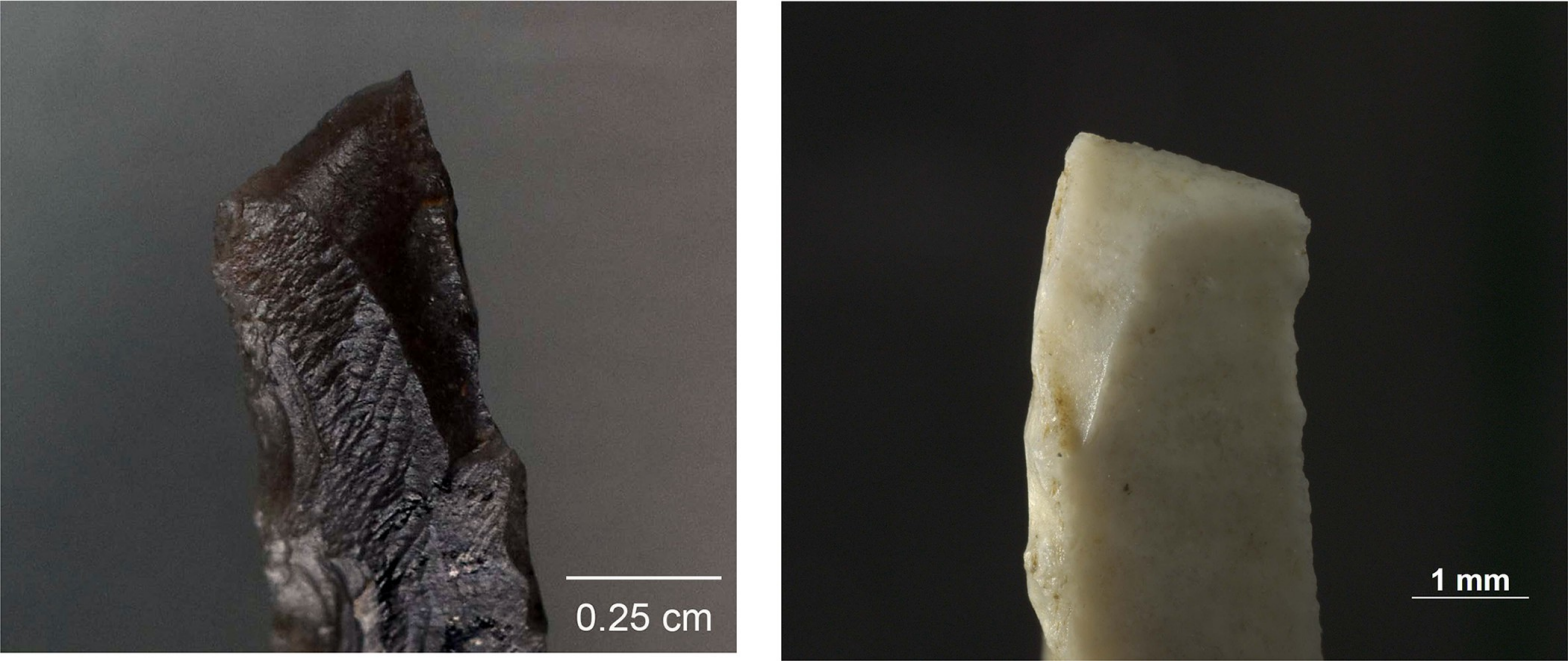
Two examples of oblique bending breaks from Level 2 and Level 3. a. AP/58-2-147; b. AP/58-3-994.

In addition to the often invasive lateral scarring, there are other features that seem to speak for the interpretation that laterally hafted armatures are well-represented in the Level 2 collection. Among them are bending-initiated breaks that start from a surface and have an oblique outline, removing the corner between the cutting edge and the extremity of the armature. It seems logical that this kind of a break would occur if the backed edge would be firmly glued to the shaft, allowing the bending forces to mostly act on the side of the cutting edge on impact. These features were documented both in the Level 2 and Level 3 samples, including nanogravettes belonging to the latter sample (Fig 20). This parallel, together with the relatively high frequency of lateral scarring at the expense of other forms of damage on the nanogravettes, is worth noting considering the assumption that nanogravettes would represent laterally hafted armatures.

One of the most characteristic features of the Level 3 microgravette sample is the presence of edge-to-edge bending breaks with an extremely long propagation (see Fig 12B and 12C above). Of the 30 bending breaks recorded for the microgravettes with strong evidence of impact, nine initiate on an edge, and three of them on the backed edge. This implies a situation where the backed (distal) edge of the armature was free to make contact with the target, which means that it was not protected by the shaft. It is possible that a bending break could initiate on the backed edge also on a laterally hafted insert as the result of a shock localised at the opposite corner (distal corner of the cutting edge) if the adhesive was resistant enough to prevent the insert from detaching in its entirety, but this kind of an event can be expected to be rarer than the formation of a similar break on a weapon tip.

**Fig 21:**
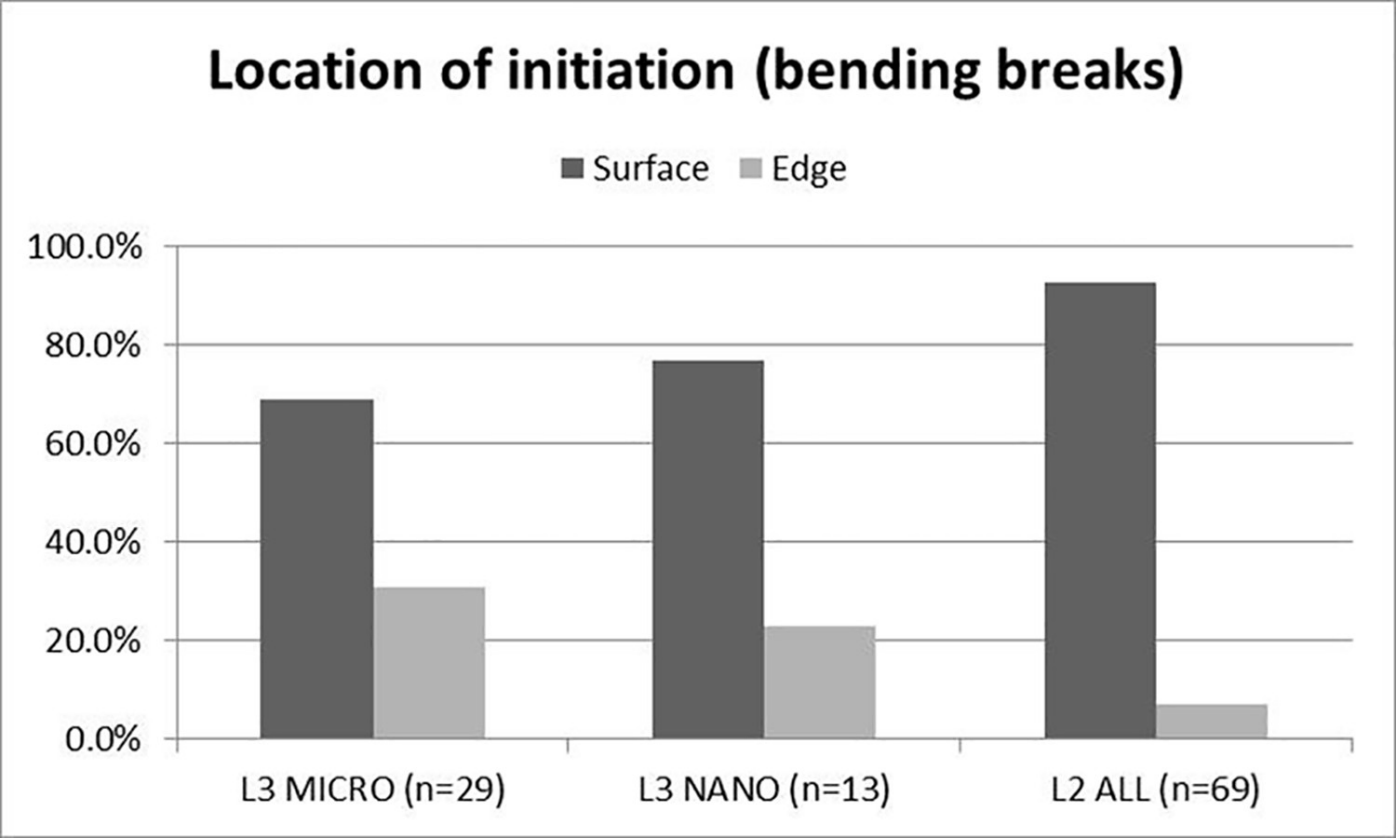
Location of bending break initiations in the Pataud Level 3 and 2 projectile samples for groups containing more than 10 observations. The breaks for which direction could not be determined are left out. Note the low number of observations for nanogravettes.

To examine whether this hypothesis would hold and whether the location of bending break initiation would be a criterion suitable for addressing the question of the position of the armatures in their shafts (distal vs lateral), the frequencies of different locations of initiation were calculated for all the bending breaks. These frequencies are shown in Table 12. Fig 21 compares the relative proportions of edge-to-edge and surface-to-surface breaks in categories where the number of observations exceeds 10 after excluding the cases for which the direction of the break could not be determined.

**Table 12:** Location of initiation for bending breaks in the Level 3 and 2 projectile samples.

Location of initiation	L3 Gravette	L3 micro-gravette	L3 nano-gravette	L3 truncated	L3 indet	L2
Ventral surface	6	7	3	1	3	30
Dorsal surface	0	13	7	1	4	34
Cutting edge	1	3	2	0	0	3
Backed edge	0	6	1	0	0	2
Indet	1	1	0	0	0	6
**Total**	**8**	**30**	**13**	**2**	**7**	**75**

The counts indicate numbers of features (as opposed to artefacts).

It is evident that the proportion of edge-to-edge breaks is very low in the Level 2 assemblage that is interpreted as including large quantities of laterally hafted elements and shows almost exclusively surface-to-surface breaks. The frequency is clearly higher among the Level 3 microgravettes, suggesting a difference in load conditions on impact between the two groups. However, as repeatedly pointed out above, the Level 2 assemblage probably shows a higher frequency of intentional breaks that can be expected to initiate on a surface simply because snapping a bladelet is easier in this direction. Therefore the dominance of surface-to-surface breaks may be partly due to factors other than hafting and use. Yet, if the frequency of pieces showing edge-to-edge breaks (which is perhaps a less biased measure of the commonness of this phenomenon) is examined, the contrast between the Level 3 microgravettes and the Level 2 sample remains clear. In the former, seven out of 18 pieces (or 38.9%) show this kind of a break [for similar frequencies on Gravette points, see 112], compared with only five out of 47 (or 10.6%) in the Level 2 sample. It would therefore appear that this variable is meaningful to look at in terms of weapon design.

The nanogravettes fall exactly in between the two other groups in terms of relative frequency of edge-to-edge breaks. While this may partly reflect the questionable choice of dividing the microgravette population into two size groups using an arbitrary limit (see above and S4 Appendix), it is obvious that these artefacts are very small-sized and fragile. They are therefore mostly difficult to perceive as effective weapon tips. Experimental testing involving different modes of propulsion and hafting systems would obviously be required to confirm this. At this stage, it can be mentioned that there is an explanation for the pattern seen in Fig 21 that does not invoke hafting of nanogravettes as tips. It is the possibility that the microgravette group contains a number of laterally hafted armatures, perhaps due to the arbitrary size limit used here. This would blur the difference between the nano and micro groups and give the impression of gradual change from one extreme (microgravettes) to the other (Level 2 inserts) in terms of frequency of lateral hafting.

#### 5.4.3 Composite point design

The discovery of laterally hafted inserts in both the collections studied here raises the question of the overall design of the assumed composite points. The regular, rectangular shape, and the back itself, of the Level 2 backed bladelets with impact damage gives reason to propose that they were hafted laterally, with the length of the back against the organic point. Similar configuration was suggested above for the nanogravettes with impact damage. The generally long and narrow shape of both the points in the Gravette family and the lateral inserts identified in the two collections speaks against the presence (or, at least, abundance) of true barbs (obliquely hafted elements on the lateral sides of a point) in the assemblage that was analysed here.

While the presence of lateral inserts, once hafted on organic points, is easy enough to demonstrate for Level 2, reconstructing the distal configuration of the composite points is more challenging. Certain previous studies have reported the co-existence of tips and lateral inserts (barbs) at the same site. Such is the case with the Final Palaeolithic site of Rekem, but there, barbs were heavily outnumbered by tips [120]. At least in the Level 2 collection, the relationship appears to be reverse. In their reconstruction of the Gravettian composite point from Les Prés de Laure, Tomasso et al. have proposed a design that involves a self-pointed osseous point equipped with lateral barbs [71]. This design was tested experimentally and succeeded in penetrating the target, suggesting that a lithic tip is not an obligation if the organic point is well sharpened. Others have reported similar results for spear tips made of antler, hard wood, and soft wood hardened by heat [112]. On the other hand, Gaillard et al. have found, using wooden points, that tipping the point with a microlith improves penetration considerably. They note that the size and morphology of the microlith are of limited significance as long as it is placed apically [124]. Pétillon et al. in turn have noted that on their reconstructions of self-pointed Magdalenian composite points, the morphology of the so-called head bladelet (the distalmost laterally hafted insert) is of crucial relevance for successfully piercing the hide. In their view, the head bladelet needs to show a curved cutting edge and pointed distal end to ensure that the point enters its target and the row of inserts located behind the head bladelet can serve its purpose [116].

**Fig 22:**
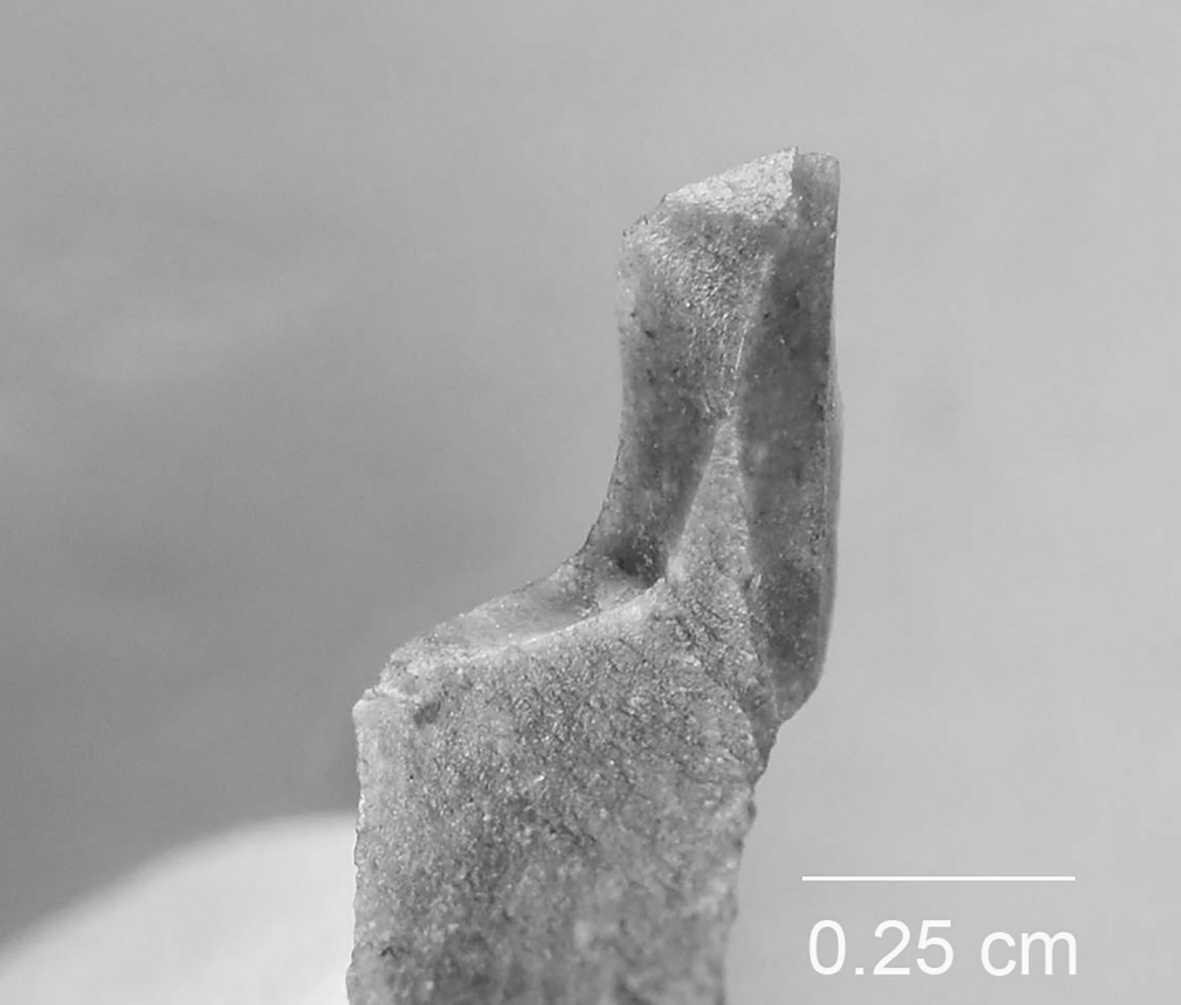
A break initiated and terminated on an edge on the partially backed bladelet AP/58-2-748 (Senonian flint).

Preliminary observations suggest that the possible presence of head bladelets in the Pataud Level 2 collection should be investigated. The Level 2 projectile sample has very few examples of edge-to-edge fracturing of the lithic armatures, but outside the sample of confidently identified projectiles, there is at least one artefact where the breakage somewhat resembles the most distinct edge-to-edge breaks in the Level 3 sample (see Fig 12 above). Given that this piece showed no characteristic wear apart from the pronounced edge-to-edge break (Fig 22), it could not be confidently identified as a projectile using the strict criteria applied here. Nevertheless, the presence of such a break that is virtually absent in the rest of the sample is noteworthy, particularly given that the piece is only partially backed. Theoretically, it could have been hafted at the distal extremity of a composite point, with its backed portion against the lateral edge of the outermost tip. Such a placement would explain the presence of an edge-to-edge break with a long propagation, a feature otherwise documented exclusively on the Level 3 microgravettes, which have been interpreted here as weapon tips.

**Fig 23:**
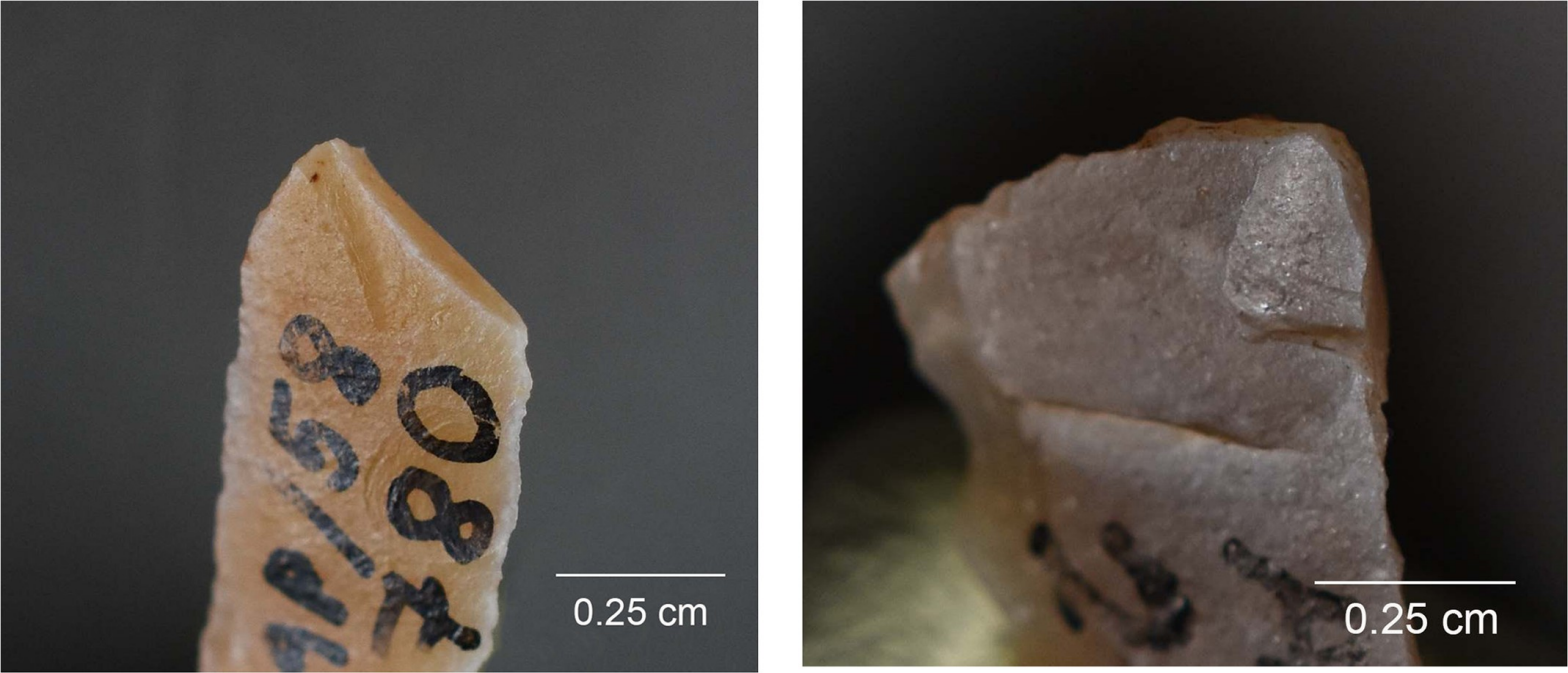
Removals linked to shaft contact or barb collision. a. An obliquely oriented elongated removal initiated on the backed edge on AP758-2-780 (Senonian flint). Note the large shallow scar on the opposite edge; b. A longitudinal removal initiated on the truncation on AP/63-2-1922 (Senonian flint). Note the preserved termination of a much larger, fissure-terminating removal that the smaller scar cuts and that is cut by the truncation.

While demonstrating that potential tips and lateral inserts were hafted together on the same weapon would undoubtedly be difficult, by examining damage that is caused by (detached) lateral inserts colliding on impact it may be possible to address the question whether they were hafted in rows. On a small number of Level 2 backed pieces (n = 6) confidently identified as projectiles, removals were recorded that were interpreted as having been caused by contact with either the shaft or another armature on impact. These initiate at one extremity of the backed bladelet and are oriented either parallel or oblique against its main axis (Fig 23). These removals are easiest to separate from other forms of damage when the extremity is truncated–in the case of armatures where both extremities show a snap break it is difficult to be certain that the scar does not represent a secondary removal detached at the moment of breakage. Part of the bidirectional scarring on armature cutting edges (see Fig 15 above and S7 Appendix) could also be explained by lateral inserts making contact not only with the target but also one another on impact. In cases where the scars are located on the side of the back, the damage could alternatively result from the insert being hafted in a groove, but it is also possible that extremely resistant hafting adhesive could produce similar counter-pressure removals. The severity of lateral scarring on many of the artefacts points to the use of strong glue (see S10 Appendix). The conditions under which such damage occurs should be investigated experimentally.

#### 5.4.4 Hafting of Gravette and microgravette points

Gravette and microgravette points are typically conceived as distally hafted armatures (weapon tips). This configuration has been tested experimentally with success and the resulting wear patterns have been found to match archaeological impact damage [see 14, 57, 58]. Alternative arrangements involving lateral hafting have been proposed [68, 69, 112], but archaeological evidence of such designs appears to be lacking. Hafting as tips is a plausible option for many of the Pataud Level 3 Gravettes and microgravettes given the overlap in wear patterns with the experimental collection available at TraceoLab [14, 114] and the experimentally produced features described in the published literature [e.g. 57]. As argued above, the relative frequency of edge-to-edge breaks on the Gravettes and microgravettes (Table 12, Fig 21) supports the distal hafting interpretation. The proportion of possible laterally hafted elements within the microgravette and nanogravette samples is currently unknown.

In the case of distally hafted armatures, previous experiments have tested hafting arrangements where the point is hafted axially or slightly obliquely to the axis of the shaft [57, 112]. An attempt was made here to distinguish between these two options in the Abri Pataud sample using two criteria: the frequency of edge-to-edge breaks and the orientation of lateral scarring.

The comparison between the archaeological sample and the available experimental data on microgravettes [14] in terms of the first variable yielded inconclusive results. The proportion of edge-to-edge breaks of all breaks is higher in the Pataud sample (27%) than in the experimental sample of axially hafted microgravette points (18%) and obliquely hafted ones (12%) (J. Coppe, personal communication). While the reason for this should be investigated through further analysis, it is possible that at least morphological differences play a role here as the experimental sample is not an exact match to the Pataud sample. Further, the experimental data used here combined samples of points that tipped spearthrower darts, arrows, hand-cast spears and thrusting spears. It is possible that the Pataud sample represents only some of these modes of propulsion, which may affect the results.

The second criterion, orientation of scarring on the cutting edge, produced clearer results. The experimental microgravettes hafted obliquely showed an exceptionally high proportion of lateral scars oriented obliquely to the cutting edge (9 out of 10 occurrences), whereas on axially hafted armatures, the proportion was close to 50% [14]. The Pataud microgravette sample (30 recorded occurrences of lateral scarring) presented an exact 50%-50% division between obliquely and perpendicularly oriented lateral scars or patches of scars. This would point towards axial rather than oblique hafting of the Pataud microgravettes. This hypothesis should, however, be tested through experiments that replicate the dimensions and morphological features of the Pataud artefacts more faithfully than the present experimental sample that was produced in the context of other studies.

#### 5.4.5 Question of other uses

Knife use has been proposed for some of the Gravette points from Level 5 at Pataud [61], and previous studies have argued that Magdalenian backed bladelets have been hafted not only as projectiles but also as knives [e.g. 117]. Experiments testing hafted backed bladelets in non-projectile use have found that these small tools can be used effectively and for long periods of time in craft activities, but that there are also certain limitations posed by their small size with respect to the handle [125, 126]. The sampling strategy here was heavily oriented towards documenting projectile damage, and pieces with minor edge damage that could be indicative of knife use were underrepresented in the samples. In addition, surface preservation, particularly in the Level 3 sample, led to the conservative interpretation of the observed wear traces as it became evident that some of the alterations (edge rounding, polish, striations) could mimic use-wear. While surface alterations and the probable overlap between certain forms of knife and projectile wear [cf. 117] limited interpretative possibilities here, some indications of non-projectile use could be found in both Level 3 and Level 2 samples.

In Level 3, the artefacts bearing potential evidence included truncated backed pieces (n = 3), a Gravette point, a microgravette, nanogravettes (n = 2), and a partially backed piece. The evidence consists of scarring, light edge rounding, and polish that varies somewhat in qualities from piece to piece. In the majority of cases, the wear could fit with knife use but projectile use could not be ruled out with certainty. A single nanogravette showed edge rounding at its distal extremity that could derive from its use as an awl. In all cases, the interpretation remains tentative at best.

**Fig 24:**
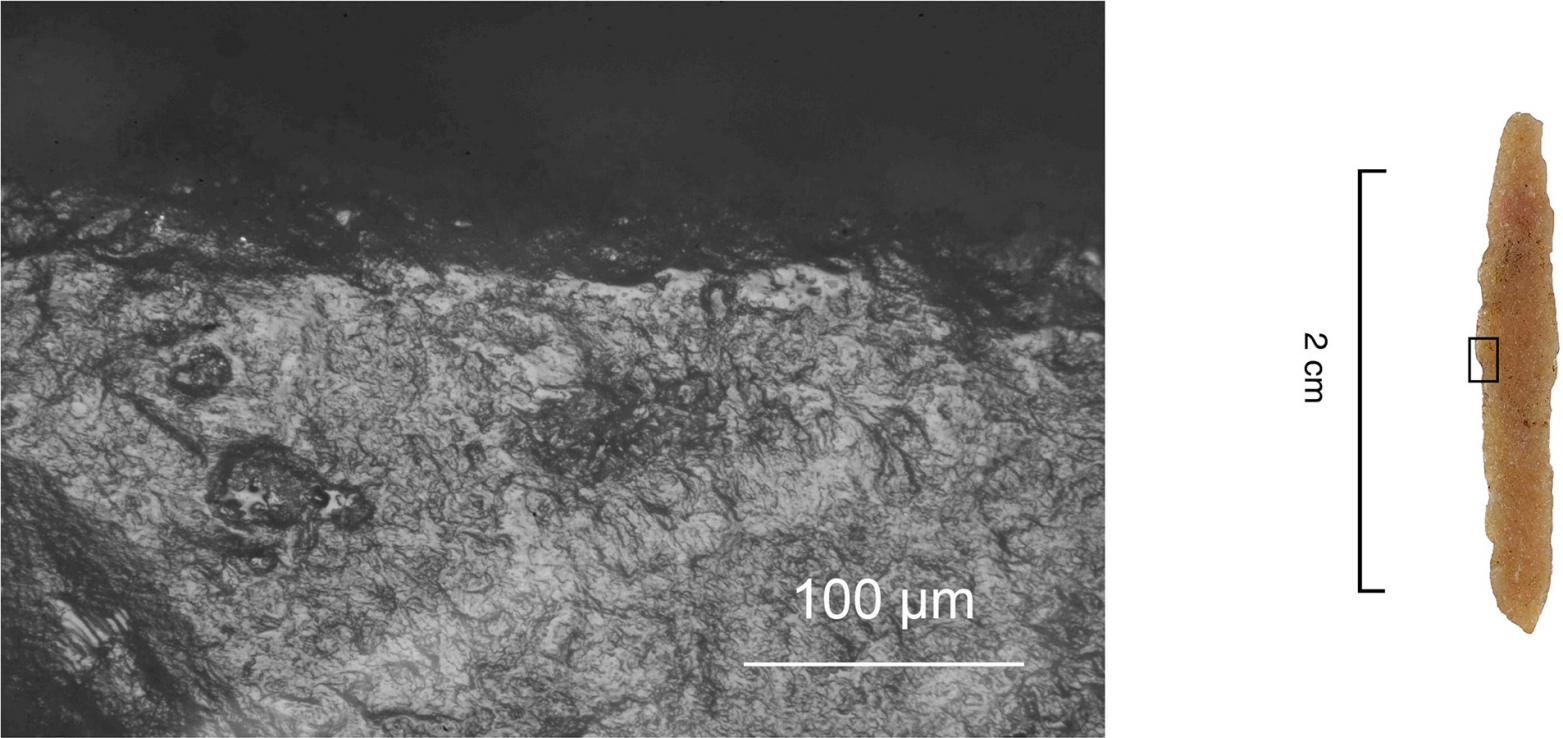
Well-developed longitudinal polish on AP/63-2-2018 (400×).

The number of Level 2 backed pieces analysed with high magnification for possible knife wear was very limited (n = 14). Out of these, two artefacts showed highest potential for such wear. While it is not impossible that the bending-initiated scarring and limited microscopic wear on one of them has got to do with projectile use, the other piece showed longitudinal polish that is more extensive than what is typically seen on armatures (Fig 24).

These observations demonstrate that the assemblage should be examined in further detail for the possible presence of composite knife inserts and other tools. Future experiments aimed at replicating the wear patterns observed on the backed tools should therefore preferably combine projectile tests with the use of backed artefacts in craft activities. It is relevant to note in this respect that knife inserts may have considerably longer use-lives than projectile armatures, and that they may consequently be underrepresented in archaeological assemblages if the examination is limited to absolute numbers of tools.

## 6. Discussion

### 6.1 Methodological considerations

The functional analysis of backed tools from Pataud Level 3 and Level 2 occupations demonstrated that armature typology does not straightforwardly reflect weapon design. While the Level 2 backed elements had been viewed as composite point inserts in previous typo-technological studies [70, 101], the status of Level 3 backed artefacts was less clear in this respect. The present study could confirm that a number of truncated backed pieces within this assemblage were once hafted, similarly to the Level 2 artefacts, as side elements. Furthermore, the analysis of the fracture attribute data allowed proposing that lateral inserts were not restricted to this morphology in the Level 3 assemblage, but that at least some of the so-called nanogravettes likewise functioned as laterally hafted elements. This highlights the importance of carrying out detailed functional analyses prior to linking typological differences to broader technological or behavioural changes.

The experimental and archaeological data presented here offered further critical viewpoints to the use of the DIF (diagnostic impact fracture) method in projectile identification [see also 8, 11]. The experiment where backed bladelets were snapped deliberately to produce shorter segments created fractures that qualified as DIFs (e.g. long bending-initiated step-terminating breaks) and thereby added to the growing body of evidence of the overlap between impact-related and non-impact-related breaks [see also 112]. The archaeological results confirmed the importance of including lateral scarring as an analytical category in projectile studies. These features have previously been overlooked at the expense of "diagnostic" breaks [8]. Here, the reliance on combinations of features instead of individual breaks produced a high-quality dataset on securely identified projectiles that could subsequently be used for reconstructing armature hafting.

Our study also raised questions regarding the usefulness of measurement data in projectile identification. Two points can be made here. First, the experiment where backed bladelets were intentionally snapped by bending produced secondary scars ("spin-offs") that are sometimes longer than the lower limit proposed for secondary scars diagnostic of projectile use [107]. Second, when a broader range of features was examined, impact fracture length appeared to be determined more by armature morphology and the location of fracture termination than by the dimensions of the armature, which undermines the significance of establishing size limits for "diagnostic" impact fractures based on armature size as has been proposed in some earlier studies [107, 110, 111, 127, 128]. While both these perspectives call for a reappraisal of the criteria currently used in projectile analysis, the second one also holds potential for future work. If fracture length is not strictly dependent on armature dimensions, it must be controlled by other factors, among which some may be relevant for reconstructing prehistoric weaponry. If, for instance, the effect of morphology and the strength of the hafting system that were judged significant here could be controlled for, the comparison of fracture dimensions could potentially reveal something about the loading conditions at the moment of impact, and metrical data could aid in investigating weapon ballistics [cf. 14, 129, 130].

Based on these insights and in view of future studies, we advocate an approach that relies on impact trace patterning (combinations of features) and on detailed recording of impact fracture attributes and frequencies [8, 11, 14, 106], and uses these data as a starting point in attempts to address higher-level questions related to prehistoric weaponry. While the method we used here in a modified form is currently in the process of being formalised and tested with larger datasets [8, 14, 71, 104], the results presented here show that already simple treatment of attribute data can contribute to the reconstruction of weapon design by, for instance, helping distinguish between distally and laterally hafted armatures.

### 6.2 Weapon design and hunting strategies in the Recent and Final Gravettian at Abri Pataud

The functional data on lithic armatures from Level 3 and Level 2 at Pataud indicate that the Recent Gravettian assemblage represents both distally hafted weapon tips (Gravette and microgravette points) and lateral inserts (at least truncated backed pieces and nanogravettes) that once belonged to composite points. The Final Gravettian collection in turn consists dominantly, if not exclusively, of laterally hafted armatures, i.e. composite point inserts. The sixteen Level 3 artefacts with the strongest evidence of lateral hafting, namely truncated backed pieces with various signs of impact (n = 12) and nanogravettes with heavy lateral scarring often associated with minor breaks (n = 4) come from the upper and middle parts of the sequence, that is, from Lens 1 (n = 1), *Eboulis* b (n = 5), Lens 2 (n = 6), Lens 2 Main (n = 3), and Lens 2b (n = 1). These translate into *ensembles 1* to *3* following the grouping of stratigraphic units favoured by recent studies [33, 87, 91]. None of the strongest candidates for laterally hafted armatures identified in the present study derive from the oldest Level 3 occupations (Lenses 3 and 4/*ensemble 4*). While this could be taken as an indication of chronological developments within Level 3, it needs to be kept in mind that the backed piece assemblage from *ensemble 4* is limited in size [cf. 87] and would require further study prior to arguing for chronological trends at a finer resolution. For now, a degree of continuity can be suggested in weapon manufacture traditions from Level 3 to Level 2. Nevertheless, the contrast in the relative importance of the composite point between these two main levels is of significance and needs to be explained from a hunting behavioural point of view.

A projectile weapon head serves two purposes: it cuts a hole in the hide of the prey animal large enough to allow the shaft to pass with as little friction as possible, and inflicts lethal damage e.g. by puncturing a vital organ or causing severe haemorrhage [116, 131]. Generally, points that are acute-angled in plan view are required to penetrate the skin of the animal, but very long and narrow tips may not be resistant enough against skin or bone and can therefore fracture prematurely (i.e. before inflicting a large enough wound). On the other hand, sturdier points that could more easily break or pass a rib or puncture a shoulder blade are more likely to bounce off the skin of the prey, particularly if hitting it diagonally [131]. Composite points have been found to perform better than organic points thanks to the increased cutting ability provided by the lithic inserts [116]. The ideal qualities of the weapon further depend on whether it is supposed to immobilise (e.g. dart or arrow) or dispatch the prey (e.g. thrusting spear), or both [131].

Prey choice might affect point size and design because the larger the hunted animal, the larger (deeper) the wound needs to be to immediately disable the prey [131]. Opting for larger game therefore requires weapon tips with better cutting and penetration capacities. This could potentially explain the shift from microgravette points to composite points at Pataud, where the analysis of the faunal remains from the recent excavations has demonstrated that large bovids were exploited on the side of reindeer [34]. In the newly excavated material, animals larger than reindeer are better represented than in the previous data on the Movius collection (Table 13). Also mammoth is present in the Level 2 record in the form of worked ivory and a single femur fragment deriving from a young individual [33, 34], but while mammoth tusks were abundantly used for the manufacture of ornaments at Pataud during the Final Gravettian, this species does not appear to have contributed significantly to the human diet judging from the bone remains [132]. However, recently measured isotope ratios on human and animal bones from Pataud and nearby contemporary sites suggest that reindeer may not have dominated the diet of the occupants of Pataud as heavily as the skeletal remains would indicate. If the isotope signature were to be explained by the consumption of other terrestrial mammals, horse and mammoth would be likely candidates, although other alternatives such as the potential contribution of freshwater fish to the diet remain to be investigated [132].

**Table 13:** Representation of terrestrial mammals in the faunal material from Pataud according to recent zooarchaeological studies [33, 34].

Species	Level 3 (Cho, 1998)				Level 2 Movius collection (Cho, 1998)				Level 2 recent excavations (Crépin, 2013)			
	NRDt	*%*	MNIc	*%*	NRDt	*%*	MNIc	*%*	NRDt	*%*	MNIc	*%*
*Mammuthus primigenius*	0	*0*.*0*	0	*0*.*0*	2	*0*.*0*	2	*2*.*7*	4	*1*.*0*	1	*5*.*9*
*Coelodonta antiquitatis*	1	*0*.*0*	1	*0*.*7*	0	*0*.*0*	0	*0*.*0*	0	*0*.*0*	0	*0*.*0*
*Equus sp*.	194	*2*.*1*	11	*7*.*1*	54	*1*.*6*	3	*4*.*1*	13	*3*.*4*	1	*5*.*9*
*Bos sp*. */ Bison sp*.	144	*1*.*5*	10	*6*.*5*	23	*0*.*7*	1	*1*.*4*	44	*11*.*5*	3	*17*.*6*
*Rangifer tarandus*	8767	*93*.*9*	101	*65*.*2*	2894	*87*.*6*	54	*73*.*0*	291	*75*.*8*	6	*35*.*3*
*Cervus elaphus*	100	*1*.*1*	8	*5*.*2*	51	*1*.*5*	4	*5*.*4*	14	*3*.*6*	1	*5*.*9*
*Capra ibex*	66	*0*.*7*	11	*7*.*1*	66	*2*.*0*	2	*2*.*7*	3	*0*.*8*	1	*5*.*9*
*Rupicapra rupicapra*	46	*0*.*5*	7	*4*.*5*	165	*5*.*0*	5	*6*.*8*	2	*0*.*5*	1	*5*.*9*
*Sus scrofa*	3	*0*.*0*	2	*1*.*3*	0	*0*.*0*	0	*0*.*0*	0	*0*.*0*	0	*0*.*0*
*Canis lupus*	2	*0*.*0*	1	*0*.*7*	42	*1*.*3*	2	*2*.*7*	10	*2*.*6*	1	*5*.*9*
*Vulpes vulpes*	14	*0*.*2*	3	*1*.*9*	8	*0*.*2*	1	*1*.*4*	1	*0*.*3*	1	*5*.*9*
*Lepus sp*.	0	*0*.*0*	0	*0*.*0*	0	*0*.*0*	0	*0*.*0*	2	*0*.*5*	1	*5*.*9*
**Total**	**9337**	***100*.*0***	**155**	***100*.*0***	**3305**	***100*.*0***	**74**	***100*.*0***	**384**	***100*.*0***	**17**	***100*.*0***

Determining how the potential shift to larger prey in Level 2 might have affected point design is not straightforward using currently available archaeological data. The recently recovered composite point from Les Prés de Laure (France), dated to late Gravettian, was found in association with horse remains [71]. This species is present at Pataud in Level 3 as well as Level 2 (Table 13), and does therefore not by itself justify the dominance of composite points in Level 2. Similarly to Pataud Level 3 that is here viewed as testifying to the co-occurrence of (at least) two alternative weapon designs, the Prés de Laure assemblage has been interpreted as representing two parallel designs, i.e. barbed composite points and distally hafted flint tips [71]. Direct evidence of mammoth hunting in the Gravettian comes from the site of Kraków Spadzista (Poland) where a fragment of a flint point has been found embedded in a mammoth rib [44]. The dimensions of the shouldered points from which the tip fragment is suspected to derive are equal to or larger than the biggest Gravette points known from Level 3 [44 and S1 Appendix], which could suggest that lithic tips larger than the ones used at Pataud in Level 3 would be preferable for hunting such large prey, and the composite point could have been a response to such size demands.

Crucially, however, the effectiveness of a hunting weapon is determined not only by the size of its tip but is also affected by tip morphology, the qualities of the shaft (e.g. weight, perimeter) [63, 133] and, most importantly, the weapon system used [e.g. 14, 63, 129, 131]. Different weapon systems produce different amounts of kinetic energy [129], which means that point morphometrics can and need to vary according to weapon parameters and conditions of use. In consequence, choice of point design does not simply depend on the size of the prey but is dynamically linked to overall weapon design as well as the hunting situation (e.g. distance to the target, number of hunters involved). A method for identifying weapon systems (propulsion modes) based on impact fracture characteristics is currently in development for distally hafted armatures [14, 104], but at the moment no reliable method exists that would allow inferring the mode from damaged laterally hafted inserts. Until this information becomes available, the link between prey size and weapon tip characteristics cannot be fully addressed.

Even though other (larger) species may have been exploited by the occupants of Pataud to a significant extent [132], the faunal assemblage from the site itself testifies to frequent and skilled hunting and butchering of reindeer during both Level 3 and Level 2 occupations [33, 34]. To explain the observed shift in lithic armature technology, it is therefore relevant to examine whether something changed in the targeting of this particular game species. Recent analyses of the fauna from Level 3 and 2 at Pataud have demonstrated that despite their shared emphasis on reindeer (Table 13 above), they differ from each other in terms of the composition of the animal population. Level 3 shows a catastrophic mortality profile and Level 2 an emphasis on individuals that were easy to kill (old and young individuals and pregnant females) [33]. A subsequent study by Crépin has linked this difference to seasonality, suggesting that a catastrophic profile is consistent with hunts during a period when the reindeer aggregate into large herds, whereas the selective hunting of vulnerable individuals would be more consistent with a winter situation when reindeer live in separate, smaller groups. He further notes that hunts during migration season require larger groups of humans whereas in the winter, smaller groups are sufficient [34].

In this scenario, the Level 3 assemblage could represent a situation where the hunt was based on a power-in-numbers approach, meaning that a large enough group of people were involved to guarantee the success of the operation, and the performance of an individual weapon was not as crucial given that there were other hunters for backup, therefore allowing the use of projectiles that required less time investment and were perhaps less robust in design. The Level 2 assemblage, on the other hand, could represent a context where more relied on the performance both of an individual hunter and of an individual weapon due to a more limited number of both hunters and prey involved. In this respect, the capacity of the composite point to inflict larger, deeper wounds [116] could have offered the hunters an advantage.

The choice of composite points in winter hunting contexts could be further argued for on the basis of the effect of cold conditions on projectile point performance and perhaps also animal physiology. Colder, harsher conditions, as documented in the Level 2 record, probably signified both lower winter temperatures and longer winters. Lithic projectiles have been argued to become more brittle as the temperature drops [133, 134], which might increase the risk of failure of flint-tipped projectiles, and again encourage composite designs instead. More speculatively, targeting animals bearing long and dense winter fur might also encourage the use of robust points. The effect of coat of hair has been mentioned as potentially relevant for projectile performance by some investigators [135] whereas others have seen it as of little importance unless the target is hit at a clearly oblique angle [136]. Frison’s comment concerned Clovis points, which can be considered sturdier than the Gravettes and particularly microgravettes in the Pataud Level 3 assemblage, and further data would be needed to evaluate the effect of this variable in the particular case of Pataud.

All these aspects put together, the technological change from Level 3 to Level 2 can be explained by a shift in hunting tactics, linked to seasonality and social organisation, and/or by new demands posed on the weaponry by the altered environmental conditions and possible shifts in prey choice [for similar reasoning, see 112]. Ideally, the link between faunal remains and different reconstructed weapon designs should be investigated at a finer scale than was possible in the present study that grouped together the different occupation phases within Level 3 and Level 2. Such an approach could shed further light on the choices made by the hunters over the tens of thousands of years the Pataud record covers.

Existence of composite points has been frequently proposed for the Aurignacian on the basis of the abundancy of recovered standardised microlithic elements [e.g. 137, 138]. Further, a number of studies relying on variations of the DIF approach (see above) have reported macrofractures indicative of projectile use on Protoaurignacian or Aurignacian bladelets [110, 123, 128, 139–141], sometimes also illustrating or describing combinations of features that are indeed highly indicative of projectile use [e.g. 110, 128] or mentioning microwear traces as a supportive argument [123]. Replicative experiments, however, have so far either been lacking or have not been combined with use-wear analysis [142], and use-wear studies rarely describe the observed features and their frequencies in sufficient detail to allow a critical evaluation. While projectile function remains to be confirmed for these bladelets through further studies, lateral hafting has already been argued for them on the basis of trace patterning, e.g. the location of use-wear opposite to the retouched edge [123], presence of lateral scarring [140], orientation and position of lateral scarring [128], or morphological criteria in combination with the direction of laterally initiated breaks [110]. These claims remain to be verified as also often noted by the authors themselves. It is evident that the mere presence of lateral scarring is not proof of lateral hafting, as this kind of damage occurs abundantly also on distally hafted experimental armatures of varying morphologies [14], and all in all, establishing that (some) Protoaurignacian and Aurignacian bladelets made part of composite points would require further experimental testing and data analysis. In sum, functional studies employing strict projectile identification criteria relying on combinations of macroscopic and microscopic features comparable to the present study are currently lacking for the Aurignacian.

In Gravettian contexts, lateral hafting of microgravettes in a row has been argued for the site of La Vigne Brun, but the proposed evidence–removals resulting from tangential impact [69]–is not shown and is not convincing argument by itself considering that also distally hafted armatures can make tangential contact with the target. Neither is ’tangential impact’ proof of serial hafting of backed elements. Lateral hafting has likewise been proposed for backed tools from the Italian early Gravettian [143, 144] but this claim relies on morphological observations and fragmentation patterns and detailed use-wear data are not provided. The study also suffers from methodological setbacks linked to fracture identification and description [see 8]. At present, the oldest partially preserved composite point that use-wear analysis has confirmed as a hunting weapon comes from the late Gravettian occupation at Les Prés de Laure, south-eastern France, dated to around 20,000 BP [71]. Considerably older, compelling evidence of hunting weapons that incorporated lithic and ivory elements is present at the Siberian site of Yana, dated to between 29,000 and 27,000 BP. Here, lithic armature fragments and an ivory shaft splinter were found embedded in the same hunting lesion in a mammoth scapula, but the exact design of the weapon cannot be determined and the authors have proposed it involved a lithic tip and a foreshaft [145]. This weapon was therefore probably not comparable in design to those reconstructed previously for Les Prés de Laure [71] and now for Abri Pataud. Impact damage has been reported on microliths from an LGM site in Moravia, Czech Republic, interpreted as composite point elements. This study considered both DIFs and oblique lateral scarring but the argument for composite points relies on artefact size and morphology alone [109] and therefore remains to be verified with functional data.

In Europe, organic points with lateral inserts or barbs become more frequently known in post-LGM contexts [see 71, 118, 146–148], although detailed functional studies remain scarce. An often-cited example is the antler composite point find from Pincevent [149], a site where the focus was, similarly to Pataud, on reindeer hunting. Further east, the well-preserved find from Talicki (also mentioned as Talickij, Talitski, or Ostrovskaya), located at the base of the Ural mountains, has also been often depicted [see 146, 148] and is attributed to the Late Glacial/final Upper Palaeolithic [146, 150].

Notably, the evidence of composite points, as reported in recent studies [71, 118], becomes more abundant when the trend in Europe is towards warmer, not colder, climate [e.g. 96]. This means that on the large scale, the success of the composite point cannot be explained solely by its functionality in arctic or subarctic conditions. To add to this, a recent study has found indications of the use of small quartz elements as barbs on hunting weapons in the Howiesons Poort of Sibudu Cave, South Africa, and while detailed functional studies on African assemblages remain few in number, small backed lithics are known from various chronological and environmental contexts within the continent [151]. This means that composite tool designs have emerged and persisted under diverse conditions, and that understanding the motives for opting for composite tools requires detailed contextual data.

The Pataud case study shows that there is continuity in technical choices from the Recent Gravettian to the Final Gravettian in the sense that composite projectile points are present already in the Level 3 record. Consequently, the Final Gravettian armature assemblage, instead of signalling the introduction of a wholly new weapon design, reflects the complete or near complete abandoning of distally hafted weapon tips in favour of composite points. The possible explanations offered here for this shift show that it can potentially be explained without having to assume that the Recent Gravettian and Final Gravettian remains represent cultural discontinuity. Instead, the differences in chosen weapon designs could have to do with particular hunting strategies that are linked to seasonality and the organisation of subsistence activities. To fully understand the changes and their significance, other kinds of data on e.g. domestic tool technology, lithic raw material economy, subsistence base, mobility patterns, and demography should preferably be incorporated in the discussion.

## 7. Conclusions

Our detailed use-wear analysis of the backed tools from Movius’s excavations at Abri Pataud allowed establishing the presence of composite points in both Level 3 (c. 24,000 BP) and Level 2 (c. 22,000 BP) assemblages. On the side of this design, weapon tips (Gravette and microgravette points) that were probably hafted axially rather than obliquely are abundantly present in the Level 3 material, but are either marginal or entirely lacking in Level 2. Whether the Level 3 collection represents adoption of composite point designs over time remains an open question until more data becomes available for the time frame represented by the oldest occupations. Even if Level 3 would show a chronological trend, composite points only become dominant in Level 2. Factors that may have encouraged the use of such points during this younger settlement phase include winter hunting conditions where individual reindeer were targeted, potentially by a limited number of hunters [34], which may have put more pressure on the performance of an individual hunter and a weapon as opposed to hunting by large groups of people during migration season. Cold winter conditions may also have favoured the use of partly organic composite points over lithic ones. Further, the recent faunal and isotope data indicate that the subsistence strategies of the Final Gravettian hunters may have incorporated a larger proportion of herbivores above the size of reindeer. Encountering such large prey may equally have stimulated the use of composite points equipped with lithic cutting edges. To further evaluate the validity of these hypotheses, data on the weapon system(s) used by the Pataud hunters would in the first place be required and additional data on the overall subsistence base and mobility patterns as well as lithic raw material economy should be considered.

On a methodological level, our study highlighted the importance of using strict criteria relying on combinations of impact features in identifying archaeological projectiles. It also demonstrated the value of detailed break and scar attribute recording for the identification of projectiles and the reconstruction of armature hafting systems. In addition, our experimental and archaeological data made it clear that caution is needed in using impact fracture metrics for functional inferences by showing that the previously suggested limits for secondary scarring "diagnostic" of impact are not reliable and that impact fracture length appears to be more strongly controlled by armature morphology and location of fracture termination than the overall armature size.

Put together, these data illustrate the potential of detailed microwear analysis of projectile armatures for addressing questions linked to past human adaptations. When combined with faunal and climatic data, such analysis is able to bridge human technologies and past environments and can also aid in reconstructing social organisation that is often difficult to address directly using archaeological data from the more distant periods.

## Supporting information

S1 Appendix Classification criteria for Level 2 preliminary analysis sample. Description of categories used in preliminary functional screening.
(PDF)

S2 Appendix Data recording during low magnification analysis. Attributes recorded for each fracture on potential projectiles and morpho-technological attributes recorded for the analysed artefacts.
(PDF)

S3 Appendix Possible production-related scars in Level 2 sample. Illustration of a potential production-related scar recurrent in Level 2 sample.
(PDF)

S4 Appendix Measurement data for Level 3 backed points. Artefact width and thickness for the Gravette, microgravette and nanogravette sample.
(PDF)

S5 Appendix Impact fracture length in Level 3 sample. Length of bending break propagation and secondary scars plotted against artefact width.
(PDF)

S6 Appendix Damage on two Level 2 denticulated backed pieces. Description of potential impact-related damage.
(PDF)

S7 Appendix Length of armature use. Evidence of reworking of damaged armatures.
(PDF)

S8 Appendix Impact fracture frequencies in the Level 2 sample. Comparison of relative frequencies of main impact fracture categories in the Level 2 subsamples and complete sample.
(PDF)

S9 Appendix Data for Fig 18. (PDF)

S10 Appendix Haft raw materials. Discussion of the strength of glue and other aspects of projectile hafting arrangements based on archaeological impact damage intensity and experimental observations.
(PDF)
