# Supplementary material for: Why did hunting weapon design change at Abri Pataud? Lithic use-wear data on armature use and hafting around 24,000–22,000 BP
Source: PLoS One. 2022 Jan 14;17(1):e0262185. doi: 10.1371/journal.pone.0262185 (PMC8759672; doi:10.1371/journal.pone.0262185)
Supplement: S1 Appendix — Description of categories used in preliminary functional screening. (PDF) [file pone.0262185.s001.pdf]

Why did hunting weapon design change at Abri Pataud?

Noora Taipale, Laurent Chiotti, Veerle Rots

Supporting information

S1 Classification criteria for Level 2 preliminary analysis sample

The categories referred to in Table 4 (main text) were defined as follows:

1) *breaks suggestive of impact* (typically bending-initiated breaks with a long propagation and varied terminations, but included in this category are also breaks that do not seem to occur on pieces interpreted as production waste, e.g. oblique breaks initiating on a surface on the side of the thin edge as well as breaks initiating on a lateral edge; pieces in this category may show lateral edge damage (scarring) in addition to the break)
2) *substantial lateral edge damage* (typically large invasive removals on the cutting edge, usually with at least partly oblique orientation and abrupt and/or fissured terminations, consistent with rather violent contact with hard material, but also smaller scarring was included in this category as long as it looked non-random; at this stage, a distinction was not attempted between projectile and other uses although at its most typical, the heaviest kind of edge damage could only result from impact or heavy-duty butchery)
3) *minor edge damage* (i.e. small removals, typically of the type seen from contact with soft material on e.g. knives and projectiles)
4) *no edge damage / isolated minuscule scars* (i.e. scars that can easily be expected to form during production or as a result of low-energy taphonomic processes)
5) *production waste* (i.e. artefacts that are clear side products of the backing and snapping process, typically proximal or distal extremities displaying no use-wear and only partial backing [1: fig. 61], and
6) *indet* (fragments that are too small or badly preserved to interpret, as well as occasional artefacts that are not strictly speaking backed, such as ones showing other types of retouch, but that had been bagged together with the backed bladelets during previous studies).

Bibliography

1. Chiotti L, Nespoulet R, Morala A, Guillermin P. Données typo-technologiques de l'industrie lithique. In: Nespoulet R, Chiotti L, Henry-Gambier D, editors. Le Gravettien final de l'abri Pataud (Dordogne, France) : Fouilles et études 2005-2009 BAR International Series 2458. Oxford: Archaeopress; 2013. pp. 111–126.
