# Supplementary material for: Why did hunting weapon design change at Abri Pataud? Lithic use-wear data on armature use and hafting around 24,000–22,000 BP
Source: PLoS One. 2022 Jan 14;17(1):e0262185. doi: 10.1371/journal.pone.0262185 (PMC8759672; doi:10.1371/journal.pone.0262185)
Supplement: S2 Appendix — Attributes recorded for each fracture on potential projectiles and morpho-technological attributes recorded for the analysed artefacts. (PDF) [file pone.0262185.s002.pdf]

Why did hunting weapon design change at Abri Pataud?

Noora Taipale, Laurent Chiotti, Veerle Rots

Supporting information

S2 Data recording during low magnification analysis

The following attributes were recorded for each break and scar or patch of scars on the artefacts judged as potential projectiles as separate entries on an Excel sheet [for the development of the attribute system, see 1–3].

Table S2.1 *Attributes recorded for each fracture observed on potential armatures in the detailed low magnification sample from Abri Pataud.*

**Location**	proximal / medial / distal left lateral edge / …
**Type of initation**	bending / cone / intermediate / removed
**Location of initiation**	ventral surface / left lateral edge / earlier break surface / …
**General direction**	parallel / oblique / perpendicular to the direction of impact
**Length of propagation**	in mm, when relevant
**Type of termination**	snap / feather / hinge / step / complex (e.g. hinge-to-step) / removed
**Location of termination**	ventral surface / left lateral edge / …
**Fracture composition**	single / multiple
**Traditional terminology**	e.g. bending-initiated step-terminating break / spin-off / burination
**Fracture part**	positive / negative
**Fracture group**	scar / break
**Associated with**	(link to other database entries, i.e. other features on the piece)

The basic morphological and technological characteristics were recorded on a separate sheet using the following categories:

Table S2.2 *Attributes recorded for each backed artefact.*

**Raw material**	Senonian / Bergerac / Fumelois / …
**Blank type**	Bladelet / burin spall / indet / …
**Cortex**	0% / <50% / >50% / 100%
**Artefact type**	Gravette / indet backed piece
**Size group (for Gravettes and microgravettes)**	Gravette / microgravette / nanogravette
**State**	Finished / preform / production discard
**Fragment type (intact/prox/med/dist)**	Intact / proximal / medial /distal
**Cross-section**	Triangular / trapezoidal / …
**Profile curvature**	Curved / slightly curved / not curved (+ location, e.g. distal)
**Twistedness**	Twisted / slightly twisted / not twisted (usually proximal portion)
**Edge coverage of backing**	0% / <50% / >50% / 100%
**Backing type**	Direct / indirect / crossed

**Backed edge**	Left / right
**Location of tip**	Distal / proximal
**Additional retouch location**	E.g. proximal left edge
**Additional retouch type**	E.g. indirect semiabrupt
**Butt preserved**	Yes / no
**Max nr of parallel (original) dorsal ridges visible**	0 / 1 / 2 / …
**Max length**	in mm
**Max width**	in mm
**Max thickness**	in mm
**Screening category**	Impact / backing / production / heat / indet / …

Bibliography

1. Hayden B. Lithic Use-Wear Analysis. New York: Academic Press; 1979.
2. Coppe J, Rots V. Focus on the target. The importance of a transparent fracture terminology for understanding projectile points and projecting modes. J Archaeol Sci Reports. 2017;12: 109–123. doi:10.1016/j.jasrep.2017.01.010
3. Coppe J. Sur les traces de l’armement préhistorique : mise au point d’une méthode pour reconstruire les modes d’emmanchement et de propulsion des armatures lithiques par une approche expérimentale, mécanique et balistique. Université de Liège. 2020.
