# Supplementary material for: Why did hunting weapon design change at Abri Pataud? Lithic use-wear data on armature use and hafting around 24,000–22,000 BP
Source: PLoS One. 2022 Jan 14;17(1):e0262185. doi: 10.1371/journal.pone.0262185 (PMC8759672; doi:10.1371/journal.pone.0262185)
Supplement: S3 Appendix — Illustration of a potential production-related scar recurrent in Level 2 sample. (PDF) [file pone.0262185.s003.pdf]

Why did hunting weapon design change at Abri Pataud?

Noora Taipale, Laurent Chiotti, Veerle Rots

Supporting information

S3 Possible production-related scars in Level 2 sample

Scars similar to the one depicted below were observed on a single experimental piece that was snapped intentionally and on several archaeological artefacts (Fig S3). While further experimentation is needed to determine whether they can result from several processes, including tool use, it is evident that such scars can form when backed bladelets are snapped deliberately.

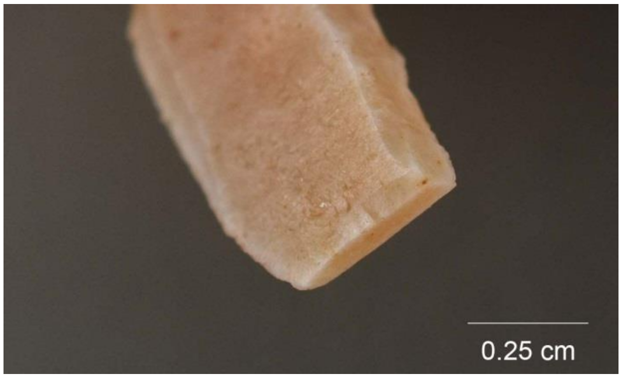

Fig S3 *An elongated removal initiated on the backed edge on AP/58-2-780.*
