# Supplementary material for: Why did hunting weapon design change at Abri Pataud? Lithic use-wear data on armature use and hafting around 24,000–22,000 BP
Source: PLoS One. 2022 Jan 14;17(1):e0262185. doi: 10.1371/journal.pone.0262185 (PMC8759672; doi:10.1371/journal.pone.0262185)
Supplement: S4 Appendix — Artefact width and thickness for the Gravette, microgravette and nanogravette sample. (PDF) [file pone.0262185.s004.pdf]

Why did hunting weapon design change at Abri Pataud?

Noora Taipale, Laurent Chiotti, Veerle Rots

Supporting information

S4 Measurement data for Level 3 backed points

An earlier technological study on the Level 3 collection has argued that the width of Gravette points centres around 6 or 7mm whereas microgravettes cluster around 4mm, but in this analysis, the widths were presented as relative proportions for predetermined categories [1].

Here, the measurements were plotted without classifying the material beforehand to investigate whether the low magnification sample (n=113) shows some form of clustering. Only width and thickness values were considered, as the dominance of fragmentary pieces means that length measurements are of little informative value.

Clustering is vague at best (Fig S4.1). Width and thickness values show more or less a continuum, and separation between Gravette and microgravette points is hindered by observations becoming scarcer with increasing width and thickness. In other words, "large" points are so rare in the sample that the slight discontinuity seen in the graph around the width of 9mm may well be just the effect of diminishing sample size.

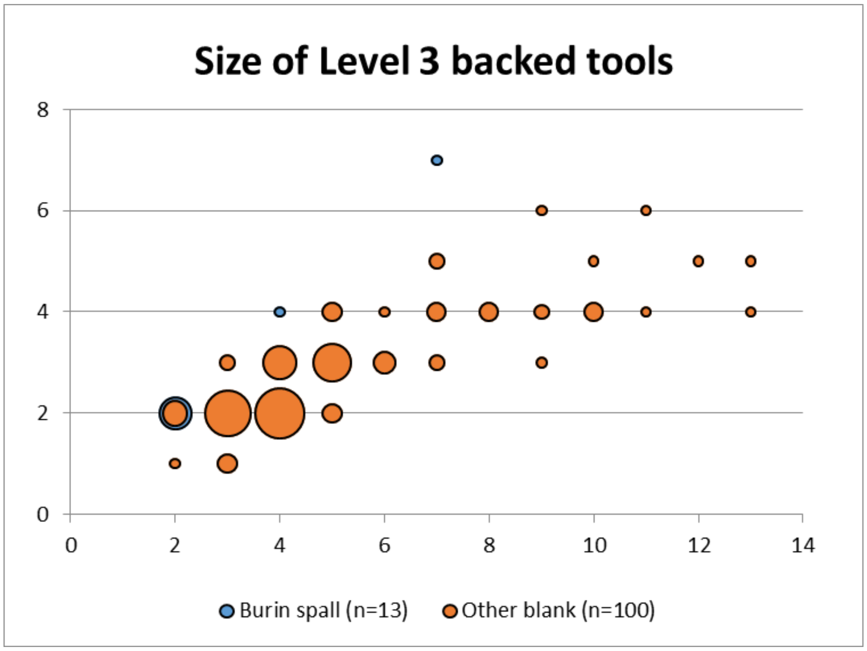

Fig S4.1 *Width (horizontal axis) and thickness (vertical axis) measurements in the Level 3 backed tool sample from Abri Pataud. The artefacts made on obvious burin spalls are plotted separately. Unfinished and potentially unfinished products are excluded, as well as fragments shorter than 1cm. The values are discontinuous because the artefacts were measured at 1mm accuracy.*

The clearest outlier in the current sample in terms of dimensions is a point measuring 7mm in both width and thickness, made on a large burin spall (Fig S4.2). It becomes classified here as a microgravette because of its unusually low width/thickness ratio despite its considerable length (83mm) that exceeds that of the largest fragments recorded. This example illustrates the difficulty of establishing analytically relevant size groups for a population of backed tools made on varied blanks and showing a high rate of fragmentation. Nevertheless, the rest of the group measured here shows fairly consistent width/thickness ratios, and the grouping employed here will do for making preliminary observations on potential differences in use and hafting of backed tools of various sizes.

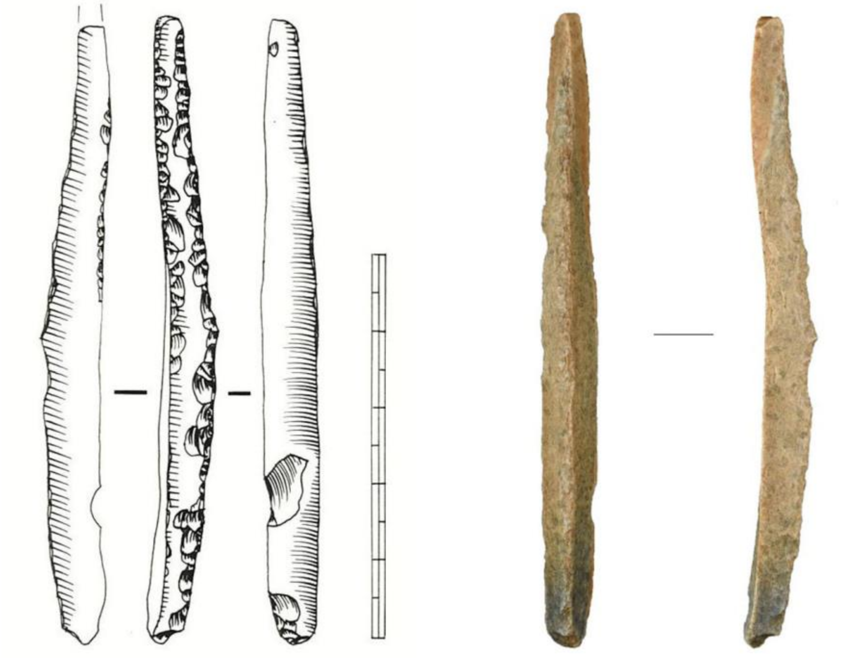

Fig S4.2 *Gravette point AP/58-3-1118, made on a large burin spall. Drawing M. Dauvois.*

Bibliography

1. Nespoulet R. Le Périgordien VI de l'abri Pataud, Les Eyzies-de-Tayac, Dordogne. L'étude technologique et typologique de l'industrie lithique de la couche 3. Muséum national d'histoire naturelle. 1996.
