# Supplementary material for: Why did hunting weapon design change at Abri Pataud? Lithic use-wear data on armature use and hafting around 24,000–22,000 BP
Source: PLoS One. 2022 Jan 14;17(1):e0262185. doi: 10.1371/journal.pone.0262185 (PMC8759672; doi:10.1371/journal.pone.0262185)
Supplement: S5 Appendix — Length of bending break propagation and secondary scars plotted against artefact width. (PDF) [file pone.0262185.s005.pdf]

Why did hunting weapon design change at Abri Pataud?

Noora Taipale, Laurent Chiotti, Veerle Rots

Supporting information

S5 Impact fracture length in Level 3 sample

The measurement data indicates that artefact dimensions (for which width was used as a proxy) are not a straightforward predictor of propagation length in either of the categories of features (Fig S5.1 and Fig S5.2). When bending breaks are examined, the longest propagations occur on some of the smallest-sized points in the assemblage. Both of the exceptionally long breaks are edge-to-edge breaks on microgravettes (see Fig 12 in main text). It seems reasonable to suspect that the length of the propagation is due to the combined effect of a strong compressive force on impact and the presence of a guiding ridge (i.e. a thin lateral edge) that has allowed a long, narrow termination to form. If these outliers are excluded, the rest of the group appears to show rather constant values instead of a clearly increasing trend with increasing width.

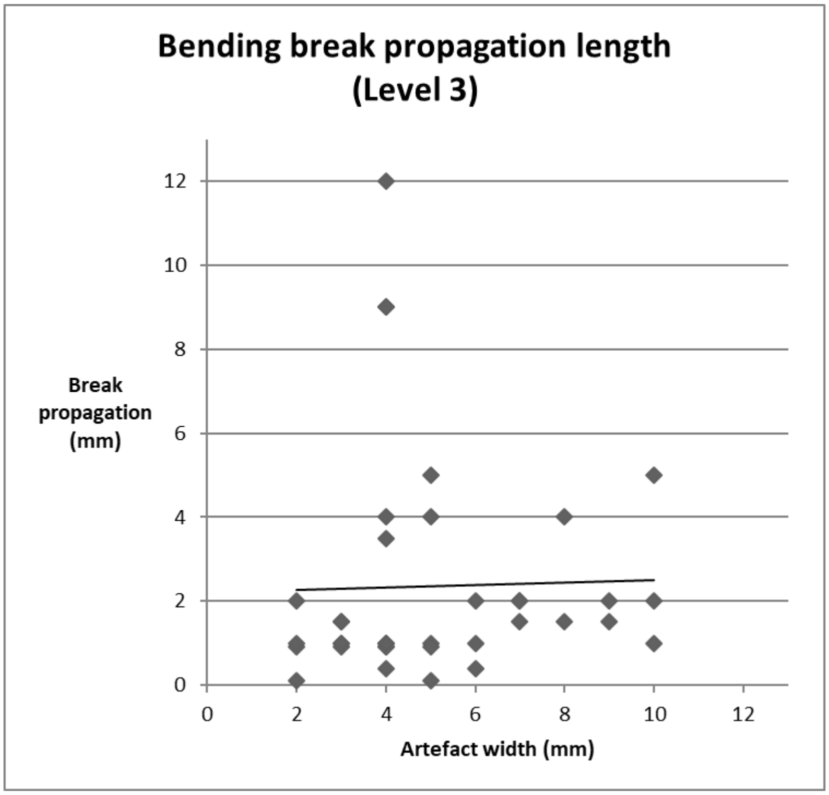

Fig S5.1 *Bending break propagation length (excluding snap breaks) plotted against artefact width in the Abri Pataud Level 3 sample of projectiles (number of observations = 60). Linear trendline is shown. The values are discontinuous because the lengths were measured at 1mm accuracy.*

This is also the case for the length of secondary removals that mostly cluster below the 2mm limit. The rising trend in this sample is affected by the biggest outlier combined with the limited number of observations at the higher end of the scale. The scar measuring 12mm in length is a secondary removal that would be called a (secondary) burination in traditional terminology. What sets it apart from most of the sample is the location of its termination on a lateral edge rather than on a flat surface or dorsal ridge.

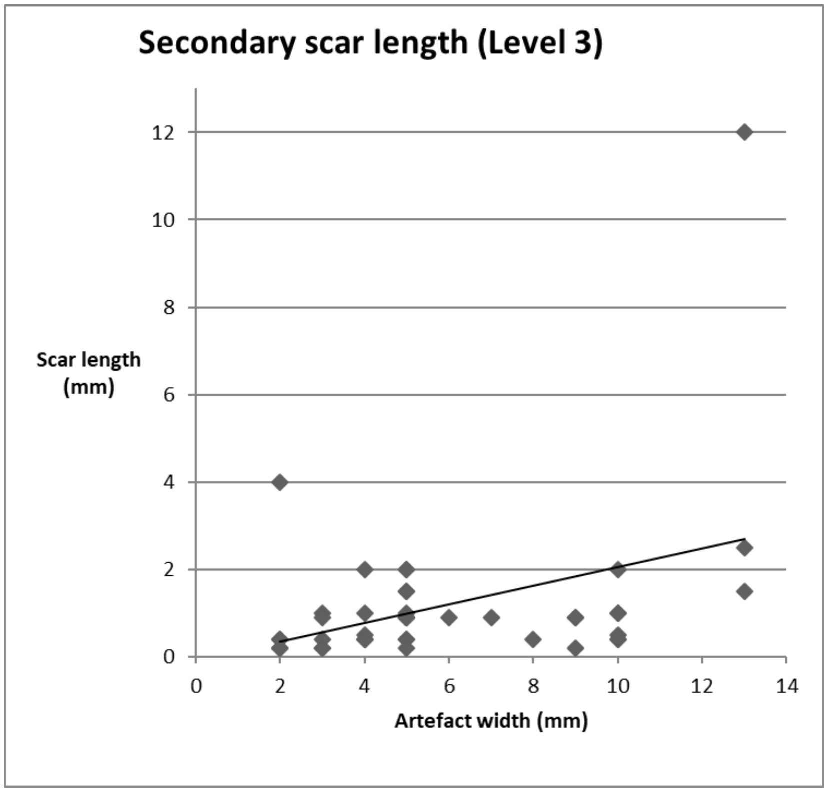

Fig S5.2 *Length of secondary scars plotted against artefact width (proxy for overall size) in the Level 3 projectile sample (number of observations = 44). Linear trendline is shown. The values are discontinuous because the lengths were measured at 1mm accuracy.*

These patterns are of interest because they confirm the observation made elsewhere that armature morphology plays a central role in impact fracture formation [1–3]. If the variation introduced by the location of termination could be filtered out of the data, the size of the features could potentially tell more about the load conditions affecting the lithic armature than about its overall size. This could be tested by comparing similar features (e.g. secondary scars with their termination on a dorsal ridge) with each other. It needs to be remembered that armature breakage can be a rather complex event. The lack of clear correlation between armature size and feature size here can have partly to do with the fact that part of the largest Gravette points show multiple breaks, which means that the energy involved in the impact has

caused more than one fracture to form simultaneously. Logically speaking, the higher the number of breaks, the less energy there will be available per break, leading into shorter propagations in the case of multiple breaks. The data here would have to be normalised by taking this effect into account before drawing further conclusions.

Bibliography

1. O'Farrell M. Approche technologique et fonctionnelle des pointes de la Gravette : une analyse archéologique et expérimentale appliquée à la collection de Corbiac (Dordogne). Université de Bordeaux I. 1996.
2. Rots V, Plisson H. Projectiles and the abuse of the use-wear method in a search for impact. J Archaeol Sci. 2014;48: 154–165. doi:10.1016/j.jas.2013.10.027
3. Coppe J. Sur les traces de l'armement préhistorique : mise au point d'une méthode pour reconstruire les modes d'emmanchement et de propulsion des armatures lithiques par une approche expérimentale, mécanique et balistique. Université de Liège. 2020.
