# Supplementary material for: Why did hunting weapon design change at Abri Pataud? Lithic use-wear data on armature use and hafting around 24,000–22,000 BP
Source: PLoS One. 2022 Jan 14;17(1):e0262185. doi: 10.1371/journal.pone.0262185 (PMC8759672; doi:10.1371/journal.pone.0262185)
Supplement: S6 Appendix — Description of potential impact-related damage. (PDF) [file pone.0262185.s006.pdf]

Why did hunting weapon design change at Abri Pataud?

Noora Taipale, Laurent Chiotti, Veerle Rots

Supporting information

S6 Damage on two Level 2 denticulated backed pieces

The larger of the denticulated pieces showed relatively subtle damage consisting of obliquely oriented, shallow, and rather invasive ventral removals at the tips of the teeth of the denticulated edge. The initiations of some of these scars have been removed with dorsal retouch while others show preserved bending initiations (Fig S6). The removed initiations suggest that the scars are either production-related or use damage that predates the final stage of shaping. Their oblique orientation would speak for the latter option. Under high magnification, the tips of the teeth showed only rare tiny spots of polish, and evidence of repetitive cutting motion (knife use) is absent. The proximal extremity shows minor bifacial edge damage. The break at the proximal end is a heavily oblique, ventrally initiated snap with very small secondary scars on the surface of initiation. The other extremity is truncated.

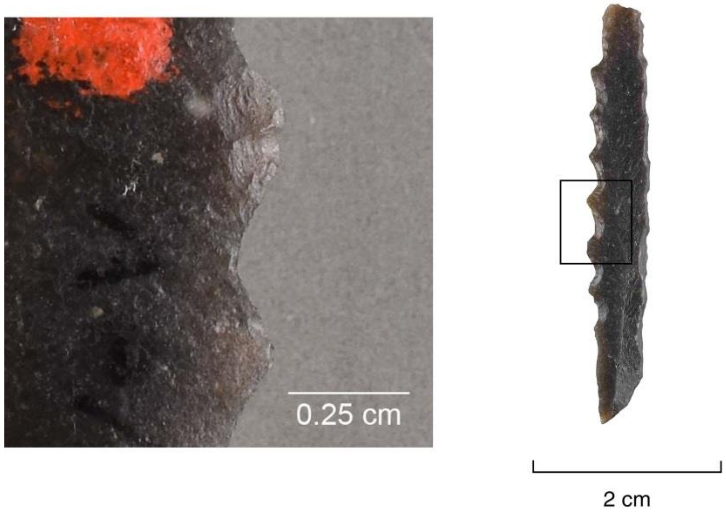

Fig S6 *Subtle lateral removals on the ventral aspect of the denticulated backed piece AP/58-2-736 (Senonian flint).*

The other denticulate (AP/58-2-901) is a short medial fragment. The proximal break is a slightly oblique snap with secondary scars on the surface of termination. These secondary removals are twisted and lack cone initiations. The distal break is a slightly twisted snap that was noted to show possible MLITs under

high magnification, but the surface is altered and the observation remains uncertain. The lateral edge shows minor perpendicularly oriented bending-initiated scars, part of them located on the tips of the teeth.
