# Supplementary material for: Why did hunting weapon design change at Abri Pataud? Lithic use-wear data on armature use and hafting around 24,000–22,000 BP
Source: PLoS One. 2022 Jan 14;17(1):e0262185. doi: 10.1371/journal.pone.0262185 (PMC8759672; doi:10.1371/journal.pone.0262185)
Supplement: S7 Appendix — Evidence of reworking of damaged armatures. (PDF) [file pone.0262185.s007.pdf]

Why did hunting weapon design change at Abri Pataud?

Noora Taipale, Laurent Chiotti, Veerle Rots

Supporting information

S7 Length of armature use

The manufacturing of a backed piece is not a lengthy process once the blanks have been produced. Pétillon et al. mention spending a minimum of one minute on shaping the back of a single insert although they note that the high breakage rate increased the time needed for producing the armatures [1]. Interestingly, some of the inserts examined here show evidence that may be interpreted as reshaping (maintenance) of the small implements even though it was not always easy to ascertain that the remains of previous fractures were due to use and not production. This is the case for a truncated backed piece (AP/63-2-1922) that showed a fissured termination of an earlier break or removal on its ventral surface, cut by subsequent truncating retouch (see Fig S7). Another piece from the same level (AP/58-2-967) showed a possible impact break (a step/fissure-terminated break with about 5mm of its original length preserved) that predated the truncation. Also one of the denticulated backed pieces described previously (AP/58-2-736) showed potential signs of reworking (see S6 Appendix).

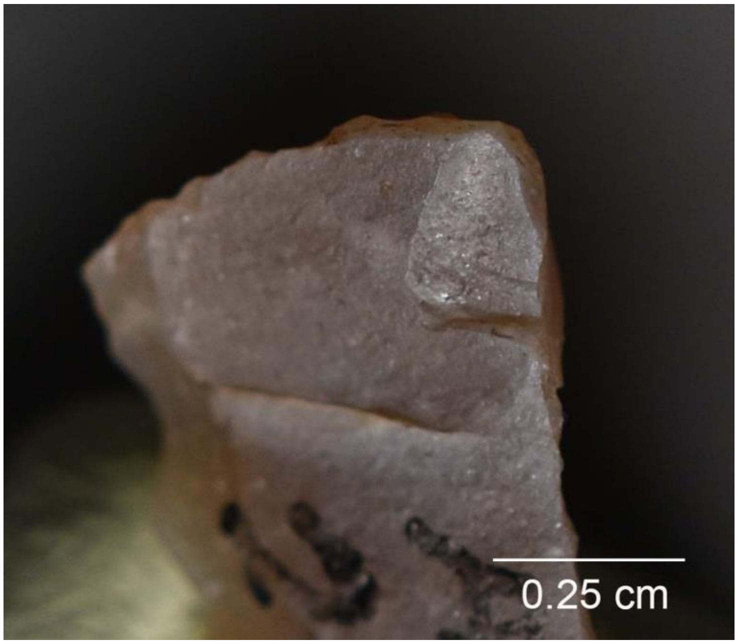

Fig S7 *Preserved fissured step termination of a large removal cut by the truncation on AP/63-2-1922 (Senonian flint). The subsequent smaller longitudinal removal initiated on the truncation may have been caused by contact with the shaft or another barb on impact.*

There are also cases in the Level 2 assemblage where several generations of impact damage appear to overlap. An example is AP/58-2-58, which shows an elongated removal ("burination") that is cut by lateral edge damage and possibly indicates repeated impact. A number of pieces with heavy lateral edge damage were also noted to show obliquely oriented removals in two opposite directions. One explanation for this pattern would be that some of the previously damaged but not yet unusable inserts were removed from their shafts as a part of retooling activities and then re-hafted the other way around. Yet, other explanations for the damage exist, and include barb collision on impact. Also the possibility that part of the damage formed when the weapon (possibly stuck in bone) was pulled out of the carcass with force should be considered. Determining the circumstances under which such bidirectional lateral scarring can occur would need further experimental work.

In the Level 3 sample, evidence of reshaping is scarce, but one armature (AP/58-3-1236) whose morphology is somewhat unusual showed possible evidence of reshaping of a broken end by truncating. Also the distal tip of the implement is rather robust compared to some others, which might mean it was also reworked.

Even though the evidence presented here can be considered preliminary, it is possible that at least some lithic armatures remained in use over slightly extended periods of time.

Bibliography

1. Pétillon J-M, Bignon O, Bodu P, Cattelain P, Debout G, Langlais M, et al. Hard core and cutting edge: Experimental manufacture and use of Magdalenian composite projectile tips. J Archaeol Sci. 2011;38: 1266–1283. doi:10.1016/j.jas.2011.01.002
