# Supplementary material for: Why did hunting weapon design change at Abri Pataud? Lithic use-wear data on armature use and hafting around 24,000–22,000 BP
Source: PLoS One. 2022 Jan 14;17(1):e0262185. doi: 10.1371/journal.pone.0262185 (PMC8759672; doi:10.1371/journal.pone.0262185)
Supplement: S8 Appendix — Comparison of relative frequencies of main impact fracture categories in the Level 2 subsamples and complete sample. (PDF) [file pone.0262185.s008.pdf]

Why did hunting weapon design change at Abri Pataud?

Noora Taipale, Laurent Chiotti, Veerle Rots

Supporting information

S8 Impact fracture frequencies in the Level 2 sample

Fig S8 and Table S8 show that differences in relative frequencies of main impact fracture categories are minor when different parts of the sample (truncated and non-truncated artefacts) are contrasted with each other. The differences observed are mainly due to the higher number of bending breaks (including snap breaks) in the sample of tools where neither of the extremities is truncated. Because the attributes of the breaks themselves do not easily allow separating between production and use-related breaks (see main text), all breaks are included here. Due to the insignificant differences between the two subsamples, the Level 2 sample is treated as a single whole.

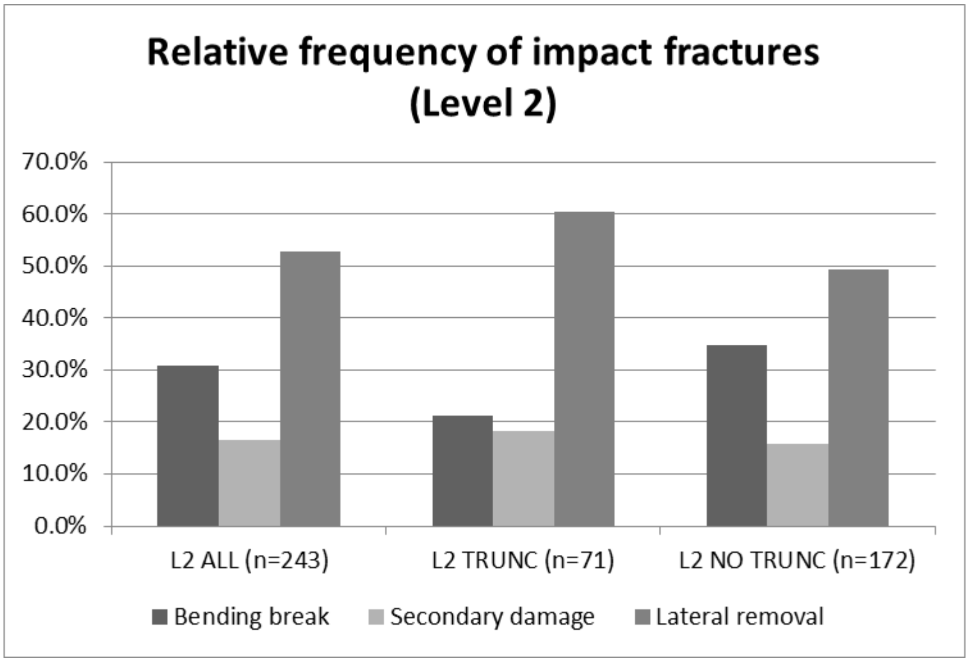

Fig S8 *Comparison of relative frequencies of main impact fracture categories in the Level 2 subsamples and complete sample. Counts in brackets refer to the number of features. Artefact counts are shown in Table S8.1.*

Table S8 *Data for Fig S8.1. Sample sizes in brackets refer to the number of artefacts.*

	L2 TRUNCATED (n=15)		L2 NO TRUNCATION (n=32)		L2 ALL (n=47)	
	n features	% features	n features	% features	n features	% features
Bending break	15	21.1%	60	34.9%	75	30.9%
Secondary damage	13	18.3%	27	15.7%	40	16.5%
Lateral removal	43	60.6%	85	49.4%	128	52.7%
Total	71	100.0%	172	100.0%	243	100.0%
