# Supplementary material for: Why did hunting weapon design change at Abri Pataud? Lithic use-wear data on armature use and hafting around 24,000–22,000 BP
Source: PLoS One. 2022 Jan 14;17(1):e0262185. doi: 10.1371/journal.pone.0262185 (PMC8759672; doi:10.1371/journal.pone.0262185)
Supplement: S9 Appendix — (PDF) [file pone.0262185.s009.pdf]

# Why did hunting weapon design change at Abri Pataud?

Noora Taipale, Laurent Chiotti, Veerle Rots

# Supporting information

## **S9** Data for Fig 18

Table S9 *Data for Fig 18 (main text). Counts are features recorded on the projectiles (Gravette: n=4, microgravette: n=18, nanogravette: n=7, truncated: n=4, indet: n=7, Level 2: n=47)*

| **Impact damage category** | **L3 Gravette** | | **L3 Micrograv.** | | **L3 Nanograv.** | | **L3 Truncated** | | **L3 Indet** | | **L2** | |
|---|---|---|---|---|---|---|---|---|---|---|---|---|
| | **n** | **%** | **n** | **%** | **n** | **%** | **n** | **%** | **n** | **%** | **n** | **%** |
| Bending break | 8 | 33,3% | 30 | 39,5% | 13 | 37,1% | 2 | 22,2% | 11 | 32,4% | 75 | 30,9% |
| Secondary damage | 12 | 50,0% | 16 | 21,1% | 6 | 17,1% | 1 | 11,1% | 11 | 32,4% | 40 | 16,5% |
| Lateral removal | 4 | 16,7% | 30 | 39,5% | 16 | 45,7% | 6 | 66,7% | 12 | 35,3% | 128 | 52,7% |
| Total | 24 | 100,0% | 76 | 100,0% | 35 | 100,0% | 9 | 100,0% | 34 | 100,0% | 243 | 100,0% |
