# Supplementary material for: Why did hunting weapon design change at Abri Pataud? Lithic use-wear data on armature use and hafting around 24,000–22,000 BP
Source: PLoS One. 2022 Jan 14;17(1):e0262185. doi: 10.1371/journal.pone.0262185 (PMC8759672; doi:10.1371/journal.pone.0262185)
Supplement: S10 Appendix — Discussion of the strength of glue and other aspects of projectile hafting arrangements based on archaeological impact damage intensity and experimental observations. (PDF) [file pone.0262185.s010.pdf]

Why did hunting weapon design change at Abri Pataud?

Noora Taipale, Laurent Chiotti, Veerle Rots

Supporting information

S10 Haft raw materials

In the moment of impact, the kinetic energy released results in the breakage of the weakest element of the weapon or the target unless all the energy is dissipated in elastic deformation of the target [1,2]. The strength of the hafting arrangement therefore affects impact damage characteristics since breakage of the shaft or detachment of the lithic armature reduce the amount of damage suffered by the armature. Previous experiments have reported lighter and/or less frequent damage on laterally hafted armatures as opposed to distally hafted ones [3–5]. Some of this variation can be attributed to the frequent detachment of the laterally hafted elements on impact, i.e. failure of the hafting arrangement, although armatures coming into contact with each other on such an event can cause characteristic forms of damage [4–9].

In this respect, the often very heavy damage present in the form of breaks as well as invasive lateral scarring in the Level 2 sample is particularly noteworthy. The impact damage is sometimes so heavy that not much else than the back is left of the original armature (Fig S10). For such extreme forms of damage to occur, the armature needs to be well attached to the body of the composite point. This means that the hafting adhesive (glue) used has been very strong. Finding a good recipe for hafting glue requires striking a balance between different mechanical properties. The glue should be strong but not too brittle, which is the reason why natural resins (pine, spruce) often benefit from being mixed with, for instance, beeswax [10,11]. Previous experimental results also indicate that the amount of glue applied is an important variable in terms of how well the armature adheres to its shaft [9]. Judging from the extreme forms of damage documented in the Level 2 sample, the makers of the hunting weapons had more or less reached an ideal balance with regard to either glue composition or the amount of adhesive used, or, more probably, both. The Level 3 sample is much more varied in terms of artefact shapes and sizes, and putting together a set of armatures comparable to that from Level 2 is not easy. Nevertheless, individual pieces such as the truncated backed bladelet 847 and the nanogravette 1210 (see Fig 13 main text) show rather intense forms of lateral edge damage, suggesting that the expertise in the manufacture of composite points was already in place during the time of the Level 3 occupations.

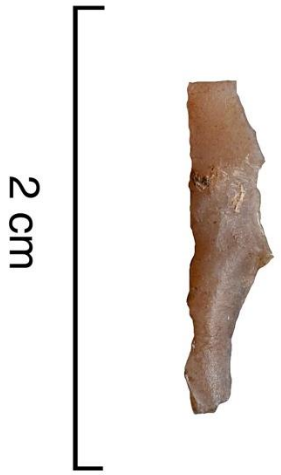

Fig S10 *Heavily damaged armature AP/58-2-800 of which practically only the back remains.*

Level 3 and Level 2 have both yielded pointed osseous tools conceived as spear or dart heads, Level 3 in antler and ivory and Level 2 in antler [12]. It is tempting to picture these as the main components of composite points on which lithic armatures were hafted. While the use of the more perishable wood should not be ruled out, climatic and environmental reconstructions point to cold conditions and an open landscape, which would have made wood scarcely available. During the analysis of the Level 2 assemblage, rare artefacts with dark residues were noted that should be examined in further detail for a possible presence of organic hafting remains, particularly since non-destructive methods for identifying different kinds of adhesives are in development [13,14]. Until such analyses can be conducted, however, it is crucial to bear in mind that different taphonomic processes can produce deposits that can appear glue or resin-like when examined with optical microscopy, that actual adhesive residues can be transformed as the result of taphonomic processes, and that the risk of misidentification of residues is high when relying on visual criteria alone [15,16]. Yet, previous work has demonstrated that despite having been washed and intensively handled, the lithic tools in the Movius collection have preserved some of the more durable functional residues [17], which means that this collection may hold potential for detailed residue analysis.

For Gravette and microgravette points, good results have been achieved experimentally by using a combination of resin and bindings (e.g. sinew) for fixing the points firmly in their shafts [18–20]. The experiments conducted at TraceoLab [20,21] employed split wooden shafts, but also hafting in a groove of a wooden shaft has been successfully tested [19]. The breakage patterns, particularly the near total destruction of some of the largest Gravette points, are consistent with resistant hafting. At the moment not much more than this can be said; an attempt was made to detect hafting wear on Gravettes, microgravettes

and truncated backed pieces under high magnification, but this effort was soon abandoned since it did not yield any convincing results. So far, the only evidence that could have directly to do with the contact of the armatures with their shaft consists of rare removals on the side of the backed edge on some of the Level 2 backed pieces, and as noted above, these features may alternatively be the result of lateral inserts coming into contact with each other on impact.

Bibliography

1. Plisson H, Beyries S. Pointes ou outils triangulaires ? Données fonctionnelles dans le Moustérien levantin [suivi des] Commentaires de J. Shea, A. Marks, J-M Geneste et de la réponse des auteurs. Paléorient. 1998;24: 5–24. doi:10.3406/paleo.1998.4666
2. Rots V, Plisson H. Projectiles and the abuse of the use-wear method in a search for impact. J Archaeol Sci. 2014;48: 154–165. doi:10.1016/j.jas.2013.10.027
3. Crombé P, Perdaen Y, Sergant J, Caspar J. Wear Analysis on Early Mesolithic Microliths from the Verrebroek Site, East Flanders, Belgium. J F Archaeol. 2001;28: 253–269. doi:10.1179/jfa.2001.28.3-4.253
4. Chesnaux L. Réflexion sur le microlithisme en France au cours du premier Mésolithique Xe-VIIIe millénaires avant J.-C.: approches technologique, expérimentale et fonctionelle. Université de Paris 1 Panthéon-Sorbonne. 2014.
5. Rots V. Projectiles and hafting technology. In: Iovita R, Sano K, editors. Multidisciplinary Approaches to the Study of Stone Age Weaponry. Springer; 2016. pp. 167–185. doi:10.1007/978-94-017-7602-8
6. Moss EH, Newcomer MH. Reconstruction of Tool Use at Pincevent: Microwear and Experiments. Tailler! Pourquoi faire: Préhistoire et technologie lithique II, Recent Progress in Microwear Studies Studia Praehistorica Belgica Leuven 2. 1982. pp. 289–312.
7. de la Peña P, Taipale N, Wadley L, Rots V. A techno-functional perspective on quartz micro-notches in Sibudu's Howiesons Poort reveals the use of barbs in hunting technology. J Archaeol Sci. 2018;93: 166–195. doi:10.1016/j.jas.2018.03.001
8. Taipale N, Rots V. Breakage, scarring, scratches and explosions: understanding impact trace formation on quartz. Archaeol Anthropol Sci. 2018;11: 3013–3039. doi:10.1007/s12520-018-0738-z
9. Pétillon J-M, Bignon O, Bodu P, Cattelain P, Debout G, Langlais M, et al. Hard core and cutting edge: Experimental manufacture and use of Magdalenian composite projectile tips. J Archaeol Sci. 2011;38: 1266–1283. doi:10.1016/j.jas.2011.01.002
10. Gaillard Y, Chesnaux L, Girard M, Burr A, Darque-Ceretti E, Felder E, et al. Assessing Hafting Adhesive Efficiency in the Experimental Shooting of Projectile Points: A new Device for Instrumented and Ballistic Experiments. Archaeometry. 2015;58: 465–483. doi:10.1111/arcm.12175
11. Rots V. Hafting Traces on Flint Tools: Possibilities and Limitations of Macro- and Microscopic Approaches. Katholieke Universiteit Leuven. 2002.
12. Bricker HM. Le paléolithique supérieur de l'abri Pataud (Dordogne): les fouilles de H. L. Movius Jr. Paris: Éditions de la Maison des Sciences de l'Homme; 1995.
13. Perrault KA, Stefanuto P-H, Dubois L, Cnuts D, Rots V, Focant J-F. A New Approach for the Characterization of Organic Residues from Stone Tools Using GC×GC-TOFMS. Separations. 2016;3: 16. doi:10.3390/separations3020016
14. Perrault KA, Dubois LM, Cnuts D, Rots V, Focant J-F, Stefanuto P-H. Characterization of hafting adhesives using comprehensive two-dimensional gas chromatography coupled to time-of-flight mass spectrometry. Sep Sci Plus. 2018;1: 726–737. doi:10.1002/sscp.201800111
15. Cnuts D, Tomasso S, Rots V. The Role of Fire in the Life of an Adhesive. J Archaeol Method Theory. 2018;25:

839–862. doi:10.1007/s10816-017-9361-z
16. Rots V, Hayes EH, Cnuts D, Lepers C, Fullagar R. Making sense of residues on flaked stone artefacts: Learning from blind tests. PLoS One. 2016;11: 1–38. doi:10.1371/journal.pone.0150437
17. Taipale N. Hafting as a flexible strategy: variability in stone tool use and hafting at three European Upper Palaeolithic sites. University of Liège. 2020.
18. Cattelain P, Perpère M. Tir expérimental de sagaies et de flèches emmanchées de pointes de la Gravette. Archéo-Situla. 1993;17–20: 5–28.
19. Soriano S. Les microgravettes du Périgordien de Rabier à Lanquais (Dordogne) : analyse technologique fonctionnelle. Gall préhistoire. 1998;40: 75–94. doi:10.3406/galip.1998.2158
20. Coppe J. Sur les traces de l’armement préhistorique : mise au point d’une méthode pour reconstruire les modes d’emmanchement et de propulsion des armatures lithiques par une approche expérimentale, mécanique et balistique. Université de Liège. 2020.
21. Coppe J, Rots V. Focus on the target. The importance of a transparent fracture terminology for understanding projectile points and projecting modes. J Archaeol Sci Reports. 2017;12: 109–123. doi:10.1016/j.jasrep.2017.01.010
